# Surface Potential and Surface Dipole Moment of Water and Polar-Quadrupolar Liquids

**DOI:** 10.1021/acs.langmuir.6c00269

**Published:** 2026-06-30

**Authors:** Radomir I. Slavchov, Boyan Peychev, Iglika M. Dimitrova

**Affiliations:** 1 School of Engineering and Materials Science, 4617 Queen Mary University of London, Mile End Road, London E1 4NS, U.K.; 2 Institute of Physical Chemistry, Bulgarian Academy of Sciences, Akad. G. Bonchev Str., bl. 11, Sofia ZIP 1113, Bulgaria; 3 Faculty of Chemical Technologies, Department of Physical Chemistry, 112623 University of Chemical Technology and Metallurgy, 8, Kliment Ohridski blvd, ZIP 1756 Sofia, Bulgaria

## Abstract

Nearby interfaces, polar-quadrupolar molecules are orientated by image forces. This produces a macroscopic normal dipole moment and is the reason for the surface potential of liquids like water. The theory of the effect requires higher-order multipole expansion of both the image interactions and the macroscopic equations of electrostatics. The quadrupolar Coulomb law predicts the formation of a dipolar double layer (DDL) at the surface, with an adsorbed layer of dipoles orientated by image forces and oppositely polarized diffuse layer formed as a response to the field of the adsorbed dipoles. We relate the surface dipolar potential to the molecular properties (dipole and quadrupole moments and molecular polarizability), the medium properties (dielectric permittivity and macroscopic quadrupolarizability), and structural characteristics of the surface layer (distance between equimolecular and dielectric surfaces, thickness of the adsorbed layer, and cavity size). The thickness of the DDL is set by the quadrupolar length of the fluid, a quantity analogous to the Debye length. Molecules on the gas-phase side of the Gibbs equimolecular surface have a large contribution to the adsorbed dipole, especially for water. The surface layer is thick enough to produce a diffuse dipole layer also on the gas-phase side of the surface, i.e., three layers of dipoles exist, in agreement with recent simulations data. The calculated unperturbed surface polarization of the adsorbed layer for water agrees with estimates from point of zero dipole of alcohol monolayers. The calculated surface potential of water (between −15 and −85 mV, depending on the assumptions; oxygen toward the gas phase) agrees with existing experimental estimates.

## Graphical abstract

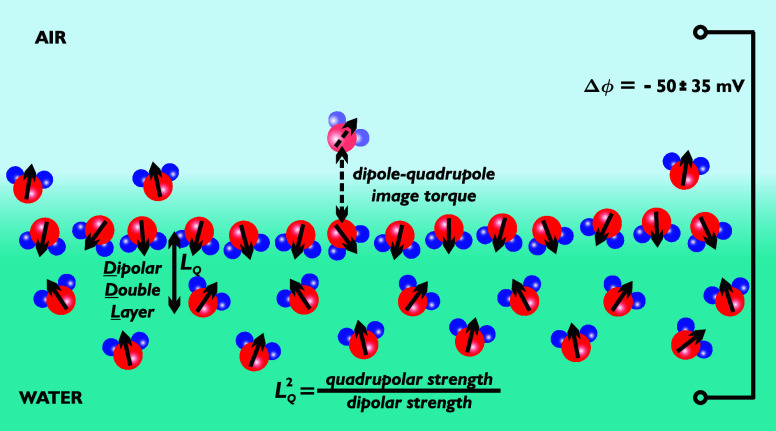

## Introduction

All surfaces carry a dipole moment. The anisotropy of the surface layer causes a spontaneous orientation of the molecules in it, even in the absence of an external electric field. The polarization of the surface layer has a complex distribution analogous to the electric double layer (EDL): it includes an *adsorbed layer of dipoles* (of density *P* ^S^ [C/m]) orientated by some specific forces, and a *diffuse layer of dipoles* oppositely orientated by the electric field of the adsorbed layer.^1^ The thickness of this *dipolar double layer* (DDL) is set by the so-called *quadrupolar length L*_*Q*_ – a material characteristic determined by the ratio between the quadrupolar and the dipolar strengths of the bulk phase, analogous to the Debye length.^1^ The total surface dipole moment Γ_*P*_ is the sum of the specifically adsorbed dipole *P* ^S^ and the diffuse dipole, and |Γ_*P*_| < |*P* ^S^| (see list of symbols and abbreviations in Supplementary Information S1).

The surface dipole moment is involved in many surface phenomena. First of all, it is the source of the surface potential:^2^

$$\Delta_{L}^{G}\phi =\Gamma_{P}/\varepsilon_{0}$$ 1

The surface potential produces large fields nearby any three-phase contact.^2,3^ The change of Δ_L_^G^ϕ upon spreading an insoluble monolayer onto a surface (called the volta potential Δ*V* of the monolayer) can be measured with a Kelvin probe, which is a standard technique for characterization of surfactant films.^4^ The change of Δ_L_^G^ϕ upon adding electrolyte in the bulk (the Δχ potential) is a valuable source of information for the interaction of ions with aqueous interfaces.^5–7^ In heterogeneous monolayers, the variance of the normal dipole density produces long-range fields responsible for fascinating macroscopic structures.^8^ Γ_*P*_ influences the dispersion of thermal capillary waves.^9^ The free energy of the DDL contributes to the surface tension.^1^ Γ_*P*_ contributes also to the nucleation work for a liquid droplet on charged particles^10^ and is important for nucleation phenomena in the atmosphere. If the bulk phase is an electrolyte solution, the interaction of the electrolyte with the DDL produces ionic surface charge.^11^ If a clean surface carries a dipole moment, one can alter it via adsorption of surfactant that carries oppositely orientated dipole; by adsorbing the right amount of such surfactant, we can achieve a surface of zero dipole moment^11^ – that is, there exists a *point of zero dipole* (pzd). In a thin liquid film of a quadrupolar phase between two polar phases, like water|benzene|water or water|dense-phase CO_2_|water, the surface potential and the DDL can give rise to an electrostatic disjoining pressure (a non-DLVO surface force corresponding to adsorbed dipole-adsorbed dipole interaction); to our knowledge, this force has not been investigated yet but can be inferred directly from the form of the Maxwell tensor in quadrupolar media.^1^ The absolute single-ion hydration free energy involves, by definition, a contribution from Δ_L_^G^ϕ, and also from the similar jump of the potential at the surface between the ion cavity and the aqueous phase.^12,13^

The source of the surface orientation varies from system to system. Polar molecules with an alkyl chain at the liquid water|gas surface (L|G) are orientated by the hydrophobic effect.^4^ The surface of a metal that is terminated by hydroxyl groups carries a large dipole moment produced by the orientation of the chemical bond between the metal and –OH. Molecules adsorbed on solids are often orientated by hydrogen bonding. For the simplest polar liquids, however, the source of the surface dipole moment is not very well understood. For example, pure water is known to carry a negative surface potential^14,15^ (water in the surface layer is orientated with the oxygen atom toward the gas phase^16,17^ ). Frenkel^18^ suggested the qualitative explanation of this orientation: that it is due to an image force acting on a dipole–quadrupole molecule. However, 70 years later, this idea has not progressed much. So far, it has been shown to be valid for a dipole–quadrupole fluid near its critical point by Stillinger and Ben Naim;^19^ furthermore, Croxton^20^ used a variational method to estimate the distribution of the dipole moment near the surface of water. The strong link between the molecular quadrupole moment and the surface orientation was demonstrated also through molecular dynamics (MD) simulations.^21^

There are several difficulties that complicate the analysis of Frenkel’s hypothesis:

1. The classical expression for the orientating dipole–quadrupole image potential has a singularity at the surface (it diverges as 1/*z*_o_^4^ where *z*_o_ is the distance between the molecule and the surface).
2. The distance *z*_o_ is not straightforward to define – the location of the ‘dielectric surface’ does not coincide with the equimolecular surface.^22^
3. The value of the quadrupole moment of a dipole–quadrupole particle depends on the origin.^19^ It is not always obvious which is the suitable origin.
4. The image field is strong and leads to a nonlinear Boltzmann distribution and molecular depolarization effects. Water is a particularly complicated case.
5. There are also complex macroscopic depolarization effects producing the diffuse dipolar layer structure.^1^ These require nonlocal approach to be handled, such as the equation of quadrupolar electrostatics (an extension of the macroscopic Poisson equation of electrostatics to include medium quadrupolarizability and adsorbed surface dipole).
6. Molecules in the gas phase well above the equimolecular surface contribute^20^ to the adsorbed dipole *P* ^S^.
7. A quadrupole of nonzero trace produces a Bethe potential^23^ – a bulk phenomenon distinct from the surface potential which has caused some confusion in the literature, especially in MD studies. The Bethe potential is a bulk quantity of little importance for electrochemical systems.^1,12^

8. Finite size effects, reaction field and cavity field effects can be important.^19^
9. Electrolytes have a complex effect on the Δ_L_^G^ϕ potential;^6,24^ measuring the changes in Δ_L_^G^ϕ, however, generally requires electrolyte to be present.
10. For interactions as short-ranged as the dipole–quadrupole image potential, the quadrupolar response of the medium becomes important, i.e. the gradient of the macroscopic quadrupolarizability also produces image forces.

We will not consider items (viii-x) in detail in this work. In a companion paper, we will investigate image forces on ion-dipole near a surface, where screening, i.e. item (ix), is essential. As for item (viii), to deal with the finite size effects, we will introduce a cutoff parameter and will investigate its role; we will also provide an approximate analysis of the reaction and cavity field effects.

While the change of Δ_L_^G^ϕ is readily accessible, its absolute value is not directly measurable. Attempts to determine it have been made by a stellar team of electrochemists (see reviews by Conway,^25^ Paluch,^14^ and Parfenyuk^15^). Our group attempted to determine it from indirect data for Δχ potentials of simple electrolytes.^24^ Simulations show sensitivity to the force model.^16,17,21,26^ The various methods produce values that vary by both order of magnitude and sign; as a result, the electrochemical community has progressed little from the vague estimate of Frumkin et al.,^27^ that Δ_L_^G^ϕ is something like −100···-200 mV, oxygen pointing toward the gas phase.

Below, we present a general theory of the dipolar surface potential of simple polar liquids produced by image forces. The electrostatic theory of the interaction of a multipole with an interface is presented first. The resulting statistical distribution of the orientation is analyzed next. Finally, the adsorbed dipole *P* ^S^, the total surface dipole Γ_*P*_ and the surface potential Δ_L_^G^ϕ are calculated and compared to existing experimental and molecular dynamics simulation results.

## Theoretical Section

### Field of a Point Source Near an Interface

Let a point charge *e* be located at the origin ***r***_o_ = (*x*_o_, *y*_o_, *z*_o_) in ‘liquid’ phase (L, *z* > 0, dielectric permittivity ε^L^), at a distance *z*_o_ away from the boundary *z* = 0 with the ‘gas’ (G, *z* < 0, permittivity ε^G^, Figure 1). The electrostatic potential of *e* in phase L is given by the expression:^28^

$$\phi_{e}^{L}=\phi_{e}+\phi_{e,\mathrm{im}},\mathrm{where}\;\phi_{e}=\frac{e}{4\pi \varepsilon^{L}}\frac{1}{\left|r-r_{o}\right|}\;\mathrm{and}\;\phi_{e,\mathrm{im}}=\frac{e_{\mathrm{im}}}{4\pi \varepsilon^{L}}\frac{1}{\left|r-r_{\mathrm{im}}\right|}$$ 2

The first term, ϕ_*e*_, stands for the potential of the ion itself, without the perturbation caused by the interface. The second term, ϕ_*e*,im_, is the image potential corresponding to the field reflected by the interface. The magnitude *e*_im_ and the apparent location ***r***_im_ of the image charge are given by^28^

$$e_{\mathrm{im}}=\frac{\varepsilon^{L}-\varepsilon^{G}}{\varepsilon^{L}+\varepsilon^{G}}e,r_{\mathrm{im}}=\left(x_{o},y_{o},-z_{o}\right)$$ 3

see Figure 1. One important question regarding the definition of *z*_o_ is the exact location of the boundary between the two phases: the surface layer is a three-dimensional object (*interphase*); hence, various options exist to place this boundary. The option that produces the simplest description of the image forces is the *dielectric interface* – this is where the *excess tangential surface polarizability α*_t_^S^ is zero^22^ (i.e., no surface excess of ε). At any other interface, the surface polarizability modifies the image force;^29^ the leading correction for the image energy is O­(*z*_o_^–2^), see the SupplementS2.

**Figure 1:**
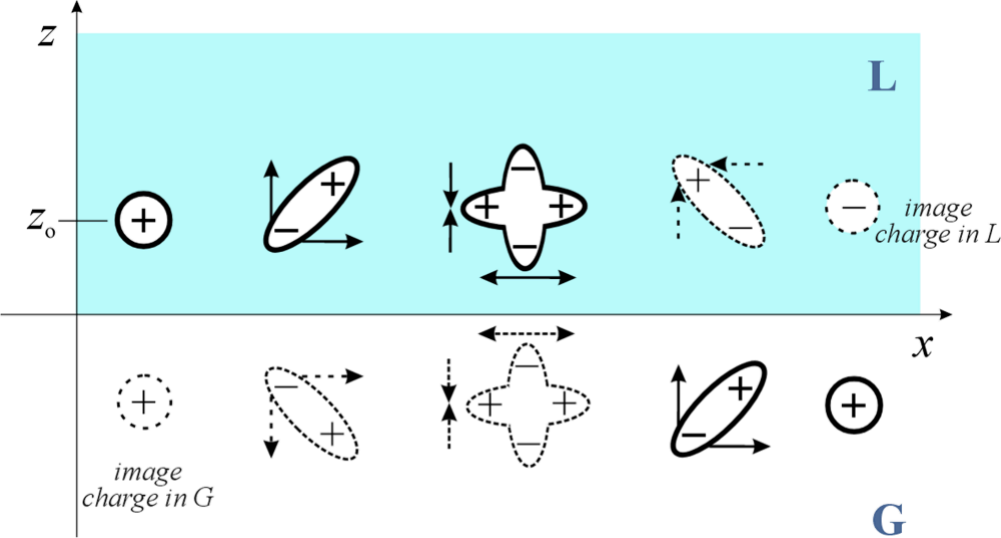
Images of a charge, a dipole, and a *q*_*xx*_ + *q*_*zz*_ quadrupole in the liquid (L) phase and of a dipole and a charge in the gaseous (G) phase (dielectric permittivity ε^L^ > ε^G^). The interface reflects the field of a particle in L with reversal of direction corresponding to same-charge images; for particles in G, the images are of inversed charge.

The potential of a dipole ***p*** near an interface is easily obtained from eq 2 via the *e* ^–1^ ***p*** ·∇_o_ operator:^28^

$$\phi_{p,\mathrm{im}}=k_{\mathrm{im}}^{L}p\cdot \nabla_{o}\frac{1}{\left|r-r_{\mathrm{im}}\right|}=\frac{p_{\mathrm{im}}\cdot \left(r-r_{\mathrm{im}}\right)}{4\pi \varepsilon^{L}{\left|r-r_{\mathrm{im}}\right|}^{3}}$$ 4

where

$$k_{\mathrm{im}}^{L}=\frac{1}{4\pi \varepsilon^{L}}\frac{\varepsilon^{L}-\varepsilon^{G}}{\varepsilon^{L}+\varepsilon^{G}}$$ 5

and

$$p_{\mathrm{im}}=\frac{\varepsilon^{L}-\varepsilon^{G}}{\varepsilon^{L}+\varepsilon^{G}}\left(p_{x},p_{y},-p_{z}\right)$$ 6

The gradient operator ∇_o_ = **e**_*x*_∂/∂*x*_o_ + **e**_*y*_∂/∂*y*_o_ + **e**_*z*_∂/∂*z*_o_ is with respect to the coordinates of the point source; **e**_*i*_ are unit vectors. The dielectric factor *k*_im_^L^ is a measure of the strength of the field reflected by the dielectric surface.

For a point quadrupole ***q*** near an interface, we similarly find

$$\phi_{q,\mathrm{im}}=k_{\mathrm{im}}^{L}q:\frac{1}{2}\nabla_{o}\nabla_{o}\frac{1}{\left|r-r_{\mathrm{im}}\right|}=\frac{3q_{\mathrm{im}}:\left(r-r_{\mathrm{im}}\right)\left(r-r_{\mathrm{im}}\right)}{8\pi \varepsilon^{L}{\left|r-r_{\mathrm{im}}\right|}^{5}}$$ 7

Here, the tensor of the image quadrupole is of magnitude:

$$q_{\mathrm{im}}=\frac{\varepsilon^{L}-\varepsilon^{G}}{\varepsilon^{L}+\varepsilon^{G}}\left(\begin{array}{ccc} q_{xx} & q_{xy} & -q_{xz}\\ & q_{yy} & -q_{yz}\\ & & q_{zz} \end{array}\right)$$ 8

The quadrupole moment is defined here as ***q*** = ∫ρ_molecule_(***rr*** – 1/3 *r* ^2^ **U**)­d*V* where ρ_molecule_ is the charge density of the molecule in a coordinate system where ***r***_o_ = 0. Other definitions of ***q*** are often used in the literature that differ from this one by a factor of 3, 3/2 or 1/2 (e.g., Doyle et al.^13^ use all three). These factors simplify the numerical coefficient in ϕ_*q*_ in Gaussian units or the expression for the displacement field, but complicate, e.g., the definition of a surface quadrupole moment.^1^ Both ***q*** and ***q***_im_ are symmetric (*q*_*ij*_ = *q*_*ji*_) and traceless (*q*_*xx*_ + *q*_*yy*_ + *q*_*zz*_ = 0).

The image potential of a point octupole ***o*** near an interface is given by the expression:

$$\phi_{o,\mathrm{im}}=k_{\mathrm{im}}^{L}o∴\frac{1}{6}\nabla_{o}\nabla_{o}\nabla_{o}\frac{1}{\left|r-r_{\mathrm{im}}\right|}=\frac{5o_{\mathrm{im}}∴\left(r-r_{\mathrm{im}}\right)\left(r-r_{\mathrm{im}}\right)\left(r-r_{\mathrm{im}}\right)}{8\pi \varepsilon^{L}{\left|r-r_{\mathrm{im}}\right|}^{7}}\;\mathrm{where}\;o_{\mathrm{im}}=\frac{\varepsilon^{L}-\varepsilon^{G}}{\varepsilon^{L}+\varepsilon^{G}}\times \left(\begin{array}{ccc} o_{xxx} & o_{xxy} & -o_{xxz}\\ & o_{xyy} & -o_{xyz}\\ & & o_{xzz} \end{array}\left|\begin{array}{ccc} o_{xxy} & o_{xyy} & -o_{xyz}\\ & o_{yyy} & -o_{yyz}\\ & & o_{yzz} \end{array}\right|\begin{array}{ccc} -o_{xxz} & -o_{xyz} & o_{xzz}\\ & -o_{yyz} & o_{yzz}\\ & & -o_{zzz} \end{array}\right)$$ 9

We use the definition *o*_*ijk*_ = ∫ρ_molecule_[*x*_*i*_ *x*_*j*_ *x*_*k*_ – 1/5*r* ^2^(*x*_*i*_δ_*jk*_ + *x*_*j*_δ_*ik*_ + *x*_*k*_δ_*ij*_)]­d*V* of octupole moment (defined with zero ‘traces’, *o*_*xxk*_ + *o*_*yyk*_ + *o*_*zzk*_ = 0 for *k* = *x*,*y*,*z*). Like the quadrupole, several other definitions of ***o*** are in common use; one that differs by a factor of 5/2 is widespread.^28,30^ Also, like ***q***, the octupole moment ***o*** of a polar molecule depends on the origin. The conversion rules for various definitions and origins are summarized in Supplement S4.

### Total Potential and Field

A point dipole–quadrupole-octupole near the dielectric surface has potential of the reflected field given by the sum of eqs 4, 7, and 9:

$$\phi_{\mathrm{im}}=\frac{p_{\mathrm{im}}\cdot \left(r-r_{\mathrm{im}}\right)}{4\pi \varepsilon^{L}{\left|r-r_{\mathrm{im}}\right|}^{3}}+\frac{3q_{\mathrm{im}}:\left(r-r_{\mathrm{im}}\right)\left(r-r_{\mathrm{im}}\right)}{8\pi \varepsilon^{L}{\left|r-r_{\mathrm{im}}\right|}^{5}}+\frac{5o_{\mathrm{im}}∴\left(r-r_{\mathrm{im}}\right)\left(r-r_{\mathrm{im}}\right)\left(r-r_{\mathrm{im}}\right)}{8\pi \varepsilon^{L}{\left|r-r_{\mathrm{im}}\right|}^{7}}$$ 10

The respective image field (***E***_im_ = −∇ϕ_im_) acting at the origin (***r*** = ***r***_o_) is

$$E_{\mathrm{im}}\left(r_{o}\right)=\frac{3p_{\mathrm{im},z}e_{z}-p_{\mathrm{im}}}{4\pi \varepsilon^{L}{\left|2z_{o}\right|}^{3}}+\frac{15q_{\mathrm{im},zz}e_{z}-6q_{\mathrm{im}}\cdot e_{z}}{8\pi \varepsilon^{L}{\left|2z_{o}\right|}^{4}}+\frac{35o_{\mathrm{im},zzz}e_{z}-15o_{\mathrm{im}}:e_{z}e_{z}}{8\pi \varepsilon^{L}{\left|2z_{o}\right|}^{5}}$$ 11

The field gradient ∇ ***E***_im_ and double gradient ∇∇ ***E***_im_ are similarly found (see Supplement S3).

### Energy and Polarization

The image potential acting on a molecule at a distance *z*_o_ from an interface can be expanded into multipolar series. Before writing down this expansion, it is instructive to compare the orders of the terms in it with respect to *z*_o_ (Table 1, cf. ref 32). For an ion of charge *e*, the leading interaction in the multipole expansion is ∼ *e* ^2^/*z*_o_ (charge-image charge); however, it cannot orientate the ion as it is of zero torque. The first term in the expansion that can orientate an ion is ∼ *e* ***p***/*z*_o_^2^ (the sum of the image charge-dipole and charge-image dipole interactions); it orientates ion-dipoles like OH^–^ and H_3_O^+^ – a question that will be considered in a companion paper.

**Table 1:** Order of the Electrostatic Terms in the Multipole Expansion of the Image Potential *u* Acting on a Molecule with Respect to the Distance *z*_o_ between a Point Multipole and the Dielectric Interface

	solid multipole (Keesom-like)					induction (Debye-like)
contributions to *u*	*e*	***p***	***q***	***o***	***h***	α_*p*_, α_*q*_, ···
*e*	*z*_o_^–1^	*z*_o_^–2^	*z*_o_^–3^	*z*_o_^–4^	*z*_o_^–5^	α_*p*_ *e* ^2^ *z*_o_^–4^ + α_*p*_ *e* ***p*** *z*_o_^–5^ + α_*q*_ *e* ^2^ *z*_o_^–6^···
***p***		*z*_o_^–3^	*z*_o_^–4^	*z*_o_^–5^	*z*_o_^–6^	α_*p*_ ***p*** ^2^ *z*_o_^–6^ + α_*p*_ ***pq*** *z*_o_^–7^ + ···
***q***			*z*_o_^–5^	*z*_o_^–6^	*z*_o_^–7^	α_*p*_ ***q*** ^2^ *z*_o_^–8^ + α_*q*_ ***q*** ^2^ *z*_o_^–10^ + ···
***o***				*z*_o_^–7^	*z*_o_^–8^	
***h***					*z*_o_^–9^	

Our focus in this article is on neutral molecules (*e* = 0). For such, the leading term in the multipole expansion of *u* is ∼ ***pp***/*z*_o_^3^ (dipole-image dipole). It alone cannot produce a macroscopic surface dipole moment because it is symmetric – orientations with positive pole up and with negative pole up are equally probable. The most long-ranged term that produces orientation of neutral molecules is ***pq***/*z*_o_^4^ (image dipole–quadrupole plus dipole-image quadrupole), as realized by Frenkel.^18^ In the analysis below, we will also leave in the expansion of *u* all *z*_o_^–5^ terms (***qq*** and ***po***), to analyze the effect of the higher-order terms. The *z*_o_^–5^ interactions are interesting also because they control the orientation of a water molecule when its dipole lies parallel to the surface, which is relevant for surface spectroscopy.^31^ We will also investigate the effect of the molecular polarizability α_*p*_; it gives a *z*_o_^–6^ correction to the expansion of *u* but we will show it modifies the large ***pp*** term through a nonlinear magnification effect.

The *p* ^2^/*z*_o_^3^ term averaged over all orientations produces the electrostatic Keesom potential (and the averaging reduces it to *z*_o_^–6^ at sufficient distance from the surface). The α_*p*_ *p* ^2^/*z*_o_^6^ interaction corresponds to the electrostatic Debye force. The electrodynamic dispersion interaction (dynamic α_*p*_-α_*p*_ interaction) is also *z*_o_^–6^; the dispersion force is the dynamic equivalent of the ***pp*** image force.

The order in Table 1 is valid only for L|G boundary set at the dielectric surface. Any other choice of location of L|G would produce a nonzero tangential surface polarizability, α_t_^S^, leading to additional terms in the multipole expansion, like α_t_^S^ *e* ^2^/*z*_o_^2^, α_t_^S^ *p* ^2^/*z*_o_^4^ etc. We expand on this question in Supplement S2.

### Image Potential for Nonpolarizable Point Multipole

Let us first consider the image energy of a nonpolarizable molecule. At the origin, the image field eq 11 interacts with the dipole ***p***; the field gradient ∇ ***E***_im_ with ***q***, and ∇∇ ***E***_im_ with ***o***. The free energy of the total interaction with the surface is given by^28^

$$u=-\frac{1}{2}p\cdot E_{\mathrm{im}}-\frac{1}{4}q:\nabla E_{\mathrm{im}}-\frac{1}{12}o∴\nabla \nabla E_{\mathrm{im}}$$ 12

where the values of ***E***_im_, ∇ ***E***_im_ and ∇∇ ***E***_im_ are taken at ***r*** = ***r***_o_. Compared to a point multipole in external field, a coefficient 1/2 appears in *u* because the image field is created by the point source itself (i.e., ***E***_im_ ∝ ***p***, ∇ ***E***_im_ ∝ ***q*** etc., hence, the charging procedure for the free energy yields *u* = −∫ ***E***_im_·d ***p*** − ··· = −1/2 ***p*** · ***E***_im_ − ···). With the cross-interactions, one formally has two options: either to use a charging procedure for the multipole particle as a whole or to treat the dipole, the quadrupole, and the octupole as three different particles located in the same origin. If the dipole and the quadrupole are treated as separate particles, the coefficient 1/2 will still appear so that the dipole–quadrupole interaction is not counted twice.

The substitution of eq 10 here (or explicitly, eq 11 and eqs 90 and 91 from Supplement S3) leads to the expression:

$$u_{0}^{L}=\frac{k_{\mathrm{im}}^{L}}{2}\left(\frac{U_{pp0}}{{\left|2z_{o}\right|}^{3}}+\frac{U_{pq}}{{\left|2z_{o}\right|}^{4}}+\frac{U_{qq}+U_{po}}{{\left|2z_{o}\right|}^{5}}\right)$$ 13

where

$$U_{pp0}=p_{0x}^{2}+p_{0y}^{2}+2p_{0z}^{2}$$ 14

$$U_{pq}=-6\left(p_{0x}q_{xz}+p_{0y}q_{yz}\right)-9p_{0z}q_{zz}$$ 15

$$U_{qq}=\frac{3}{4}\left(2q_{xx}^{2}+2q_{yy}^{2}+17q_{zz}^{2}\right)+3q_{xy}^{2}+12\left(q_{xz}^{2}+q_{yz}^{2}\right)$$ 16

$$U_{po}=15\left(p_{0x}o_{xzz}+p_{0y}o_{yzz}\right)+20p_{0z}o_{zzz}$$ 17

The dipole moment ***p***_0_ is in vacuum. The terms ***qo*** and ***oo*** are of order *z*_o_^–6^ and higher; hence, they were neglected in eq 12. In the absence of hexadecapoles, the missing ***ph*** term would anyway make eq 12 of O­(*z*_o_^–6^), see Table 1.

For a molecule in the *gas* phase, the following expression for the image potential can be derived from consideration of symmetry:

$$u_{0}^{G}=\frac{k_{\mathrm{im}}^{G}}{2}\left(-\frac{U_{pp0}}{{\left|2z_{o}\right|}^{3}}+\frac{U_{pq}}{{\left|2z_{o}\right|}^{4}}-\frac{U_{qq}+U_{po}}{{\left|2z_{o}\right|}^{5}}\right)$$ 18

Here, the *U* coefficients are still given by eqs 14–17, and the gas-phase dielectric factor *k*_im_^G^ stands for:

$$k_{\mathrm{im}}^{G}=\frac{1}{4\pi \varepsilon^{G}}\frac{\varepsilon^{L}-\varepsilon^{G}}{\varepsilon^{L}+\varepsilon^{G}}$$ 19

It is larger than *k*_im_^L^ by a factor of ε^L^/ε^G^, see eq 5. Therefore, the molecules on the gas-phase side of the dielectric surface are more strongly orientated than the ones on the liquid-phase side.

The first *z*_o_^–3^ term in the square bracket of eq 13 corresponds to the dipole–dipole image potential *u*_*pp*0_^L^. It pulls the dipole to the polar phase, and the image torque orientates the dipole parallel to the surface (tangential orientation of ***p*** corresponds to half the repulsive energy of the normal orientation, eq 14). However, orientation in direction + **e**_*z*_ and −**e**_*z*_ by this term is equally probable, so *u*_*pp*0_^L^ produces no average normal polarization of the surface. By contrast, from eq 18, for a dipole in the *gaseous* phase, the ***pp***/*z*_o_^3^ term is of negative sign and produces preferential normal orientation of the dipole – but again with no preference for the + **e**_*z*_ or −**e**_*z*_ direction.

The cross-term *u*_*pq*_ ∼ ***pq***/*z*_o_^4^ in eqs 13 and 18 (the sum of the dipole-image quadrupole and quadrupole-image dipole interactions) is the most important interaction for the surface potential, as it produces a mean normal dipole moment. Unlike *u*_*pp*_, it does not switch direction – the produced orientation is the same in the gaseous and the aqueous phases.

However, |*u*_*pq*_^L^| ≪ |*u*_*pq*_^G^|, due to the dielectric factors *k*_im_.

The next term is ***qq***/*z*_o_^5^, the image quadrupole–quadrupole force – again pulling the molecule toward the liquid phase and again producing zero average dipole moment. Finally comes the ***po***/*z*_o_^5^ term. It produces no average dipole moment. The two *z*_o_^–5^ terms are interesting with their effect on the orientation of the population of water molecules lying with dipole moment parallel to the surface, as discussed below.

Equations 13 and 18 are the multipolar O­(*z*_o_^–6^) expansions of the image energy *u*; the largest neglected terms in the series are the ***qo***/*z*_o_^6^ and ***ph***/*z*_o_^6^ interactions (see Table 1). We see that, to produce an expression for the image potential that is O­(*z*_o_^–6^), one must include a molecular octupole moment in the expansion – because, although the ***oo*** term is *z*_o_^–7^ and can be neglected, the cross-interaction ***po*** is *z*_o_^–5^. Similarly, to produce an O­(*z*_o_^–4^) expansion of the image potential acting on a charged particle, just the ***pp***/*z*_o_^3^ is not sufficient – the mixed *e* ***q*** term is of the same order of magnitude, as will be discussed in the companion paper.

Since hydrogen bonding in water is of predominantly electrostatic nature, a multipole expansion up to octupole terms means that the hydrogen bonding is implicitly included in our electrostatic model. A point dipole–quadrupole-octupole gives reasonably accurate description of the hydrogen bonding.^32^ For example, multipoles higher than octupole contribute at most ± 15% to the hydrogen bonding energy of a given configuration of an ice lattice.^33^

### Polarization of the Dipole by Its Image

The image field polarizes the molecule; the image field gradient quadrupolarizes it and so forth. We neglect the molecular quadrupolarizability as it produces an effect of shorter range (see Table 1); for the same reason, we neglect the contributions of ***q*** and ***o*** to ***E***_im_ in eq 11 when calculating the field that polarizes the molecule. The dipole moment ***p*** = (*p*_*x*_,*p*_*y*_,*p*_*z*_) in the expression [Disp-formula eq6] is different from ***p***_0_ of a water molecule in vacuum due to the action of ***E***_im_:

$$p=p_{0}+\alpha_{p}E_{\mathrm{im}}$$ 20

where

$$E_{\mathrm{im}}\approx -k_{\mathrm{im}}^{L}\frac{p_{x}e_{x}+p_{y}e_{y}+2p_{z}e_{z}}{{\left|2z_{o}\right|}^{3}}$$ 21

from eqs 11 and 6. Equations 20 and 21 are vector equations for ***p*** and ***E***_im_; the solution for the dipole moment is

$$p_{x}=\frac{p_{0x}}{1+k_{\mathrm{im}}^{L}\alpha_{p}/{\left|2z_{o}\right|}^{3}},p_{y}=\frac{p_{0y}}{1+k_{\mathrm{im}}^{L}\alpha_{p}/{\left|2z_{o}\right|}^{3}},p_{z}=\frac{p_{0z}}{1+2k_{\mathrm{im}}^{L}\alpha_{p}/{\left|2z_{o}\right|}^{3}}$$ 22

Thus, the image field depolarizes the dipoles located in the more polar phase (water).

However, if the dipole is in the gaseous phase, the opposite is true – it is polarized by the image force; moreover, the effect is of larger magnitude (the image field is larger as ε^G^ ≪ ε^W^). From symmetry considerations:

$$p_{x}=\frac{p_{0x}}{1-k_{\mathrm{im}}^{G}\alpha_{p}/{\left|2z_{o}\right|}^{3}},p_{y}=\frac{p_{0y}}{1-k_{\mathrm{im}}^{G}\alpha_{p}/{\left|2z_{o}\right|}^{3}},p_{z}=\frac{p_{0z}}{1-2k_{\mathrm{im}}^{G}\alpha_{p}/{\left|2z_{o}\right|}^{3}}$$ 23

The free energy acquired by a polarizable dipole in the image field (created by the dipole ***p*** and proportional to its magnitude) is the sum of the electric energy – 1/2 ***p*** · ***E***_im_ of dipole ***p*** in the field ***E***_im_ plus the elastic energy + (***p*** – ***p***_0_)^2^/2α_*p*_ lost on the polarization of the dipole. Together with eq 20, this produces the expression (cf. refs 34 and 35):

$$u_{pp}=-\frac{1}{2}p\cdot E_{\mathrm{im}}+\frac{1}{2\alpha_{p}}{\left(p-p_{0}\right)}^{2}=-\frac{1}{2}p_{0}\cdot E_{\mathrm{im}}$$ 24

We substitute here ***E***_im_ from eq 21 and ***p*** from eq 22 to obtain

$$u_{pp}^{L}=\frac{k_{\mathrm{im}}^{L}}{2}\left(\frac{p_{0x}^{2}+p_{0y}^{2}}{1+k_{\mathrm{im}}^{L}\alpha_{p}/{\left|2z_{o}\right|}^{3}}+\frac{2p_{0z}^{2}}{1+2k_{\mathrm{im}}^{L}\alpha_{p}/{\left|2z_{o}\right|}^{3}}\right)\frac{1}{{\left|2z_{o}\right|}^{3}}$$ 25

We neglected the polarizability effect on the higher-order (***pq*** and ***po***) terms in *u*, i.e. they remain unchanged compared to the solid multipole case (eq 13). This is because the effect of α_*p*_ on the ∇ ***E***_im_ and ∇∇ ***E***_im_ terms in the energy produces high-order corrections (the most long-ranged one is ∝α_*p*_ ***pq***/*z*_o_^7^ from the cross ***pq*** term).

Therefore, the total energy of a polarizable molecule in an image field is

$$u^{L}=\frac{k_{\mathrm{im}}^{L}}{2}\left(\frac{U_{pp}^{L}}{{\left|2z_{o}\right|}^{3}}+\frac{U_{pq}}{{\left|2z_{o}\right|}^{4}}+\frac{U_{qq}+U_{po}}{{\left|2z_{o}\right|}^{5}}\right)$$ 26

where

$$U_{pp}^{L}=\frac{p_{0x}^{2}+p_{0y}^{2}}{1+k_{\mathrm{im}}^{L}\alpha_{p}/{\left|2z_{o}\right|}^{3}}+\frac{2p_{0z}^{2}}{1+2k_{\mathrm{im}}^{L}\alpha_{p}/{\left|2z_{o}\right|}^{3}}$$ 27

For the gas phase, we similarly find

$$u^{G}=\frac{k_{\mathrm{im}}^{G}}{2}\left(-\frac{U_{pp}^{G}}{{\left|2z_{o}\right|}^{3}}+\frac{U_{pq}}{{\left|2z_{o}\right|}^{4}}-\frac{U_{qq}+U_{po}}{{\left|2z_{o}\right|}^{5}}\right),\mathrm{where}$$

$$U_{pp}^{G}=\frac{p_{0x}^{2}+p_{0y}^{2}}{1-k_{\mathrm{im}}^{G}\alpha_{p}/{\left|2z_{o}\right|}^{3}}+\frac{2p_{0z}^{2}}{1-2k_{\mathrm{im}}^{G}\alpha_{p}/{\left|2z_{o}\right|}^{3}}$$ 28

The expansions (eqs 26 and 28) are still O­(*z*_o_^–6^), like eq 13 for solid multipoles. Even the first correction for the polarizability is *z*_o_^–6^ (the expansion of eq 27 in series produces α_*p*_ ***p*** ^2^/*z*_o_^6^ – a kind of Debye force). However, the molecular polarizability in eqs 27 and 28 is coupled with the largest ***pp*** term in *u* ^L^ and *u* ^G^ which produces a strongly nonlinear distribution, see below; the nonlinearity has a magnifying effect on the role of the polarizability.

### Multipole in a Cavity

The molecules are not point sources in continuous medium. They have finite size and carry a solvation shell. In first approximation, the solvation shell can be modeled as a spherical cavity^36–38^ of effective radius *R*_cav_. The cavity produces two important effects:^36^ *the reaction field* effect and the *cavity field* effect.

The reaction effect is due to the central molecule orientating its neighbors. The produced structure of the solvation shell results in a nonzero mean field inside the cavity – the reaction field^36^ ***E***_react_ – that polarizes appreciably all molecules in the liquid phase. ***E***_react_ is the reason why the force models for water molecules used in MD simulations of liquids are of larger dipole moment than water in vacuum, i.e. *p* > *p*_0_. Moreover, the dipole moment of the structure *molecule* + *polarized cavity* as a whole (the so-called *external dipole*,^36^ ***p***_ext_) is even larger – *p*_ext_ > *p* (see Supplement S7).

The cavity field effect is due to the refraction of ***E***_im_ by the cavity wall; it produces a stronger field inside the cavity (*E*_cav_ > *E*_im_). Moreover, the polarization of the cavity by ***E***_im_ also produces an external dipole moment, ***p***_ext,*E*_, which modifies ***E***_im_.

Similarly, a point quadrupole^37,38^ in the cavity produces a reaction field gradient ∇ ***E***_react_, and the cavity reflects the image field gradient ∇ ***E***_im_ to produce a larger cavity field gradient ∇ ***E***_cav_. The ensemble *central quadrupole* + *cavity* acquires an external quadrupole ***q***_ext_ + ***q***_ext,∇*E*_.

Both the reaction and the cavity field effects are diminished nearby the surface,^19^ for two reasons: first, the density of water is reduced, and second, the surface layer is significantly polarized, and the dielectric saturation there reduces the local response to external field (see below).

We will only provide an approximate analysis of the two effects, and we will only correct the largest terms in the multipole expansion of the image potential, ***pp*** and ***pq***. In Supplement S7, we show that, at large distance from the surface, eq 27 for the polarizable version of the ***pp*** interaction coefficient is modified to

$$U_{pp,\mathrm{cav}}^{L}=\frac{Y_{E}^{2}}{{\left(1-\alpha_{p}X_{p}\right)}^{2}}\left(\frac{p_{0,x}^{2}+p_{0,y}^{2}}{1+k_{\mathrm{im}}^{L}\alpha_{p,\mathrm{cav}}/{\left|2z_{o}\right|}^{3}}+\frac{2p_{0,z}^{2}}{1+2k_{\mathrm{im}}^{L}\alpha_{p,\mathrm{cav}}/{\left|2z_{o}\right|}^{3}}\right)$$ 29

and similarly for eq 28 in the gas. Here,^36–38^ we introduced the quantities:

$$\alpha_{p,\mathrm{cav}}=\frac{\alpha_{p}Y_{E}^{2}}{1-\alpha_{p}X_{p}}-\frac{4\pi }{3}R_{\mathrm{cav}}^{3}\left(\varepsilon -\varepsilon_{0}\right)Y_{E};Y_{E}=\frac{3f_{E}\varepsilon }{2\varepsilon +f_{p}\varepsilon_{0}};Y_{\nabla E}=\frac{5f_{\nabla E}\varepsilon }{3\varepsilon +2f_{q}\varepsilon_{0}};X_{p}=\frac{1}{2\pi \varepsilon_{0}R_{\mathrm{cav}}^{3}}\frac{\varepsilon -f_{p}\varepsilon_{0}}{2\varepsilon +f_{p}\varepsilon_{0}};X_{q}=\frac{9}{4\pi \varepsilon_{0}R_{\mathrm{cav}}^{5}}\frac{\varepsilon -f_{q}\varepsilon_{0}}{3\varepsilon +2f_{q}\varepsilon_{0}}$$ 30

*Y*_*E*_ are the Onsager cavity field coefficient (***E***_cav_ = *Y*_*E*_ ***E***_im_); *Y*_∇*E*_ is the cavity field gradient coefficient (∇ ***E***_cav_ = *Y*_∇*E*_∇ ***E***_im_); *X*_*p*_ is the reaction field coefficient (*E*_react_ = *X*_*p*_ ***p***); *X*_*q*_ is the reaction field gradient coefficient (∇*E*_react_ = *X*_*q*_ ***q***); α_*p*,cav_ is the polarizability of the assembly molecule + cavity. The quadrupolar factors *f*_*E*_, *f*_∇*E*_, *f*_*p*_, and *f*_*q*_ are related to the quadupolarizability of the medium and for water are close to 1 (i.e., eq 30 simplifies to the Onsager model^36^); the formulas for them are given by eqs 104 and 113 in Supplement S7.

The cavity coefficients and α_*p*,cav_ in eq 29 are functions of the distance from the surface. They can be calculated from the quadrupolar version^37,38^ of the Onsager cavity dielectric equation of state.^36^ This is done in Supplement S8. The calculation requires a certain model for how *R*_cav_ depends on the local density of water. Two such models are tested in Supplement S8, as limiting cases: one where *R*_cav_ is almost constant and another where it follows the Onsager formula *R*_cav_ ∼ *C* ^–1/3^ (reaching the experimental value in the bulk water, 1.39 Å). Deep inside the liquid phase, the coefficient in front of the large bracket of eq 29 is close to 3, i.e. the image force is enhanced significantly. Close to the surface, the effect is reduced due to the reduced density of the interphase and the dielectric saturation. The effective polarizability α_*p*,cav_ is large and negative for water.

The ***pq*** interaction coefficient (eq 15) is also generalized to a multipole in a cavity in Supplement S7:

$$U_{pq,\mathrm{cav}}=-\frac{Y_{E}Y_{\nabla E}}{\left(1-\alpha_{p}X_{p}\right)\left(1-\alpha_{q}X_{q}\right)}\left[6\left(p_{0x}q_{xz}+p_{0y}q_{yz}\right)+9p_{0z}q_{zz}\right]$$ 31

The coefficient in front of the square bracket is close to 3 × 2 = 6 in the liquid phase and smoothly drops to 1 in the gas, see Figure 2.

**Figure 2:**
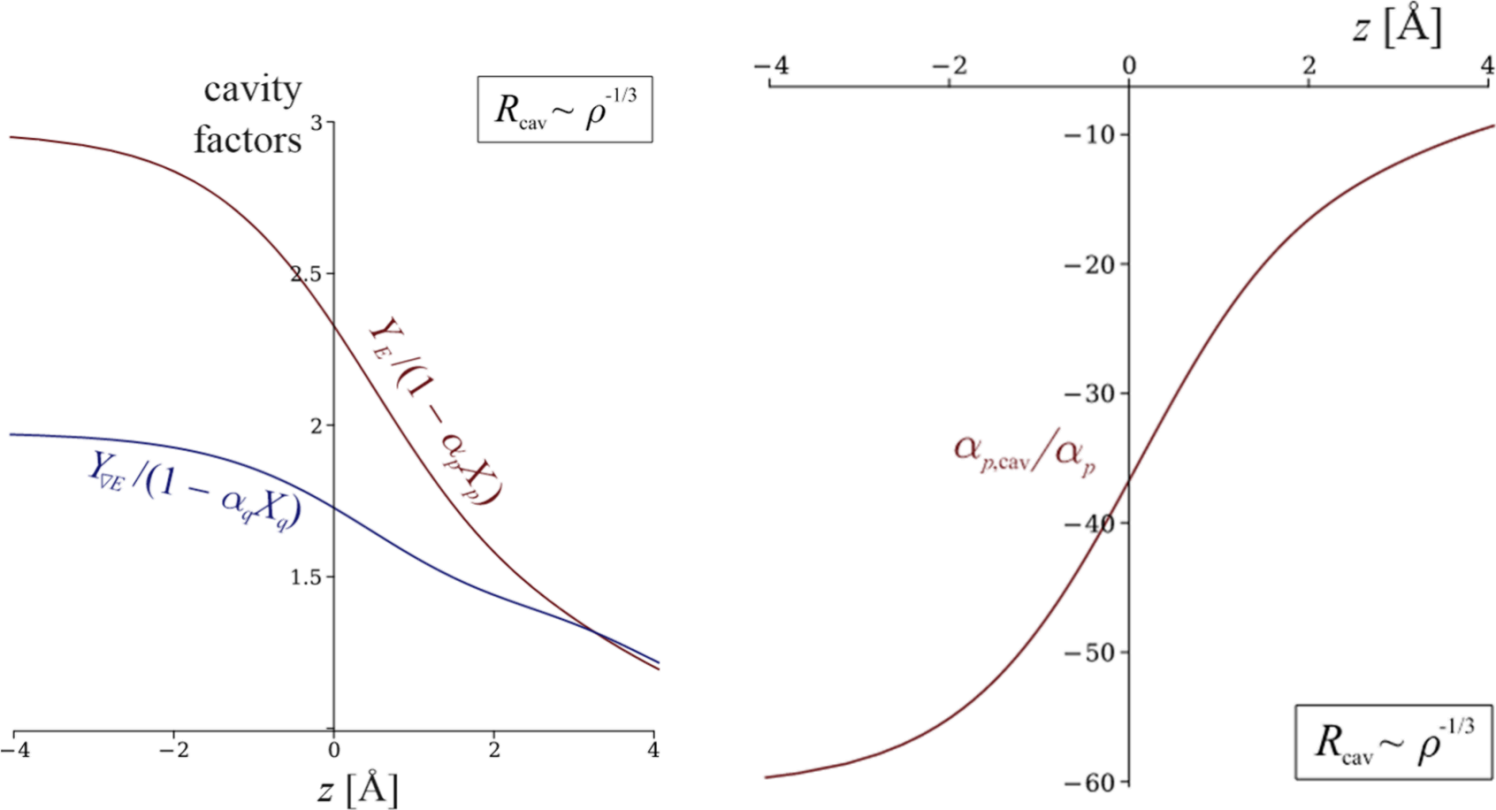
Cavity factors as a function of the distance to the dielectric surface.

One corollary to these results is that the water dipole moment varies with the location – ***p*** is different in the surface layer, the bulk liquid and the gas phase. By contrast, all standard classical MD force fields for liquid water use models with fixed dipole moment larger than ***p***_0_ by a factor of ∼ 1.5, as a way to correct for the reaction field in the bulk. This may mean that nonpolarizable MD models overestimate the image forces in the surface layer.

### Probability Distribution

The Boltzmann distribution with respect to the Euler angles reads:

$$\rho_{\varphi \psi \theta }=c_{n}e^{-u/kT}$$ 32

where *u* is the image energy, (eq 26) in the liquid or (eq 28) in the gas. The coefficient *c*_n_ is determined from the normalization condition:

$$c_{n}=1/\int_{0}^{\pi }\int_{0}^{2\pi }\int_{0}^{2\pi }e^{-u/kT}\sin\;\theta d\varphi d\psi d\theta$$ 33

The energy *u* is a function of the Euler angles through the components of ***p***_0_, ***q*** and ***o*** in eqs 14–17and 27; the dependence is set by the formulas:

$$p_{0i}=E_{ij}^{\varphi \psi \theta }p_{n,j}$$ 34

$$q_{ij}=E_{ik}^{\varphi \psi \theta }E_{jl}^{\varphi \psi \theta }q_{n,kl}$$ 35

$$o_{fgh}=E_{fi}^{\varphi \psi \theta }E_{gj}^{\varphi \psi \theta }E_{hk}^{\varphi \psi \theta }o_{n,ijk}$$ 36

Here, n subscript indicates a chosen reference orientation of the water molecule – e.g., the one where the dipole is in direction + **e**_*z*_. Another condition can be imposed on the reference orientation that simplifies ***q***_n_ by rotation around the axis of ***p***_n_. ***E*** ^φψθ^ is the Euler tensor:

$$E^{\varphi \psi \theta }=\left(\begin{array}{ccc} \begin{array}{c} \cos\;\varphi \cos\;\psi -\\ \sin\;\varphi \sin\;\psi \cos\;\theta \end{array} & \begin{array}{c} \sin\;\varphi \cos\;\psi +\\ \cos\;\varphi \sin\;\psi \cos\;\theta \end{array} & \sin\;\psi \sin\;\theta \\ \begin{array}{c} -\cos\;\varphi \sin\;\psi -\\ \sin\;\varphi \cos\;\psi \cos\;\theta \end{array} & \begin{array}{c} -\sin\;\varphi \sin\;\psi +\\ \cos\;\varphi \cos\;\psi \cos\;\theta \end{array} & \begin{array}{c} \cos\;\psi \sin\;\theta \end{array}\\ \begin{array}{c} \sin\;\varphi \sin\;\theta \end{array} & \begin{array}{c} -\cos\;\varphi \sin\;\theta \end{array} & \begin{array}{c} \cos\;\theta \end{array} \end{array}\right)$$ 37

Here, the angle θ is azimuth between ***p***_0_ and **e**_*z*_; ψ is polar angle for the rotation around the axis *z*; φ is polar for rotation around the axis of ***p***_0_.

For molecules for which ***q***_n_ and ***o***_n_ can be diagonalized by rotation around the axis of ***p***_n_, the distribution becomes independent of ψ. Water (point group C_2v_) is such a molecule; therefore, the distribution ρ_φψθ_ of water is a function of θ and φ only (i.e., it is ρ_φθ_). This results in relatively simple expressions for the dependence of the coefficients (eqs 17 and 27) on θ and φ for water; these are given in Supplement S5 for reference.

### Choice of Origin and Values for Water

One problem that has received little attention in the literature is that the quadrupole and octupole moments of a polar molecule depend on the origin. This is more than just a theoretical conundrum: the value of ***q*** that corresponds to origin in the center of mass (***q***_*m*_) and the value that corresponds to origin in the center of the oxygen atom (***q***_n_) are so different that they formally correspond to surface potentials of different signs. Therefore, the origin has to be chosen carefully.

Most standard force models of water set the origin of the (centrosymmetric) Van der Waals forces in the oxygen nucleus.^21^ The origin of the steric repulsion term could be placed, e.g., in the center of mass instead. However, to achieve an equivalent description with another origin, a dependence of the steric force on the rotation angles would be required – that is, the steric force has a torque with respect to the center of mass, and this torque orientates the bulky oxygen atom in the direction of the less dense phase.

In the following sections, upon integration of the local mean dipole moment, we treat the electrostatic forces as a perturbation over a model tanh distribution of the molecules around the surface. If the origin is at the oxygen nucleus, this initial unperturbed distribution depends solely on *z*, and we can assume that the orientation is due to the electrostatic image force only (noting that orientation due to hydrogen bonding is equivalent to higher-order multipole electrostatics^32,33^ ).

However, if the origin is chosen in the center of mass, the assumed unperturbed distribution around the surface is already complex even before the perturbation by the image forces – because it is a function of both the distance from the surface and the orientation of water with respect to the surface.

Therefore, we will set the origin of the point multipole in the oxygen nucleus. This was the choice also by Stillinger and Ben Naim.^19^ A better description would be achieved if the origin is chosen at the point where the sum of torques from the steric force and the neglected electrostatic O­(*z*_o_^–6^) forces, ***ph*** and ***qo***, has an average of zero – but we leave this for future research.

Let the symbols ***p***_n_, ***q***_n_, and ***o***_n_ refer to water molecule orientated with its dipole in direction + **e**_*z*_ (oxygen down), with all nuclei in the *xz* plane (here ‘n’ stands for ‘normal’ orientation). In this orientation, due to the symmetry, all cross components of ***q*** are zero (*q*_n,*xy*_ = *q*_n,*xz*_ = *q*_n,*yz*_ = 0). Therefore:

$$p_{n}=\left(0,0,p_{0}\right),q_{n}=\left(\begin{array}{ccc} q_{xx} & 0 & 0\\ & -q_{xx}-q_{zz} & 0\\ & & q_{zz} \end{array}\right)$$ 38

Furthermore, all but seven components of ***o*** are zero, and only two of them are independent:

$$o_{n}=\left(\begin{array}{ccc} 0 & 0 & o_{xxz}\\ & 0 & 0\\ & & 0 \end{array}\left|\begin{array}{ccc} 0 & 0 & 0\\ & 0 & o_{yyz}\\ & & 0 \end{array}\right|\begin{array}{ccc} o_{xxz} & 0 & 0\\ & o_{yyz} & 0\\ & & -o_{yyz}-o_{xxz} \end{array}\right)$$ 39

We use the multipole values calculated by Batista et al.^39^ These authors used origin at the center of mass (***p***_*m*_, ***q***_*m*_, and ***o***_*m*_) and orientation of water in the plane *xz* but with dipole moment in direction **-e**_*z*_ (oxygen up). The distance between the two origins–the oxygen nucleus and the center of mass–is *d* = 0.06586 Å for water.^39^ The rules to switch between values for origin at the mass center and origin at O are summarized in Supplement S4; Table 2 lists the formal values for two origins and two orientations.

**Table 2:** Molecular Parameters of Water^a^

**indices**	***p***_*m*_, ***q***_*m*_, ***o***_*m*_ ^b^	***p***_O_, ***q***_O_, ***o***_O_ ^c^	***p***_n_, ***q***_n_, ***o***_n_ ^d^
origin	center of mass	oxygen nucleus	oxygen nucleus
orientation	atoms in *xz* plane; oxygen in direction + **e**_*z*_	atoms in *xz* plane; oxygen in direction + **e**_*z*_	atoms in *xz* plane; oxygen in direction -**e**_*z*_
*p*_*z*_ [Cm] × 10^30^	–6.204	–6.204	+6.204
*q*_*xx*_ [Cm^2^] × 10^40^	+5.812	+5.539	+5.539
*q*_*yy*_ [Cm^2^] × 10^40^	–5.517	–5.789	–5.789
*q*_*zz*_ = −*q*_*xx*_ – *q*_*yy*_ [Cm^2^] × 10^40^	–0.295	+0.250	+0.250
*o*_*xxz*_ [Cm^3^] × 10^50^	–3.107	–3.476	+3.476
*o*_*yyz*_ [Cm^3^] × 10^50^	+1.297	+1.675	–1.675
*o* *_zzz_* = −*o* *_xxz_* – *o*_*yyz*_ [Cm^3^] × 10^50^	+1.810	+1.801	–1.810
α_*p*_/4πε_0_ [Å^3^]	1.470	1.470	1.470

a Calculated ab initio by Batista et al.^39^–second-order Møller–Plesset level calculations with the aug-cc-pVQZ basis.

b The values of Batista et al. are converted to SI units, and their values of ***q***_*m*_ and ***o***_*m*_ are multiplied by 2/3 and 2/5, respectively, due to the different coefficients in the definitions.

c Converted to origin in the oxygen nucleus, see Supplement S4; oxygen still points up.

d Converted to the normal orientation (dipole points up, oxygen points down).

Compared to *q*_*zz*_, the ‘magnitude’ ***q***: ***q*** of the quadrupole (which controls the macroscopic quadrupolarizability^37,38^ ) and the difference *q*_*xx*_ − *q*_*yy*_ (the spherical component of the quadrupole that controls the orientation of water when its dipole lies parallel to the surface, see Supplement S5) are only very weakly dependent on the choice of origin.

### Orientation of a Water Molecule Due to the Image forces

The orientation predicted by eqs 32–36, with energy (eq 13) in the liquid and (eq 18) in the gas phase, is illustrated in Figure 3 for the point multipole model and for two values of *z*_o_, one below and another above the dielectric surface. The values were chosen so that the magnitudes of *U*_*pp*_/*z*_o_^3^ and *U*_*qq*_/*z*_o_^5^ are comparable and that significant orientation in *z* direction appears. At longer distances, the dipole–dipole term dominates, causing preferential orientation of the dipole parallel to the surface in the liquid phase, or normal to the surface in the gas phase – but with the directions + **e**_*z*_ and -**e**_*z*_ being equally probable. The image force is not the only one acting at longer distance: the formation of a DDL produces an electrostatic force orientating the molecules normally in the diffuse dipole layer, but with the oxygen toward the liquid phase (Supplement S10). At shorter distances, the neglected higher-order multipoles become significant and the induction effects in eqs 27 and 28 approach unrealistic magnitude (see below).

**Figure 3:**
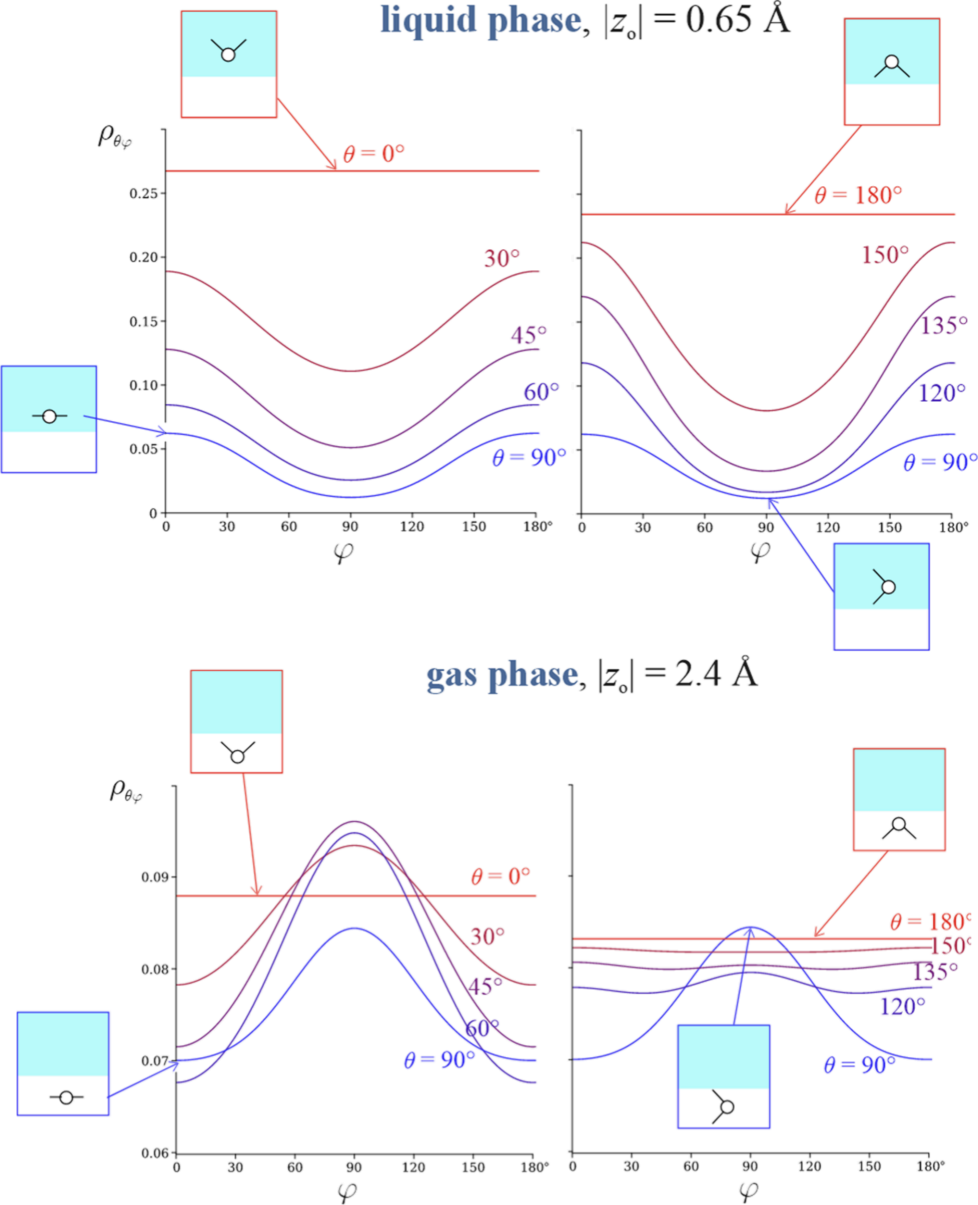
Up: probability distribution for orientation of a water molecule in the liquid phase, 0.65 Å away from the dielectric surface. Down: same for water in the gas phase, 2.4 Å away from the dielectric surface. Point multipole model, *u* = *u*_0_; parameters from Table 2.

For molecules with the symmetry of water, it holds true that *u*(φ + π) = *u*(φ); therefore, we plot only the range φ = 0–180° (the distribution in φ = 180–360° is identical). For both phases, the probability of finding the molecule with the oxygen toward the gas phase (corresponding to θ = 0) is higher than the one where O points toward the liquid (θ = 180°). This is due to the cross-interaction ***pq***/*z*_o_^4^ between dipole and quadrupole.

There is an ongoing discussion in the literature^31^ about whether the hydrogens lie parallel to the surface or normal to the surface (i.e., the distribution with respect to φ for θ = 90°), based mostly on MD simulations and sum frequency generation data. It is interesting to see what follows from pure electrostatics – especially since the most common MD models of water have higher multipole moments that do not match those of real water. Value of θ = 90° corresponds to ***p*** lying flat on the surface; φ then corresponds to rotation around the ***p***-axis, with φ = 0 meaning the whole water molecule lies flat on the surface (hydrogens in the *xy* plane) and φ = 90° – water normal to the surface (molecule in the *xz* plane, with the axis H–H connecting the two hydrogens being normal to the surface – see the inset schematics in Figure 3). For the symmetry of water, only the *U*_*qq*_/*z*_o_^5^ and *U*_*po*_/*z*_o_^5^ terms contribute to the distribution with respect to φ (because, at θ = 90°, *U*_*pq*_ = 0 and *U*_*pp*_ is constant, see Supplement S5). The dependence of *u* on φ in this case is controlled by the sum:

$$U_{qq}+U_{po}=\mathrm{const}+\frac{15}{2}\left[\frac{3}{4}(q_{xx}-q_{yy})q_{zz}-p_{0}(o_{xxz}-o_{yyz})\right]\cos\;2\varphi +\frac{9}{16}(q_{xx}-q_{yy}{)}^{2}\cos^{2}\;2\varphi$$ 40

For water, *U*_*qq*_ + *U*_*po*_ = const + (−224·cos2φ + 72·cos^2^2φ) × 10^–80^ C^2^m^4^, so the cos2φ term dominates. This results in weak preferential orientation to φ = 0° in the liquid phase (all atoms parallel to the surface) and strong preferential orientation to φ = 90° in the gas phase (‘dangling bonds’ configuration with H–H normal to the surface)–see Figure 3, blue line and insets. This behavior is mostly due to the large *U*_*po*_ term in eqs 13 and 18. The (*q*_*xx*_ – *q*_*yy*_)^2^cos^2^2θ term can, in principle, cause preferential orientation at θ = 90°, φ = 45° (dipole parallel to the surface, the H–H axis under 45°) – which indeed has been noticed in MD simulations.^31^ Based on eq 40, we can say that, for a MD simulation to produce a qualitatively correct orientation of water, it is essential that the water model has not only the accurate *q*_n,*zz*_ and *q*_n,*xx*_ values, but also accurate *o*_n,*xxz*_ – *o*_n,*yyz*_.

For the calculation of the average orientation of water in the surface layer, we will use the nonlinear distribution (eq 32). However, it is useful to compare those with what follows from the linearized version of the Boltzmann distribution; moreover, the linearized distribution is sufficient for a class of interfaces where ε^L^ and ε^G^ are not too different (less polar fluids, fluids near their critical point, certain solid|liquid interfaces etc.). The linear form of eq 32 is

$$\rho_{\varphi \psi \theta }=c_{n}e^{-\mathrm{u̅}/kT}e^{-\left(u-\mathrm{u̅}\right)/kT}\approx \frac{1}{8\pi^{2}}\left(1-\frac{u\left(\varphi ,\psi ,\theta \right)-\mathrm{u̅}}{kT}\right)$$ 41

where *u̅* is the image potential averaged over all orientations. For a solid dipole without cavity in the liquid phase:

$${\mathrm{u̅}}_{0}^{L}=\frac{2}{3}k_{\mathrm{im}}^{L}\frac{p_{0}^{2}}{{\left|2z_{o}\right|}^{3}}+\frac{12}{5}k_{\mathrm{im}}^{L}\frac{q:q}{{\left|2z_{o}\right|}^{5}}$$ 42

This average image potential contributes to the adsorption energy of polar-quadrupolar molecules to any interface.

The linearized distribution allows the average characteristics of a molecule near a surface to be obtained in a closed form. For nonpolarizable molecules, using eqs 41, 13, and 34–36, we obtain

$${\mathrm{p̅}}_{z}^{L}=\int_{0}^{\pi }\int_{0}^{2\pi }\int_{0}^{2\pi }p_{0z}\rho_{\varphi \psi \theta }\sin\;\theta d\varphi d\psi d\theta =k_{\mathrm{im}}^{L}\frac{3q_{n,zz}p_{0}^{2}}{40kT}\frac{1}{z_{o}^{4}}$$ 43

Here, *p*_0*z*_ is given by eq 34, ρ_ϕψθ_ – by eq 41 with energy eq[Disp-formula eq13] without α_*p*_. This result holds for any molecule, not just those having the symmetry of water.

For water, *q*_n,*zz*_ > 0 (when the origin is at the oxygen nucleus, see Table 2); therefore, *p̅*_*z*_^L^ > 0. The coordinate system has the water phase at *z* > 0, so eq 43 predicts the negative pole of water points, on the average, toward the gas phase – confirming the conclusion of Stillinger and Ben Naim.^19^

Using the symmetry of the problem, for a water molecule in the gas phase we can write

$${\mathrm{p̅}}_{z}^{G}=k_{\mathrm{im}}^{G}\frac{3q_{n,zz}p_{0}^{2}}{40kT}\frac{1}{z_{o}^{4}}$$ 44

The orientation is again with the oxygen toward the gas phase, but in the gas the image field is much stronger (ε^G^ ≪ ε^L^ and *k*_im_^G^ ≫ *k*_im_^L^), producing stronger orientation. As a result, the average location of the adsorbed dipole layer is above the dielectric surface (see below).

The sign of the surface potential of a simple liquid is determined by the sign of *q*_n,*zz*_ (i.e., the sign of ***p*** · ***q*** · ***p***). Let us reiterate that the choice of origin matters; the correct origin is the one that corresponds to zero steric torque. Inconsistent choice of origin (e.g., *q*_*m*,*zz*_ = −0.259 × 10^–40^ C·m^2^ instead of *q*_n,*zz*_ = +0.250 × 10^–40^ C·m^2^, see Table 2) can produce potential of the wrong sign for water.

For a polarizable molecule, eq. 43 and ([Disp-formula eq44]) are generalized to (see Supplement S6):

$${\mathrm{p̅}}_{z}^{L}=\frac{k_{\mathrm{im}}^{L}}{1+2k_{\mathrm{im}}^{L}\alpha_{p}/\left|2z_{o}^{3}\right|}\frac{3q_{n,zz}p_{0}^{2}}{40kT}\frac{1}{z_{o}^{4}}\;\mathrm{and}\;{\mathrm{p̅}}_{z}^{G}=\frac{k_{\mathrm{im}}^{G}}{1-2k_{\mathrm{im}}^{G}\alpha_{p}/\left|2z_{o}^{3}\right|}\frac{3q_{n,zz}p_{0}^{2}}{40kT}\frac{1}{z_{o}^{4}}$$ 45

For polarizable molecule in a dielectric cavity, this is further modified to

$${\mathrm{p̅}}_{z}^{L}=\frac{Y_{E}}{\left(1-\alpha_{p}X_{p}\right)}\frac{Y_{\nabla E}}{\left(1-\alpha_{q}X_{q}\right)}\frac{k_{\mathrm{im}}^{L}}{1+2k_{\mathrm{im}}^{L}\alpha_{p,\mathrm{cav}}/{\left|2z_{o}\right|}^{3}}\times \frac{3q_{n,zz}p_{0}^{2}}{40kT}\frac{1}{z_{o}^{4}}\;\mathrm{and}\;{\mathrm{p̅}}_{z}^{G}=\frac{Y_{E}}{\left(1-\alpha_{p}X_{p}\right)}\frac{Y_{\nabla E}}{\left(1-\alpha_{q}X_{q}\right)}\frac{k_{\mathrm{im}}^{G}}{1-2k_{\mathrm{im}}^{G}\alpha_{p,\mathrm{cav}}/{\left|2z_{o}\right|}^{3}}\times \frac{3q_{n,zz}p_{0}^{2}}{40kT}\frac{1}{z_{o}^{4}}$$ 46

The linearized-distribution formulas [Disp-formula eq43]–[Disp-formula eq45] for the average dipole moment are compared with the numerically computed ones from the nonlinear distribution eq 32 in Figure 4.

**Figure 4:**
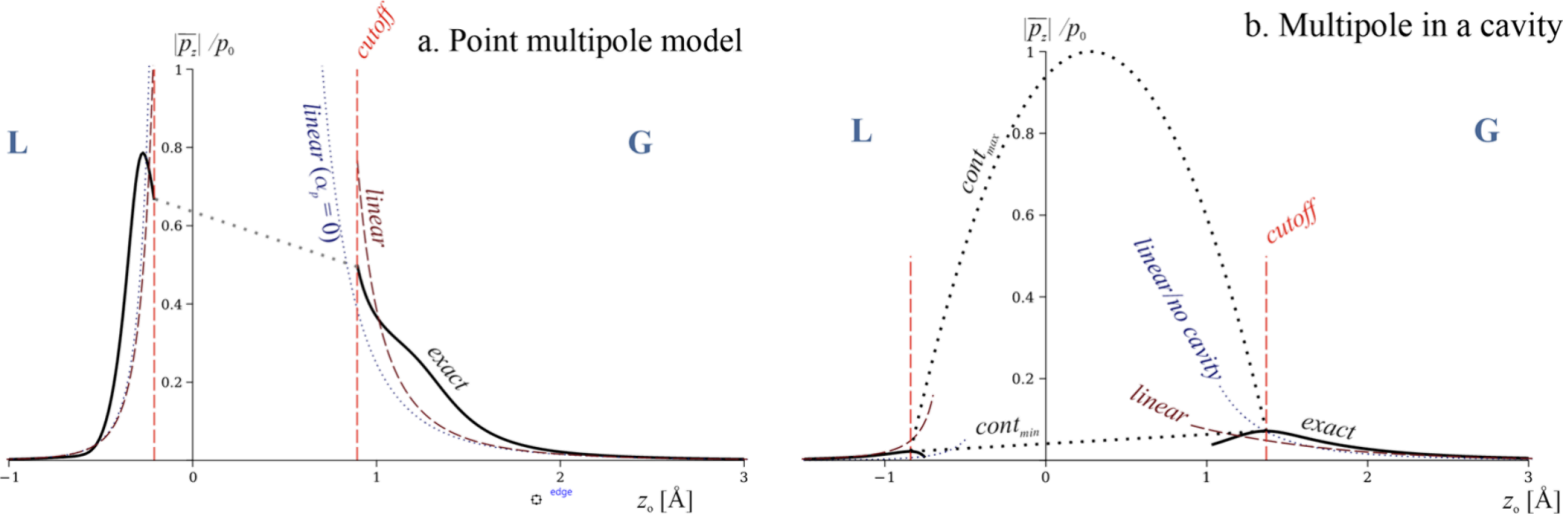
Average dipole moment as a function of the distance from the surface of water (oxygen points toward the gas phase). These are the unperturbed distributions, without the DDL response. (a) Point multipole model. Compared are the dipole calculated from the exact nonlinear distribution (eq 41) and the one from the linear formulas [Disp-formula eq43]–[Disp-formula eq45] (with and without molecular polarizability). Cutoff parameter ξ_cutoff_ = 1/2. (b) Multipole in a cavity; eqs 29–31 are used in the exact nonlinear distribution; compared to the linear eq 46.

Let us find also the average square of the normal and the tangential components of ***p*** for the linearized distribution:

$${\left(\frac{\overset{®}{p_{0z}^{2}\;}}{p_{0}^{2}}\right)}_{0}^{L}=\frac{1}{3}-\frac{8k_{\mathrm{im}}^{L}}{5kT}\left[\frac{p_{0}^{2}}{36{\left|2z_{o}\right|}^{3}}+\frac{3\left(q_{n,xz}^{2}+q_{n,yz}^{2}+q_{n,zz}^{2}\right)-q:q+5p_{0}o_{n,zzz}}{7{\left|2z_{o}\right|}^{5}}\right];{\left(\frac{\overset{®}{p_{0x}^{2}+p_{0y}^{2}\;}}{p_{0}^{2}}\right)}_{0}^{L}=\frac{2}{3}+\frac{8k_{\mathrm{im}}^{L}}{5kT}\left[\frac{p_{0}^{2}}{36{\left|2z_{o}\right|}^{3}}+\frac{3\left(q_{n,xz}^{2}+q_{n,yz}^{2}+q_{n,zz}^{2}\right)-q:q+5p_{0}o_{n,zzz}}{7{\left|2z_{o}\right|}^{5}}\right]$$ 47

The values 1/3 and 2/3 correspond to random distribution. For a molecule in the polar phase, the most long-ranged perturbation is proportional to *p*_o_^2^/*z*_o_^3^ and leads to preferential orientation of ***p*** in the plane *xy*. The correction from the quadrupole can be in either direction: large *q*_n,*xz*_, *q*_n,*yz*_ and *q*_n,*zz*_ will favor flat orientation of ***p***, while large *q*_n,*xx*_, *q*_n,*yy*_ and *q*_n,*xy*_ favor normal (but normal up and normal down with equal probability – therefore, not contributing to *p̅*_*z*_). Finally, the *p*_0_ *o*_n*,zzz*_/*z*_o_^5^ term leads to normal orientation if *o*_n,*zzz*_ < 0 and to tangential otherwise. For water, both the *qq* and *po* terms are negative, see Table 2, so water is predicted to have a tendency for normal orientation at very small values of *z*_o_ – the terms in the square bracket in eq 47 cancel at *z*_o_ = 2.0 Å, i.e. normal orientation will prevail at *z*_o_ < 2 Å. This is an overprediction: in reality, the *pp* contribution is larger due to the cavity effects.

In contrast, for a molecule in the gas phase, the *p*_o_^2^/*z*_o_^3^ term leads to preferential orientation in normal direction (for any polar molecule), and the *qq* and *po* terms – in tangential (for water). This is because the sign in front of the square bracket changes compared to the liquid-phase (eq 47):

$${\left(\frac{\overset{®}{p_{0z}^{2}\;}}{p_{0}^{2}}\right)}_{0}^{G}=\frac{1}{3}+\frac{8k_{\mathrm{im}}^{G}}{5kT}\left[\frac{p_{0}^{2}}{36{\left|2z_{o}\right|}^{3}}+\frac{3\left(q_{n,xz}^{2}+q_{n,yz}^{2}+q_{n,zz}^{2}\right)-q:q+5p_{0}o_{n,zzz}}{7{\left|2z_{o}\right|}^{5}}\right];{\left(\frac{\overset{®}{p_{0x}^{2}+p_{0y}^{2}\;}}{p_{0}^{2}}\right)}_{0}^{G}=\frac{2}{3}-\frac{8k_{\mathrm{im}}^{G}}{5kT}\left[\frac{p_{0}^{2}}{36{\left|2z_{o}\right|}^{3}}+\frac{3\left(q_{n,xz}^{2}+q_{n,yz}^{2}+q_{n,zz}^{2}\right)-q:q+5p_{0}o_{n,zzz}}{7{\left|2z_{o}\right|}^{5}}\right]$$ 48

The zero subscript indicates neglected molecular polarizability.

The above formulas can be generalized to polarizable molecules – there, the *pp* terms in the square brackets of eqs 47 and 48 are modified to (see Supplement S6 for a derivation):

$$\frac{p_{0}^{2}}{36\left(1+2k_{\mathrm{im}}^{L}\alpha_{p}/{\left|2z_{o}\right|}^{3}\right)\left(1+k_{\mathrm{im}}^{L}\alpha_{p}/{\left|2z_{o}\right|}^{3}\right){\left|2z_{o}\right|}^{3}}\;\mathrm{and}\;\frac{p_{0}^{2}}{36\left(1-2k_{\mathrm{im}}^{G}\alpha_{p}/{\left|2z_{o}\right|}^{3}\right)\left(1-k_{\mathrm{im}}^{G}\alpha_{p}/{\left|2z_{o}\right|}^{3}\right){\left|2z_{o}\right|}^{3}}$$ 49

The calculated the average *p*_0*z*_^2^/*p*_0_^2^ and (*p*_0*x*_^2^ *+p*_0*y*_^2^)/*p*_0_^2^ are shown in Figure 5; these quantities characterize the orientation of the molecules. The respective mean *p*_*z*_^2^/*p*_0_^2^ and (*p*_*x*_^2^ *+ p*_*y*_^2^)/*p*_0_^2^ are shown in Supplement S6; *p*_0*z*_^2^/*p*_0_^2^ and *p*_*z*_^2^/*p*_0_^2^ differ by a polarization-depolarization factor – see eqs 101 and 102 in Supplement S6.

**Figure 5:**
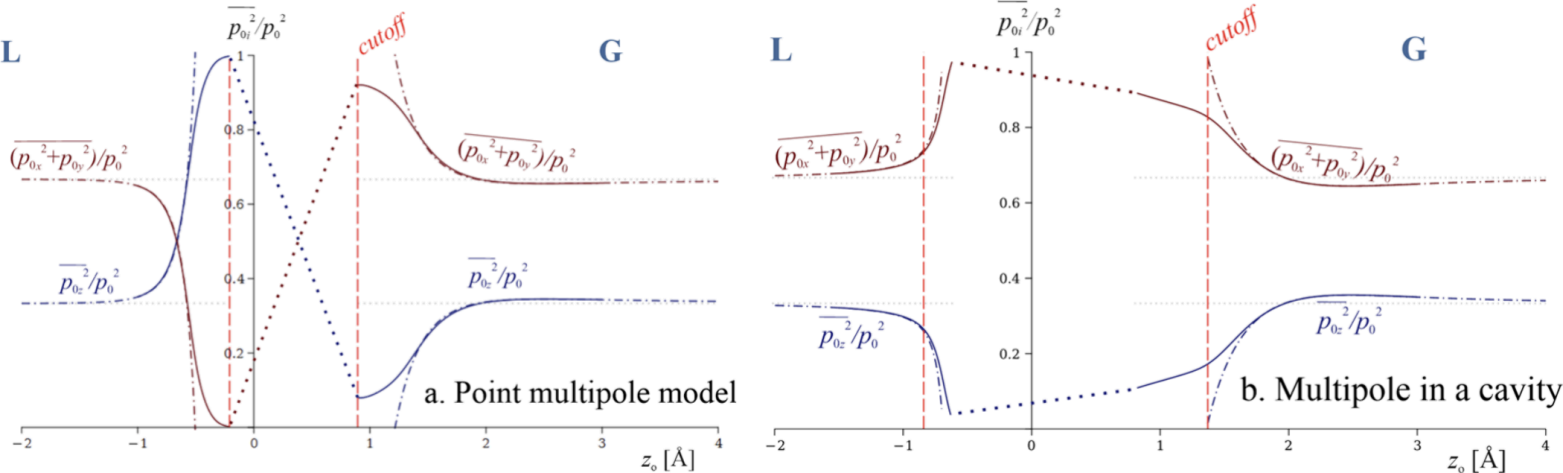
Mean square values of the normal and the tangential dipole moment of a water molecule near the dielectric surface. Solid lines: based on the nonlinear distribution (eq 41); dash-dot: linearized formulas [Disp-formula eq48]–[Disp-formula eq50]. (a) Point multipole model with cutoff parameter ξ_cutoff_ = 1/2. (b) Multipole in a cavity with *R*_cav_ ∼ ρ^–1/3^.

The polarization reduces the magnitude of the *pp* term for a molecule in the water but significantly increases it when the molecule is in the gas phase. This results in a peculiar nonlinear magnification effect on both the ***pp***/*z*_o_^3^ and the α_*p*_ ***pp***/*z*_o_^6^ terms in the multipole expansion that makes the role of α_*p*_ in the surface potential more important than the order of the effect in Table 1 would suggest.

The *pp* terms for a polarizable dipole in a cavity are further modified with the cavity factors and α_*p*,cav_ (see Supplement S7):

$$\frac{\frac{Y_{E}^{2}}{{\left(1-\alpha_{p}X_{p}\right)}^{2}}p_{0}^{2}}{36\left(1+2k_{\mathrm{im}}^{L}\alpha_{p,\mathrm{cav}}/{\left|2z_{o}\right|}^{3}\right)\left(1+k_{\mathrm{im}}^{L}\alpha_{p,\mathrm{cav}}/{\left|2z_{o}\right|}^{3}\right){\left|2z_{o}\right|}^{3}},\;\frac{\frac{Y_{E}^{2}}{{\left(1-\alpha_{p}X_{p}\right)}^{2}}p_{0}^{2}}{36\left(1-2k_{\mathrm{im}}^{G}\alpha_{p,\mathrm{cav}}/{\left|2z_{o}\right|}^{3}\right)\left(1-k_{\mathrm{im}}^{G}\alpha_{p,\mathrm{cav}}/{\left|2z_{o}\right|}^{3}\right){\left|2z_{o}\right|}^{3}}$$ 50

In the liquid phase, the effect from *Y*_*E*_, *X*_*p*_ and α_*p*,cav_ is very strong and leads to a much greater tangential orientation of the dipoles compared to the point multipole formula [Disp-formula eq47]. However, this effect is again overestimated; in reality, the higher order terms will also be of increased magnitude due to cavity effects. However, a more accurate model is pointless for our aims, because the molecules below the surface layer contribute little to the adsorbed dipole, see below.

In contrast, for the gas phase, the effects from the cavity factors *Y*_*E*_ and *X*_*p*_ and α_*p*,cav_ are acting in opposite direction (due to the minus in front of *k*_im_) and compensate each other to a significant extent.

The complex structure of the adsorbed layer revealed by Figures 4 and[Fig fig5] is further modified by the response of this layer to the electric field created by the spontaneously orientated dipoles – i.e. by the DDL. The unperturbed dipole *P*_0_^S^ of the adsorbed layer is reduced by the surface electric field ***E*** ^S^ of the DDL. ***E*** ^S^ is essentially a macroscopic reaction field caused by the nonvanishing normal dipole density. This field tends to depolarize the adsorbed layer of dipoles.^1^ Moreover, the adsorbed layer overlaps with a diffuse dipole layer which carries dipoles orientated in the opposite direction (especially below the dielectric surface, up to a distance of 2–3*L*_*Q*_). The DDL field decays exponentially, ***E*** = ***E*** ^S^exp­(−|*z*|/*L*_*Q*_), and produces a layer of molecules orientated with the oxygen toward the liquid phase (see below). The dipole moment carried by the diffuse layer nearly cancels the one of the adsorbed layer.^1^ Another effect is the smearing of the whole structure by thermal capillary waves.

The orientation of the molecules at the surface reduces the polarizability of the surface layer. To investigate the effect, we consider the effective polarizability α_mol_ per molecule (the quantity that appears in the Debye and Onsager models for the dielectric permittivity^36^). For it, the following approximate expressions hold:

$$\alpha_{\mathrm{mol},t}=\alpha_{p}+\frac{\overset{®}{p_{0x}^{2}+p_{0y}^{2}\;}}{2kT};\alpha_{\mathrm{mol},z}=\alpha_{p}+\frac{\overset{®}{p_{0z}^{2}\;}-{\mathrm{p̅}}_{0z}^{2}}{kT}$$ 51

The averages are taken in the absence of external field but in the presence of the image potential. These formulas are derived in Supplement S9 for a more general case that includes depolarization – see eq (128)&(129). The results can be compared to the Debye formula for the bulk polarizability per molecule: α_mol_ = α_*p*_ + *p*_0_^2^/3*kT*, where *p*_0_^2^/3 is the average value of *p*_0*i*_^2^ in the bulk phase.

As seen from eq 51 and Figure 5, a large fraction of the molecules at the surface contributes little to the tangential polarizability compared to those in the bulk – due to their preferential normal orientation by the strong image field, they respond less to tangential field. On the opposite, another fraction – right above the dielectric surface – contributes more to the tangential polarizability. This alteration of the polarizability results in a shift of the dielectric surface compared to the equimolecular surface: these two surfaces would match for nonpolar liquids but do not coincide for polar ones.

The situation is different for the normal polarizability α_mol,*z*_, due to the pyroelectric nature of the interphase – according to eq 51, the normal polarizability per molecule is reduced if there is significant orientation in one direction; for fully orientated molecule in one direction, α_mol,*z*_ vanishes.

### Macroscopic Characteristics of the Polarized Surface

The derived expressions [Disp-formula eq26]–[Disp-formula eq28] for the energies *u* ^L^ and *u* ^G^ of a point multipole, and eqs 29–31 for multipole in a cavity, are not valid at small values of *z*_o_, for at least three reasons.

The first one is the structure of the interphase: the surface layer is a 3D object, while the derived image potentials assume two homogeneous phases separated by a boundary. The leading correction of the image forces that would account for the structure of the surface corresponds to a zeroth moment of the dielectric permittivity at the surface, that is, a surface tangential dielectric polarizability α_t_^S^, see Supplement S2. The surface dielectric permittivity can be nullified by a careful choice of the position of the dividing surface – there exists a location where the surface tangential dielectric permittivity is zero, corresponding to the so-called ‘dielectric surface of water’^22^ (Supplements S2 and S8).

The second issue with eqs 26–31 is that, at small distances, the higher-order terms of the multipole expansion of the energy become significant. This is why we left in the expansion also the *z*_o_^–5^ terms; the comparison between different orders of the multipole expansion, namely O­(*z*_o_^–4^), O­(*z*_o_^–5^) and O­(*z*_o_^–6^), allows us to estimate the error introduced by the missing higher-order terms.

The third issue with eqs 26–31 is that the polarization of the molecule at small distances becomes unphysically strong, i.e. fields and gradients become so large that the first hyperpolarizability and quadrupolarizability become important. The failure is particularly obvious with *u* ^G^ – eqs 28and[Disp-formula eq29] have singularity at small *z*_o_. By contrast, eqs 27 for *u* ^L^ has no singularity but the dipole moment (eq 22) becomes unreasonably strongly depolarized at small distances.

The cavity model has additional issues; first, at small distances, a portion of the cavity protrudes above the dielectric surface, which means that the multipole expansion becomes inaccurate at *z* ∼ *R*_cav_ (see Supplement S7). Moreover, we correct only the leading two terms for the cavity and reaction field effects, which leads to overprediction of the lateral orientation of the molecules in the surface layer.

To deal with these issues, we introduce cutoff distances. We show below that the choice of value of the cutoff distance has only a weak effect on the calculated macroscopic characteristics for the case of point multipole. A multipole in a cavity is more difficult to handle; nevertheless, our analysis below provides a lower and an upper bound for the values of the adsorbed dipole density and the surface potential.

### Cutoff Distance and Continuation of the Dipole Density for Point Multipoles

The formula [Disp-formula eq22] for the depolarization of a molecule in the liquid phase becomes inapplicable nearby the surface. To handle the issue, we introduce a cutoff parameter defined as the maximum acceptable polarization ratio: ξ_cutoff_ = max­(|*p*_*z*_ *−p*_0*z*_|/*p*_*z*_). For water, we consider it unrealistic when ξ_cutoff_ approaches values between 0.1 and 1. Below, we test the values 2/3, 1/2, 1/3, and 1/4. The conservative ξ_cutoff_ = 1/4 corresponds to depolarization of the *p*_*z*_ component by 20% compared to *p*_0*z*_; the hyperpolarizabilities should not have a strong effect at such polarization. In the other end, the value ξ_cutoff_ = 2/3 corresponds to very high allowed polarization. The distance at which ξ_cutoff_ is approached follows from eq 22:

$$2k_{\mathrm{im}}^{L}\alpha_{p}/{\left|2z_{o}\right|}^{3}\approx \xi_{\mathrm{cutoff}}$$ 52

Solving this for *z*_o_ gives for the cutoff distance:

$$z_{\mathrm{cutoff}}^{L}={\left(\frac{1}{16\pi \varepsilon^{L}}\frac{\varepsilon^{L}-\varepsilon^{G}}{\varepsilon^{L}+\varepsilon^{G}}\frac{\alpha_{p}}{\xi_{\mathrm{cutoff}}}\right)}^{1/3}$$ 53

The value is of the order of 0.2 Å (very high polarization allowed) to 0.3 Å (conservative). Similarly, for a molecule in the gas phase:

$$z_{\mathrm{cutoff}}^{G}={\left(\frac{1}{16\pi \varepsilon^{G}}\frac{\varepsilon^{L}-\varepsilon^{G}}{\varepsilon^{L}+\varepsilon^{G}}\frac{\alpha_{p}}{\xi_{\mathrm{cutoff}}}\right)}^{1/3}$$ 54

Due to the smaller dielectric permittivity in the denominator, the cutoff distance in the gas is longer – 0.8 Å (very high polarization allowed) to 1.1 Å (conservative).

In this section, we use a coordinate system where the gas phase occupies *z* > 0 and the liquid is at *z* < 0. For the layer of molecules closest to the surface, between −*z*_cutoff_^L^ and + *z*_cutoff_^G^, the distribution (32) is invalid due to the failure of the expressions for *u* ^L^ and *u* ^G^. Therefore, for −*z*_cutoff_^L^ < *z* < + *z*_cutoff_^G^, we assume a continuous linear transition of all mean characteristics of the polarization, for example:

$${\mathrm{p̅}}_{z}\left(z_{o}\right)=\{\begin{array}{cc} {\mathrm{p̅}}_{z}^{G}\left(z_{o}\right), & z_{o}>z_{\mathrm{cutoff}}^{G};\\ {\mathrm{p̅}}_{z}^{G}\left(z_{\mathrm{cutoff}}^{G}\right)+\left({\mathrm{p̅}}_{z}^{G}\left(z_{\mathrm{cutoff}}^{L}\right)-{\mathrm{p̅}}_{z}^{L}\left(z_{\mathrm{cutoff}}^{G}\right)\right)\frac{z_{o}-z_{\mathrm{cutoff}}^{G}}{z_{\mathrm{cutoff}}^{L}+z_{\mathrm{cutoff}}^{G}}, & z_{\mathrm{cutoff}}^{G}>z_{o}>-z_{\mathrm{cutoff}}^{L};\\ {\mathrm{p̅}}_{z}^{W}\left(z_{o}\right), & z_{o}<-z_{\mathrm{cutoff}}^{L}. \end{array}$$ 55

This linear continuation is plotted as dotted lines in Figure 4 for the average *p*_*z*_, and in Figure 5 for the average *p*_0*i*_^2^.

### Cutoff Distance and Continuation of the Dipole Density for Multipoles in Cavities

Since only the leading terms of the multipole expansion were corrected for the cavity and reaction field effects, the dipole density profile in Figure 4 holds only at relatively large distances, where the normal orientation does not exceed ∼10% yet. Even in this case, we can estimate the lower and the upper bound of the adsorbed dipole density.

As evident from the case of point multipole, in the surface layer, we can expect the normal polarization to increase very quickly from nearly zero to significant orientation. On the liquid side of the dielectric surface, this transition happens within 0.3 Å (Figure 4a). In the gas phase, the profile is better defined–all the different approximations we investigated (different models for *R*_cav_(*C*); with/without α_*p*_; with/without O­(*z*_o_^–5^) terms in *u*; linearized/nonlinear) produce a similar tail where the orientation smoothly increases from very small at 2 Å to significant at 1–1.5 Å above the dielectric surface.

The total adsorbed dipole moment is, as an order of magnitude, equal to (thickness of the adsorbed layer)×(average polarization in the adsorbed layer). Based on the discussion above, the thickness is relatively well-defined – it is no larger than 2.5 Å and no smaller than 1.5 Å. We can also give lower and upper bounds for the average orientation of the dipoles in the surface layer. The upper limit is 100% orientation. The lower limit is set by the orientation produced at the edges of the surface layer, where our multipole expansions are approximately correct – and this is ∼ 5%. The exact shape to the normal polarization profile is not important as it is smoothened by the quadupolar response of the medium, see below.

Hence, for the case of multipole in a cavity, we chose as a cutoff distances the points where the predicted lateral orientation starts to become unrealistically high and produces a drop in the normal dipole moment – these are the maxima in the distributions in Figure 4b, appearing at *z*_cutoff_^L^ = 0.84 Å and *z*_cutoff_^G^ = 1.37 Å for the considered case (*R*_cav_ ∼ ρ^–1/3^, see Supplement S7). The lower bound of the adsorbed dipole density is then given by eq 55, with a linear continuation between the two maxima.

The upper bound corresponds to normal polarization reaching complete orientation in the middle of the adsorbed dipole layer; in this case, we use a parabolic continuation:

$${\mathrm{p̅}}_{z}\left(z_{o}\right)=\{\begin{array}{cc} {\mathrm{p̅}}_{z}^{G}\left(z_{o}\right), & z_{o}>z_{\mathrm{cutoff}}^{G};\\ p_{z}^{\mathrm{surface}}\left(z_{o}\right) & z_{\mathrm{cutoff}}^{G}>z_{o}>-z_{\mathrm{cutoff}}^{L};\\ {\mathrm{p̅}}_{z}^{W}\left(z_{o}\right), & z_{o}<-z_{\mathrm{cutoff}}^{L}, \end{array}$$ 56

where *p*_*z*_^surface^ is a parabola interpolating the two chosen cutoff points and passing through a maximum value of *p*_0_ in the middle between them, see Figure 4b.

We tried other possible continuations and cutoff distances but they lead to similar lower and upper bounds for the surface potential.

### Density Profile of Water

To obtain the integral characteristics of the surface, we need the density profile of water. We assume that the relevant profile is the one that has the contribution of the capillary waves subtracted – the so-called *classical profile of the intrinsic structure theory*,^40^ having the following tanh dependence on *z*:

$$C\left(z;L_{\varepsilon }\right)=\frac{1}{2}\left(C^{L}+C^{G}\right)-\frac{1}{2}\left(C^{L}-C^{G}\right)\tanh\;\frac{z-L_{\varepsilon }}{2L_{C}}$$ 57

Here, *C* ^L^ and *C* ^G^ are the particle densities in the bulk liquid and gas phase; *L*_ε_ is the distance between the dielectric surface (*z* = 0, where α_t_^S^ = 0 and the derived expressions for *u* hold true) and the Gibbs equimolecular surface (*z* = *L*_ε_, where the excess Γ_*C*_ of *C*(*z*) is zero and where *C*(*L*_ε_) = 1/2*C* ^L^ + 1/2*C* ^G^). The distance *L*_ε_ between the equimolecular and the dielectric surface is a macroscopic characteristic of every interface. The calculations below show that the Gibbs surface is on the gas side of the L|G boundary and the dielectric surface is beneath it, in the liquid (so *L*_ε_ > 0). Finally, *L*_*C*_ in eq 57 is a measure of the thickness of the surface layer. From previous works,^24,41^ we know that water at room temperature has a hydrophobic gap of around one Van der Waals radius of water molecule, *R*_w_ = 1.39 Å. This corresponds to structure of the surface with a single layer of water molecules having density reduced by 50% compared to the liquid, meaning that there are *C* ^L^ *R*_w_/2 molecules above the equimolecular surface. On the other hand, if we neglect the concentration in the gas phase in eq 57, for the number of molecules above the equimolecular surface we calculate:

$$C^{L}\int_{0}^{\infty }\left(\frac{1}{2}-\frac{1}{2}\tanh\;\frac{z}{2L_{C}}\right)dz=\ln\left(2\right)L_{C}C^{L}$$ 58

By setting this equal to *C* ^L^ *R*_w_/2, we find that *L*_*C*_ = 0.73×*R*_w_ = 1.0 Å at room temperature. This choice corresponds to value of the so-called *universal amplitude* ^40^ of ξ_+0_ = 0.96×*R*_w_, which is reasonable. It can be compared also to *L*_*C*_ = 1.3 Å from MD,^42^ but the last value is without the amplitude of the capillary waves completely subtracted – only limited by the finite size of the simulation cell.^42^ The simulations by Rashmi et al.^26^ (quantum dynamics with the MB-pol many-body quantum-correction potential) produced a much sharper surface: even with the capillary waves, from their density profile we obtained *L*_*C*_ = 0.8 Å. Rashmi et al. also determined the instantaneous surface using the Willard–Chandler procedure^43^ to subtract the capillary waves; this produces *L*_*C*_ ∼ 0.3 Å – i.e. only around *C* ^L^ *R*_w_/7 of the molecules are above the Gibbs surface. The DFT-MD simulations of Becker et al.^17^ similarly produce a small surface layer thickness (*L*_*C*_ = 0.9 Å before subtracting the capillary waves). Hence, below we also investigate a thinner layer, *L*_*C*_ = 0.5 Å.

### Location of the Dielectric Surface

The first macroscopic characteristic we need to calculate is the location *L*_ε_ of the dielectric surface with respect to the equimolecular one. There are two reasons why the surface layer is of reduced polarizability. The first one is the nonlinear dependence of ε on *C*, see Figures S4 and S5 in Supplement S8. We test two dependences of ε on *C*: one that assumes approximately constant *R*_cav_ and predicts approximately linear ε­(*C*) (producing a small value of *L*_ε_: 0.22 Å), and another that uses Onsager’s *R*_cav_ ∼ *C* ^–1/3^ and produces a nonlinear ε­(*C*) (hence producing a much larger distance between the dielectric and the equimolecular surfaces: *L*_ε_ = 1.33 Å). In both cases, the surface layer is of reduced polarity, hence, the dielectric surface is on the liquid side of the equimolecular one.

The second reason why the surface layer is of reduced polarity is the significant orientation in the surface layer. We calculate the tangential polarizability as (see Supplement S9 for derivation):

$$\begin{array}{c} \alpha_{t}^{S}=\alpha_{p}\Gamma_{w}+\int_{-\infty }^{0}\left[C\left(z;L_{\mathrm{em}}\right)\frac{\overset{®}{\left(p_{0x}^{2}+p_{0y}^{2}\right)}}{2\left(1+k_{\mathrm{im}}^{L}\alpha_{p}/{\left|2z_{o}\right|}^{3}\right)kT}-C^{L}\frac{p_{0}^{2}}{3kT}\right]dz\\ +\int_{0}^{\infty }\left[C\left(z;L_{\mathrm{em}}\right)\frac{\overset{®}{\left(p_{0x}^{2}+p_{0y}^{2}\right)}}{2\left(1-k_{\mathrm{im}}^{G}\alpha_{p}/{\left|2z_{o}\right|}^{3}\right)kT}-C^{G}\frac{p_{0}^{2}}{3kT}\right]dz. \end{array}$$ 59

Here, *C*(*z*;*L*_em_) is the function (eq 57); *L*_em_ is the distance of the surface *z* = 0 from the Gibbs equimolecular surface; Γ_w_ is the adsorption of water at the surface *z* = 0:

$$\Gamma_{w}=\int_{-\infty }^{0}\left[C\left(z;L_{\mathrm{em}}\right)-C^{L}\right]dz+\int_{0}^{\infty }\left[C\left(z;L_{\mathrm{em}}\right)-C^{G}\right]dz$$ 60

In the numerical procedure, we only need to calculate the triple (d*z*dθdφ) integral (eq 59) in some 50–100 points between *z*_cutoff_ and a distance *z*_lin_ large enough for the linear formulas for the average *p*_0*x*_^2^ *+ p*_0*y*_^2^ to be correct; beyond it, a single integration of the approximate linear formulas [Disp-formula eq47] and 48 is sufficient. From Figure 5, |*z*_lin_| ∼ 1 Å in the liquid and ∼ 2 Å in the gas. Between *z*_cutoff_^L^ and *z*_cutoff_^G^, a linear spline is used, of the type of eq 55.

The dielectric surface corresponds to the particular value *L*_em_ = *L*_ε_ which nullifies the lateral surface polarizability α_t_^S^. To find it, we evaluated the integral (eq 59) as a function of *L*_em_. All values of the integral (59) except zero are actually meaningless, because the image potential used for evaluating ρ_φθ_ is incorrect for any surface but the dielectric one, see Supplement S2. The location of the dielectric surface determined as the solution to the equation α_t_^S^(*L*_ε_) = 0, however, is consistent with the precision of our model. The values of *L*_ε_ found in this way are given in Table 3, for various choices of the cutoff parameter ξ_cutoff_. As seen, the location of the dielectric surface varies by some 0.2 Å depending on the choice of ξ_cutoff_. Hence, the location of the dielectric surface is at a distance between 0.2 and 0.4 Å below the equimolecular one.

**Table 3:** Macroscopic Characteristics of the Adsorbed Layer of Dipoles for Point Multipole and Various Choices of the Cutoff Parameter and for Multipole in a Cavity for Two Models of *R*_cav_, Two Values of *L*_*C*_, and Two Continuations (Linear and Parabolic) of the Dependence of the Mean Orientation on *z*

cutoff parameter ξ_cutoff_	*L*_*C*_ [Å]	*L*_**ε**_ [Å]	*P*_0_^S^ [Cm^–1^] × 10^12^	mean location of normal dipole layer [Å]	Δ_L_^G^ϕ [mV]
2/3	1.0	0.18	–10.8	0.30 ± 0.48	
1/2	1.0	0.24	–9.4	0.26 ± 0.51	–33
1/3	1.0	0.32	–9.7	0.24 ± 0.51	–34
1/4	1.0	0.38	–10.0	0.24 ± 0.50	

model for the size of the cavity	*L*_*C*_ [Å]	*L*_ε_ [Å]	*P*_0_^S^ [Cm^–1^] × 10^12^	mean location of normal dipole layer [Å]	Δ_L_^G^ϕ [mV]
*R*_cav_ ∼ const	1.0	0.22	–3.2 (lin.)	0.57 ± 0.86	–16
			–16.6 (parb.)	0.27 ± 0.60	–62
*R*_cav_ ∼ *C* ^–1/3^	1.0	1.33	–2.0 (lin.)	0.48 ± 0.94	–10
			–23.2 (parb.)	0.23 ± 0.54	–86
*R*_cav_ ∼ *C* ^–1/3^	0.5	0.67	–1.6 (lin.)	0.14 ± 0.77	–10
			–20.9 (parb.)	0.09 ± 0.49	–94

In reality, the two effects – nonlinear ε­(*C*) and partial saturation due to the images – are acting together, but are not additive; the larger is probably closer to truth. We anyway tested several values for *L*_ε_; generally, the larger this distance is, the higher the local water density *C* nearby the dielectric surface, and hence more dipoles are orientated by the image forces. The two extreme cases, *L*_ε_ > *L*_*C*_ and *L*_ε_ ≪ *L*_*C*_, produce surface dipole moments that differ by a factor of close to two (corresponding to half the local density when *L*_ε_ ≪ *L*_*C*_).

We tested the role of the order of the multipole expansion on the calculated value of *L*_ε_ by neglecting the ***qq***/*z*_o_^5^ and the ***po***/*z*_o_^5^ terms from *u*, thus comparing the O­(*z*_o_^–5^) and O­(*z*_o_^–6^) expansions. The result is *L*_ε_ moving by 0.2–0.25 Å, depending on the cutoff, which is acceptable (the shift of *L*_ε_ smaller than the thickness of the surface layer).

Our value for *L*_ε_ can be compared with the one of Bonthuis et al.^22^ for the dielectric surface for the interface water|hydrophobic solid. They found *L*_ε_ = −1.4 Å, i.e. their dielectric surface is significantly shifted toward the hydrophobic phase.

### Adsorbed Dipole Moment in the Absence of Field

Once the location of the dielectric surface is determined, we can proceed to the integration of the ‘unperturbed’ dipole moment (not depolarized by the field of the DDL^1^):

$$P_{0}^{S}=\int_{-\infty }^{0}C\left(z;L_{\varepsilon }\right){\mathrm{p̅}}_{z}^{L}dz+\int_{0}^{\infty }C\left(z;L_{\varepsilon }\right){\mathrm{p̅}}_{z}^{G}dz$$ 61

Here, the concentration profile *C*(*z*;*L*_ε_) is given by eq 57; *p̅*_*z*_^L^ and *p̅*_*z*_^G^ are the nonlinear averages, e.g.:

$${\mathrm{p̅}}_{z}^{L}=\int_{0}^{\pi }\int_{0}^{2\pi }p_{z}\rho_{\varphi \theta }\sin\;\theta d\varphi d\theta$$ 62

with *p*_*z*_ from eq 22, ρ_φθ_ from eq 32 (with no dependence on ψ for water), with *u* ^L^ given by eq 26 and *U* coefficient given in explicit form in Supplement S5. As with eq 59 before, for eq 61, the triple integration with respect to *z*, θ and φ is performed only in the regions between *z*_cutoff_ and *z*_lin_; at longer distances, the linear formulas (eq 45) hold, and between *z*_cutoff_^L^ and *z*_cutoff_^G^, a linear or parabolic spline is used, see eqs 55 and 56.

The calculated values of *P*_0_^S^ are listed in Table 3 for point multipole and several choices for the cutoff length. As seen, the choice of ξ_cutoff_ has a small effect on the computed *P*_0_^S^. The value we find is *P*_0_^S^ ≈ −10 × 10^–12^ C·m^–1^ (the negative sign means negative pole toward air). This corresponds to *P*_0_^S^/*p*_0_ = one water dipole per 60 Å^2^; hence, in the top layer of water molecules (of density reduced by ∼ 50%), one out of every 3 molecules is fully orientated.

We tested the applicability of the multipole expansion again by neglecting the ***qq***/*z*_o_^5^ and the ***po***/*z*_o_^5^ terms from *u*. The values of *P*_0_^S^ calculated with O­(*z*_o_^–5^) are in the range −12 ± 2 × 10^–12^ C·m^–1^, depending on the cutoff, and have a somewhat stronger dependence on ξ_cutoff_. The fact that the O­(*z*_o_^–6^) calculation has a reduced dependence on ξ_cutoff_ is a good sign for the convergence of the multipole expansion.

Next, we calculated *P*_0_^S^ for four versions of the cavity model: the two options for the dependence of the cavity radius *R*_cav_ on the local density *C*, and for the two continuations of the normal polarization profile in the surface layer (lower bound – set by linear spline, and upper bound – parabola reaching complete orientation). The values of *P*_0_^S^ are compared in Table 3. This approach limits *P*_0_^S^ between −2 and −23 C·m^–1^.

We also calculated the mean location of the surface layer: using the normal dipole density in Figure 4, we obtain that the adsorbed dipole layer stays 0.25–0.50 Å above the dielectric surface (Table 3), i.e., it is close to the equimolecular surface. The adsorbed layer is rather thick: the standard deviation is ± 0.5 Å (see Figure 4).

Finally, in the last row of Table 3, we investigated the effect of a sharper density profile (*L*_*C*_ = 0.5 Å instead of *L*_*C*_ = 1.0 Å). This change has a relatively small effect on the calculated *P*_0_^S^ (10–20%, depending on the rest of the assumptions), but brings the adsorbed dipole layer closer to the dielectric surface.

### Normal Surface Polarizability

Compared to the tangential component α_t_^S^, the calculation of the normal surface polarizability α_*z*_^S^ is more complex: *E*_*z*_ changes with *z*; the depolarization in the DDL has a major effect on α_*z*_^S^; the nonmonotonic dependence of α_mol,*z*_ makes the integration more strongly dependent on the cutoff parameters. These issues are discussed in more detail in Supplement S9.

Since a detailed model of α_*z*_^S^ is beyond the aims of this work, it will suffice to give an order of magnitude estimate of α_*z*_^S^. We expect a negative value of α_*z*_^S^ at the dielectric surface, because both the layers with significant parallel orientation and the layers with significant orientation in one direction will have reduced α_mol,*z*_ compared to the bulk. The thickness of the layer of strongly orientated molecules can be estimated from eq 44 – the orientation becomes significant when:

$$\frac{{\mathrm{p̅}}_{z}^{L}}{p_{0}}=k_{\mathrm{im}}^{L}\frac{3q_{n,zz}p_{0}}{40kT}\frac{1}{z_{o}^{4}}∼1$$ 63

i.e. the thickness *L*_imm_ of the layer of liquid that does not contribute to the normal polarizability is

$$L_{\mathrm{imm}}∼{\left(k_{\mathrm{im}}^{L}\frac{3q_{n,zz}p_{0}}{40kT}\right)}^{1/4}$$ 64

At room temperature for water, this quantity has the value of *L*_imm_ = 0.24 Å. We can estimate α_*z*_^S^ as the excess of the bulk dielectric permittivity ε^L^:

$$\alpha_{z}^{S}∼-\varepsilon^{L}L_{\mathrm{imm}}+\left(\begin{array}{c} \mathrm{positive contribution}\\ \mathrm{from the}\;\mathrm{gas}\;\mathrm{phase} \end{array}\right)+\left(\begin{array}{c} \mathrm{positive contribution}\\ \mathrm{from}\;\mathrm{DDL}\;\mathrm{depolarization} \end{array}\right)∼-\left(\frac{1}{2}\pm \frac{1}{4}\right)\varepsilon^{L}L_{\mathrm{imm}}$$ 65

The numerical coefficient 1/2 ± 1/4 has been chosen based on the expectation that the positive terms are of similar order of magnitude but smaller than the main ε^L^ *L*_imm_ term.

We have previously assumed^1^ that α_*z*_^S^ > 0, because we were not aware of this immobilization effect. Based on eq 65, we expect α_*z*_^S^ ∼ −10 ± 5 Å, corresponding to surface of zero α_*z*_^S^ located 0.12 ± 0.06 Å under the dielectric surface (where α_t_^S^ = 0). For comparison, Bonthius et al.^22^ found that the surface of zero α_*z*_^S^ is 0.3 Å on the water side of the surface of zero α_t_^S^ for water|hydrophobic solid.

### Depolarization in the DDL and Surface Potential

The above calculations refer to molecules in isolation, without lateral interaction within the surface layer. We now proceed to determine the medium response to the orientation in the surface layer, using the quadrupolar Maxwell equations.^28,44^

The quadrupolar equation of electrostatics (a multipolar series of the exact vacuum equations truncated at the octupolar terms) extends the classical macroscopic Poisson equation (truncated at the quadrupoles). For flat symmetry, the displacement field truncated at octupoles is given by^28,44^

$$D_{z}=\varepsilon_{0}E_{z}+P_{z}-1/2dQ_{zz}/dz$$ 66

For the polarization *P*_*z*_ of the anisotropic surface layer, we can use the equation of state of a pyroelectric:^2^

$$P_{z}=P_{0}+\alpha_{P}E_{z}$$ 67

where *P*_0_ = *C*(*z*)*p̅*_*z*_ is the unperturbed polarization in the absence of external field, with *p* ^–^_*z*_ from Figure 4 and *C*(*z*) from eq 57; α_*P*_(*z*) is the local macroscopic normal polarizability. For the quadrupolarization^45^ *Q*_*zz*_, we ignore the zero-field quadrupole (i.e., adsorbed quadrupole neglected):

$$Q_{zz}=\frac{2}{3}\alpha_{Q}\frac{dE_{z}}{dz}$$ 68

Here, the macroscopic quadrupolarizability (normal component, generally *z*-dependent) is related to the quadrupolar length as

$$\alpha_{Q}=3\varepsilon L_{Q}^{2}$$ 69

Coulomb’s macroscopic law for flat symmetry reads d*D*_*z*_/d*z* = 0, or *D*_*z*_ = 0 in the absence of external field. Therefore, the electrostatic problem for the field in the surface layer simplifies to

$$P_{z}-1/2dQ_{zz}/dz=-\varepsilon_{0}E_{z}$$ 70

The quadrupole term in this equation is large in the surface layer. Ben Naim and Stillinger^19^ and Croxton^20^ used instead the equation *P*_*z*_ = −ε_0_ *E*_*z*_, which is valid near the critical point where the gradients are small; at room temperature, however, d*E*_*z*_/d*z* approaches large values in the surface layer.^46^ This is one of the two reasons why Croxton obtained surface potential of exceedingly high magnitude. The second reason is that he used quadrupole moment values with nonzero trace.

Compared to the results of the classical dipolar electrostatics, two common features of the solutions of the quadrupolar electrostatic law can be pointed out: the regularization of the potential and the damping of the field gradient. A remarkable example of the first effect is the finding^45,47^ that a point charge in quadrupolar medium has finite potential; another is that the electric field at a charged surface is continuous, and the same is valid for the electrostatic potential of an infinitely thin capacitor.^48^

### DDL for Adsorbed Dipole at the Interface between Two Isotropic Media

Let us first assume that we can treat the surface layer as infinitely thin adsorbed layer of dipoles located at the dielectric surface, and the two adjacent phases – as homogeneous isotropic fluids of constant α_*P*_ and α_*Q*_. In this case, we need to solve *D*_*z*_ = 0 in both the L and the G phase.

Two boundary conditions are needed. One is for continuous *E*_*z*_ (the electric field does not jump at the interface between quadupolar media^48,44^ ). The other relates the surface dipole moment to the jump of the bulk quadupolarization^44,49^ (an analogue of Gauss’s equation):

$$\frac{1}{2}Q_{zz}^{G}\left(0\right)-\frac{1}{2}Q_{zz}^{L}\left(0\right)=P^{S}$$ 71

The solution to the problem so set reads:^1^

$$E^{G}=E^{S}\exp\left(-\left|z\right|/L_{Q}^{G}\right);E^{L}=E^{S}\exp\left(-\left|z\right|/L_{Q}^{L}\right)$$ 72

where the value of the surface electric field follows from eqs 68 and 71 as

$$E^{S}=-\frac{P^{S}}{\varepsilon^{L}L_{Q}^{L}+\varepsilon^{G}L_{Q}^{G}}$$ 73

The last formula is analogous to Gouy’s equation^1^ (eq 73) relates surface dipole to surface field, while Gouy’s equation relates surface charge to surface potential). If the gas has negligible quadrupolarizability, eq 73 simplifies to

$$E^{S}=-P^{S}/\varepsilon^{L}L_{Q}^{L}$$ 74

Equation 74 does not define completely the surface field; a surface equation of state for *P* ^S^ is also required. In linear approximation, the adsorbed dipole *P* ^S^ is related to the unperturbed dipole *P*_0_^S^ as

$$P^{S}=P_{0}^{S}+\alpha_{z}^{S}E^{S}$$ 75

Solving eqs 74 and 75 for *P* ^S^ and *E* ^S^ results in

$$P^{S}=\frac{\varepsilon^{L}L_{Q}^{L}}{\varepsilon^{L}L_{Q}^{L}+\alpha_{z}^{S}}P_{0}^{S},E^{S}=-\frac{P_{0}^{S}}{\varepsilon^{L}L_{Q}^{L}+\alpha_{z}^{S}}$$ 76

The field (eq 72) produces orientation in the diffuse dipole layer below the surface, giving rise to a bulk polarization of:

$$P^{L}=\left(\varepsilon^{L}-\varepsilon_{0}\right)E^{S}\exp\left(-\left|z\right|/L_{Q}^{L}\right)$$ 77

Since *E* ^S^ ∝ -*P* ^S^, the dipoles in the diffuse layer point in the opposite direction of the ones in the adsorbed layer, i.e. the positive poles of the dipoles in the diffuse layer point toward the positive pole of the adsorbed dipole, see Figure 7. This may appear counterintuitive – positive pole to positive pole is normally associated with repulsion. The logic of the result is easiest to comprehend by treating the adsorbed dipole as a molecularly thin capacitor. The medium between the two walls of a classical capacitor is polarized by the field between the two plates (∼*E* ^S^) and has the opposite dipole moment of the dipole moment carried by the capacitor plates. However, due to the quadrupoles, the response of the medium becomes nonlocal and the ‘content’ of the capacitor ‘spills’ at a distance *L*_*Q*_ outside its walls.

The integral dipole carried by the diffuse layer is

$$\Gamma_{P}^{L}=\int_{-\infty }^{0}P^{L}dz=\left(\varepsilon^{L}-\varepsilon_{0}\right)E^{S}L_{Q}^{L}=-\left(1-\varepsilon_{0}/\varepsilon^{L}\right)P^{S}$$ 78

The respective total dipole moment of the surface is the sum of what is carried by the adsorbed and the diffuse layers:

$$\Gamma_{P}=P^{S}+\Gamma_{P}^{L}=\frac{\varepsilon_{0}}{\varepsilon^{L}}P^{S}$$ 79

Hence, the response of the medium dramatically reduces the overall polarization – by a factor ε_0_/ε^L^. Substituting here eq 76, and using the general relationship^2^ Δ_L_^G^ϕ = Γ_*P*_/ε_0_, we obtain for the surface potential:^1^

$$\Delta_{L}^{G}\phi =\frac{\Gamma_{P}}{\varepsilon_{0}}=\frac{P^{S}}{\varepsilon^{L}}=\frac{L_{Q}^{L}}{\varepsilon^{L}L_{Q}^{L}+\alpha_{z}^{S}}P_{0}^{S}$$ 80

If *P*_0_^S^ = −10 × 10^–12^ C·m^–1^ and α_*z*_^S^ is neglected, this approximate formula gives Δ_L_^G^ϕ = −15 mV. However, the dielectric saturation in the adsorbed layer results in less efficient depolarization of the surface, which we take into account through the negative value of the normal surface polarizability α_*z*_^S^, see eq 65. Unfortunately, the difference ε^L^ *L*_*Q*_^L^ − |α_*z*_^S^| in eq 80 is quite undetermined. Not only α_*z*_^S^ is uncertain (see eq 65); the value of the quadrupolar length is also not yet known for water. Water’s *L*_*Q*_^L^ obtained theoretically from the quadupolar cavity model^38^ is ∼0.3 Å, and disagrees with estimates from various properties of electrolyte solutions,^45,50^ which produce *L*_*Q*_^L^ > 1 Å. This discrepancy appears to be related to strong nonlinear effects in very polar fluids like water,^38^ which tend to accumulate surface dipole at the surface of the cavity (for less polar fluids, e.g., methanol, the agreement is adequate). Various choices for the value of *L*_*Q*_^L^ and α_*z*_^S^ produce potential in the range Δ_L_^G^ϕ = −20··· −45 mV (oxygen toward the gas phase).

### DDL Structure for Adsorbed Dipole Distributed in a Finite-Size Surface Layer

Equation 80 is useful for qualitative discussion of the factors that control the surface potential. It shows that the depolarization greatly reduces the surface potential (|Δ_L_^G^ϕ| = |Γ_*P*_|/ε_0_ ≪ |*P*_0_^S^|/ε_0_). The possible values of *L*_*Q*_^L^ for polar-quadrupolar liquids^37,38^ are of limited range, e.g., between 0.2 and 1 Å; hence, eq 80 predicts a relatively weak dependence on *L*_*Q*_^L^ (unless ε^L^ *L*_*Q*_^L^ − |α_*z*_^S^| is small – but the approximations for the derivation fail in the vicinity of ε^L^ *L*_*Q*_^L^ = |α_*z*_^S^|).

However, eq 80 is based on the assumption that the thickness of the adsorbed layer of dipoles is smaller than *L*_*Q*_; in fact, both quantities are of the order of 1 Å. This results in a significant overlap between the adsorbed and the diffuse layer (a similar overlap between the adsorbed charge and diffuse charge layers is important for the EDL in concentrated electrolyte solutions^24^). To investigate the overlap in the DDL, we need to use the actual calculated distribution *P*_0_(*z*) from Figure 4 and the continuous eq 70 of quadrupolar electrostatics, together with the equation of state (eqs 67–69). We now proceed to this task.

To set up the continuous model of the interphase and solve eqs 67–70, we need *P*_0_(*z*), α_*P*_(*z*) and *L*_*Q*_(*z*) as explicit functions of *z*. For *P*_0_(*z*), we use the calculated distribution of *p̅*_*z*_(*z*) shown in Figure 4 (handled as a linear spline function over 300 points between −1 and 3 Å, and with the linearized approximation (45) outside this range). *P*_0_ and *p̅*_*z*_ are related as

$$P_{0}\left(z\right)=C\left(z\right){\mathrm{p̅}}_{z}\left(z\right)$$ 81

Here, *C*(*z*) is the water concentration profile (57). The profile *P*_0_(*z*) of the unperturbed adsorbed dipole is illustrated in Figure 6.

**Figure 6:**
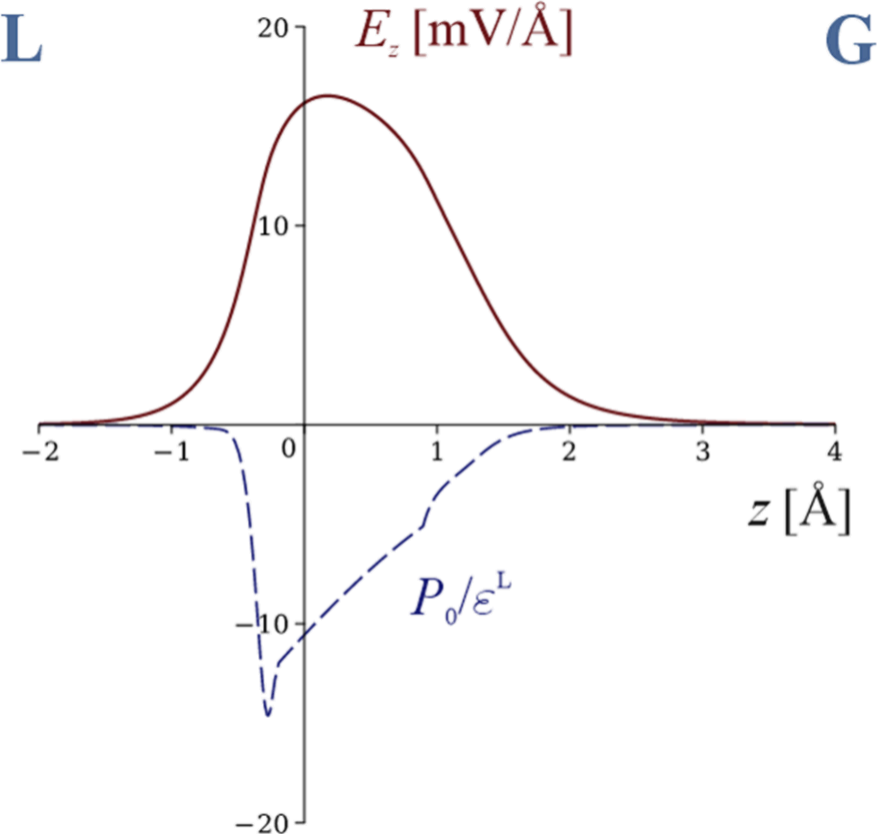
Electric field in the surface layer: numerical solution to the quadrupolar equation of electrostatics (eq 70). For comparison, we plot also the source of the filed: the unperturbed polarization *P*_0_(*z*) = *C*(*z*)*p̅*_*z*_(*z*), converted to units mV/Å by dividing by ε^L^. Point multipole model, ξ_cutoff_ = 1/2.

Next, we tried several variants for the dependence of α_*P*_ and α_*Q*_ on *z*. As a simplest option, we assumed that the macroscopic polarizability α_*P*_ and quadrupolarizability α_*Q*_ also follow the profile (57) of *C*, but with a lag of *L*_ε_:

$$\alpha_{P}=\frac{\varepsilon^{L}-\varepsilon_{0}}{2}\left(1-\tanh\;\frac{z}{2L_{C}}\right);\alpha_{Q}=\frac{3}{2}\varepsilon^{L}{\left(L_{Q}^{L}\right)}^{2}\left(1-\tanh\;\frac{z}{2L_{C}}\right)$$ 82

Here, *z* = 0 is the dielectric surface (not the equimolecular). The lag makes sure that the excess of α_*P*_ at the dielectric surface is zero. As a more advanced model, we used the dependence of α_*P*_ and α_*Q*_ on *C* predicted by the quadrupolar version of the Onsager cavity model, see the Supplement S8 and the profiles in Figure S5.

The first set of the study cases we investigated was for point multipole and tanh profiles of α_*P*_ and α_*Q*_. In it, we substitute eq 81 and 82 in eq 67 and 68. The second set of study cases was for image force for multipole in a cavity, with α_*P*_(*z*) and α_*Q*_(*z*) calculated also from the cavity model, see Supplement S8. In both cases, Coulomb’s law (70) becomes an explicit second order differential equation for *E*_*z*_(*z*). The boundary conditions for the numerical procedure are for zero field far from the interface; *E*_*z*_(*z* = −4 Å) = 0 and *E*_*z*_(*z* = +6 Å) = 0 were found to be sufficiently ‘far’ via a direct test. We used Maple’s default trapezoid method with Richardson extrapolation, with 500–1000 mesh points.

We tested several sets of parameter values for this problem. The base case was chosen to be ξ_cutoff_ = 1/2, respectively *L*_ε_ = 0.24 Å (see Table 3); we use ε^L^ = 78.4 × ε_0_, *L*_*Q*_^L^ = 0.27 Å at *T* = 25 °C, as obtained from our dielectric cavity model;^38,37^ *C* ^L^ = 3.33 × 10^28^ m^–3^ (density 997 kg/m^3^); *L*_*C*_ = 1 Å, see eq 58. We also tested the values for ξ_cutoff_ = 1/3, and higher values of *L*_*Q*_^L^ (up to 0.5 Å), to check the uncertainty of the final result for the surface potential Δ_L_^G^ϕ. We also investigated larger and smaller gap *L*_ε_, as a continuous way to account of the effect from the dielectric saturation in the surface layer (which we took into account through α_*z*_^S^ in the previous paragraph, see eqs 65 and 75). The most interesting results from this analysis are reported in Table 3 and Table S1 in Supplement S10 for the point multipole method (with tanh dependences of α_*P*_ and α_*Q*_ on *z*), and in Table 3 for multipoles in a cavities (with α_*P*_ and α_*Q*_ as predicted by the cavity model, see Supplement S8).

The solution for *E*_*z*_(*z*) for a point multipole is shown in Figure 6. The field intensity is of the order of 10^8^ V/m. The profile of the unperturbed polarization *P*_0_(*z*) produced by the image forces is not a smooth function because of the linear continuation we are using between −*z*_cutoff_^L^ and +*z*_cutoff_^G^, see Figure 4 and eq 55. Nevertheless, *E*_*z*_(*z*) is smooth; this is a general feature of the quadrupolar electrostatics – fields are smoothened to a length scale ∼*L*_*Q*_. This smoothening diminishes the role of the cutoff parameter – the field profile changes little for ξ_cutoff_ = 1/3, see Supplement S10; Δ_L_^G^ϕ changes even less.

The calculated distribution of the dipole moment density *P*_*z*_ for a point multipole is plotted in Figure 7. The unperturbed image force polarization *P*_0_ produces electric field *E*_*z*_, which depolarizes the surface. The total polarization *P*_*z*_ has one peak where the adsorbed dipoles dominate, and a second where the diffuse dipoles dominate – the essence of the DDL structure. The total dipole moment of the diffuse layer is nearly equal to that of the adsorbed layer, i.e. the total dipole moment is small (|Γ_*P*_| ≪ |*P* ^S^|). This two-layer structure is prominent in MD simulations,^21^ even without the subtraction of the capillary wave profile.

**Figure 7:**
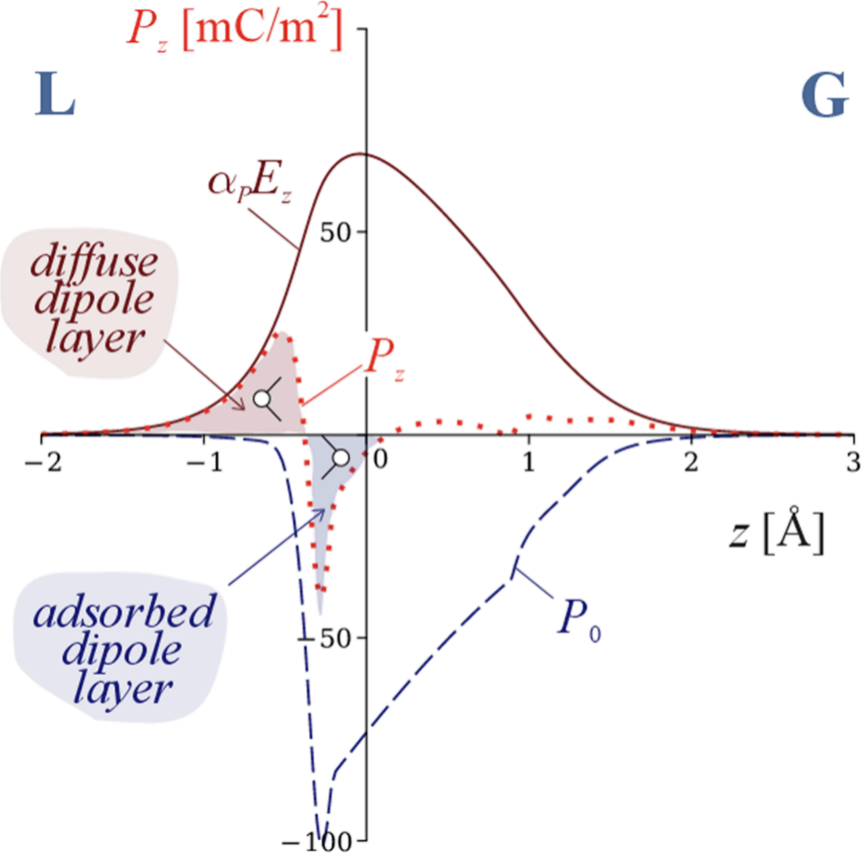
Structure of the dipolar double layer: density of dipole moment in the surface layer. The polarization *P*_0_ due to the image forces is the reason for the polarization in the first place and corresponds to water pointing with the oxygen toward the gas phase. However, the adsorbed dipoles create the ‘spilled capacitor’ field *E*_*z*_, which produces reverse orientation of the water molecules (α_*P*_ *E*_*z*_) in the diffuse layer of dipoles and significant depolarization of the surface. The total polarization *P*_*z*_ from the pyroelectric equation of state (eq 75) corresponds to a double layer structure: two layers of oppositely orientated dipoles separated by a distance ∼*L*_*Q*_^L^.

A new feature of the continuous treatment of the surface layer is the formation of a diffuse layer also on the gas-phase side of the adsorbed dipole layer (slightly positive *P*_*z*_ for *z* > 0 in Figure 7). This happens because the profile α_*Q*_ from eq 82 spreads to a distance *L*_*C*_ away from the surface, and *L*_*C*_ is comparable to the thickness of the adsorbed dipole layer – i.e. there is a sufficient number of ‘gas’ molecules above the dielectric surface to produce a quadrupolar response. The effect is related to our assumption that α_*Q*_ and α_*P*_ change proportionally to each other, eq 82: the local value *L*_*Q*_ = [α_*Q*_/3­(α_*P*_ + ε_0_)]^1/2^ ≈ (α_*Q*_/3α_*P*_)^1/2^ changes little in the surface layer, even well into the gas phase; the same behavior follow from the cavity model for α_*P*_ and α_*Q*_, see Supplement S8. Such dipolar triple layer, with two diffuse layers on the two sides of the adsorbed layer, has been proposed before for dense surfactant monolayers (eq 19 in ref 11). There, the layer of dipoles is between water phase and a layer of hydrocarbon tails which is sufficiently thick to have a quadrupolar length of its own, and results in additional ‘quadrupolar screening’ of the potential of the adsorbed dipoles.^11^ Of course, when the second phase is not gas but a dense fluid, dipolar triple layer always forms, with two diffuse layers in the two phases.^1^

Finally, the surface potential is calculated as

$$\Delta_{L}^{G}\phi =-\int_{-\infty }^{\infty }E_{z}dz$$ 83

which is equivalent to eq 1. For a point multipole and ξ_cutoff_ = 1/2, the integration results in Δ_L_^G^ϕ = −33 mV. For the cavity models, we obtain Δ_L_^G^ϕ = −50 ± 40 mV, see Table 3.

The main reason for the larger surface potential compared to Δ_L_^G^ϕ= −15 mV from the simple formula [Disp-formula eq80] (if α_*z*_^S^ is neglected) is that the local value of α_*P*_ in the surface layer is reduced by about half compared to bulk water due to *L*_*C*_ > *L*_*Q*_, see eq 82.

We tested how the choice of parameter values affects the computed surface potential, see Table 3 and Table S1 in Supplement S10. We found that the values of the cutoff parameter ξ_cutoff_, the quadrupolar length *L*_*Q*_^L^ of the bulk water, and the characteristic thickness *L*_*C*_ of the surface layer all have only a minor effect on the surface potential – for the range of values we consider most likely (ξ_cutoff_ = 1/2···1/3, *L*_*Q*_^L^ up to 0.5 Å, *L*_*C*_ up to 1.5 Å), the surface potential changes by 1–2 mV. More important are the structural characteristics of the surface layer: if we neglect *L*_ε_, or if we add 0.1–0.3 Å of immobilized layer of dipoles that results in location of the surface of zero α_*z*_^S^ 0.1–0.3 Å below the dielectric interface (see Supplement S10), we can obtain Δ_L_^G^ϕ in the range from −30 to −40 mV.

The cavity models predict longer-ranged image potential, but also more significant cavity depolarization. The two limits we investigate correspond essentially to two hypotheses about which effect dominates in the surface layer: little depolarization would produce higher potentials (e.g., Δ_L_^G^ϕ = −70 ± 20 mV), but if α_*p*,cav_ remains large in the surface layer, the potential is weaker (e.g., Δ_L_^G^ϕ = −30 ± 20 mV). The comparison with simulations data is in better agreement with the first situation.

## Results and Discussion

The three-layer structure of the DDL at the surface of water is spectacularly confirmed by the simulation of a water surface of Rashmi et al.^26^ They used quantum dynamics simulation (with temperature-elevated path-integral coarse graining and MB-pol many-body potential energy function). Importantly, they used the Willard-Chandler procedure^43^ to deal with the capillary waves and calculate the instantaneous surface.

In Figure 8, we compare our results for *P*_*z*_/*Cp*_0_ from two versions of the multipole-in-a-cavity model (with parabolic continuation in the surface layer, i.e. the large orientation limit) to simulation results^26,16,17^ for cosθ (where θ is angle between the dipoles and the normal to the surface). The two quantities are equivalent for nonpolarizable molecules only, but the comparison is qualitative anyway. There is a good qualitative agreement, in particular with the results of Rashmi et al., which show both diffuse layer peaks. The location and the height of the adsorption layer peak are in better agreement with the simulations for the cavity model with *R*_cav_ ∼ ρ^–1/3^, compared to *R*_cav_ ∼ *const*. This means that a larger value of *L*_ε_ agrees better with the simulation results. Also, the simulations generally predict a much wider adsorption peak, and a less developed diffuse layer on the liquid side of the equimolecular surface.

**Figure 8:**
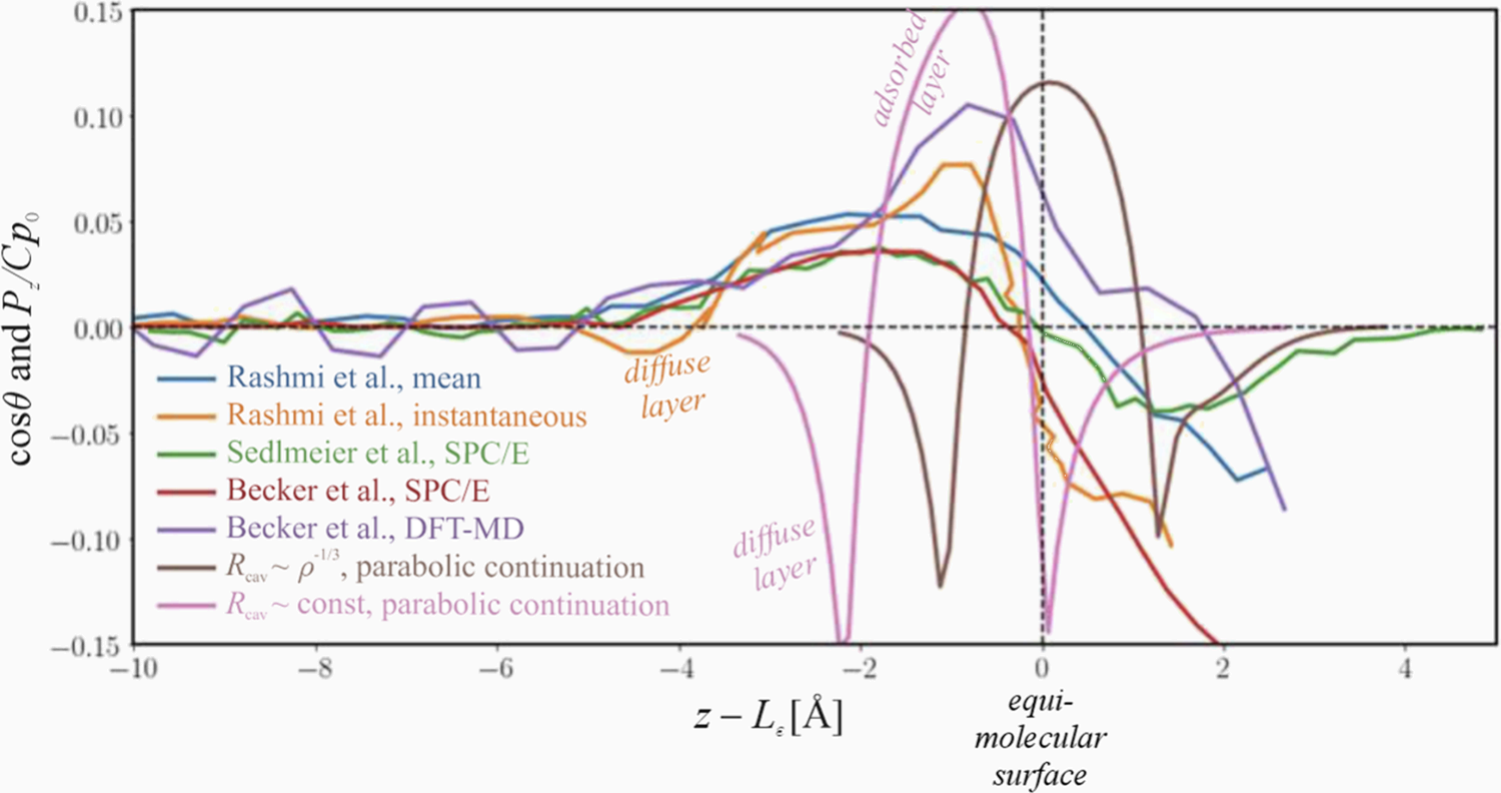
Structure of the dipolar double layer: *P*_*z*_/*Cp*_0_ from our multipole-in-a-cavity model compared to simulated^26,16,17^ mean cosθ as a function of the distance to the equimolecular surface.

The data of Sedlmeier et al.^16^ and Becker et al.^17^ have not been corrected for the capillary waves. This smears the distribution of the dipole moment; therefore, they did not detect the diffuse layer on the liquid side of the equimolecular surface. However, they detected it on the gas side in all cases. The DDL structure on the liquid side is evident in the simulations of Sedlmeier et al.^16^ of solid|liquid interfaces, where the capillary waves are not an issue – see, e.g., their Figure 15.

### Unperturbed Normal Surface Dipole Moment

The values of *P*_0_^S^ calculated in Table 3 can be tested experimentally by nullifying the dipole moment of the surface with an appropriate surfactant monolayer, i.e. by determination of the point of zero dipole, pzd, of the surface. If an insoluble alkanol monolayer is spread on the surface of water, the alkanol molecules are orientated by the hydrophobic force with the −C−O− bond toward the water, i.e. the opposite orientation of that of water.

The absolute dipole moment of the −CH_2_OH moiety is^51^ *p*_ROH_ = 5.5 × 10^–30^ C·m. Assuming that the hydrocarbon tail stands normal to the surface and the OH group orientates parallel to the surface (which maximizes the normal dipole and minimizes the energy of −CH_2_OH in the surface layer), we obtain for the normal component *p*_ROH_sin­(109°/2) = +4.5 × 10^–30^ C·m. Moreover, each OH group in the surface layer substitutes one water molecule, contributing further 0.33×*p*_0_ = +2.1 × 10^–30^ C·m to the dipole increment (0.33×*p*_0_ being the average contribution of a water molecule in the surface layer to *P*_0_^S^). Therefore, we find Δ*p*_ROH_ = 4.5 + 2.1 = +6.6 × 10^–30^ C·m increment of the surface dipole per alcohol molecule. Making slightly different assumptions for the orientation of −CH_2_OH and the displacement of water, we can estimate the increment at Δ*p*_ROH_ = +(4···8)×10^–30^ C·m per molecule.

The pzd of dodecanol was estimated experimentally by analyzing the tendency of Na^+^ and Cl^–^ to stick to it.^11^ For dense dodecanol monolayers (*P*_0_^S^ positive toward air), the EDL potential was found to be positive (Na^+^ adsorbs to −CH_2_OH due to the negative pole being orientated toward the liquid). For dilute monolayers (negative *P*_0_^S^), the EDL potential is negative (Cl^–^ adsorbs). By assuming that the point of zero charge (no preferential adsorption of Na^+^ or Cl^–^) occurs nearby the point of zero dipole, we estimated Γ_s,pzd_ at 0.7–2.0 nm^–2^. Therefore, the adsorbed dipole of pure water can be estimated as *P*_0_^S^ = −Γ_s,pzd_×Δ*p*_ROH_ = 8 ± 5 × 10^–12^ Cm^–1^. This shows that the calculated *P* ^S^_0_ values in Table 3 are reasonable – the experimental estimate indeed lies between the linear and parabolic continuation limits obtained with the cavity models.

### Surface Potential

The uncertainty of the surface potentials we computed is large, but so are the available experimental estimates.^14,15,25^ The value that follows from our model compares well with our own estimate, −90 ± 60 mV from analysis of Δχ data for a number of simple electrolytes.^24^ It also agrees well with the experimental estimates of Gomer and Tryson^52^ (−50 mV) and Farrel and McTique^53^ (−15···-35 mV), but is somewhat lower than the value of Randles and Schiffrin^54^ (−70···-130 mV from temperature dependence). It should be highlighted that the assumption that Δ_L_^G^ϕ is linear function of *T* up to the critical point^53,54^ is questionable from the viewpoint of eqs 43, 44,57, and 80, because ε^L^ and *L*_*C*_ depend nonlinearly on the temperature.

The simulations generally produce much larger potentials. From the data on Figure 1 of Rashmi et al.,^26^ we estimated that their potential is around −350 mV; this demonstrates again that the simulation predicts less developed dipolar diffuse layer then our model. The classical MD simulations of Sedlmeier et al.^16^ (nonpolarizable SPC/E water) produces −240 mV; Doyle et al.^13^ also investigated SPC/E and got −329 mV for the dipolar potential of a curved surface and −284 mV for the total potential (which includes a contribution from *Q*_*rr*_ at curved surfaces). Becker et al.^17^ obtained −240 mV for SPC/E, and −540 mV from DFT-MD.

## Conclusions

We developed a O­(*z*_o_^–6^) multipole expansion of the image energy of a molecule near an interface, including dipole, quadrupole and octupole terms. The molecular polarizability is also accounted for, as well as cavity and reaction field effects.

This expansion is used to determine the orientation of a simple molecule near an interface, in particular – a water molecule near the surface of water (Figure 3). The leading term in the multipolar series of the image force that can cause normal orientation is ∝ ***pq***/*z*_o_^–4^, as realized by Frenkel.^18^ Of course, the distribution of the orientation is useful beyond the normal dipole. The distribution is an explicit function of the Euler angles and the distance from the surface (Supplement S5), hence, it is a useful benchmark for MD simulation studies of surfaces that focus on surface orientation, and sum frequency generation studies.^31,26,55^ We show that the so-called dangling OH bonds at the surface are strongly affected by the ***qq***/*z*_o_^–5^ and ***po***/*z*_o_^–5^ terms of the image interaction, and by the location of the molecule with respect to the dielectric surface.

We calculated the macroscopic characteristics of the polarized surface of a simple fluid, including the location of the dielectric surface (where the lateral surface polarizability is zero) and the value of the unperturbed dipole moment density. Finally, we integrated the quadrupolar equation of electrostatics to find the distribution of the electric field in the surface layer and to investigate the depolarization due to the formation of the dipolar double layer^1^ (Figures 6 and[Fig fig7]).

The structure of the dipolar layer is predicted to have a diffuse layer of oppositely orientated dipoles on both sides of the dipolar adsorbed layer. This prediction is in qualitative agreement with recent simulation data (Figure 8).

The calculated values of the unperturbed dipole moment (*P*_0_^S^ = −10 ± 8 × 10^–12^ C·m^–1^) and the surface potential (Δ_L_^G^ϕ= −50 ± 35 mV) agree reasonably well with existing experimental estimates. The order of magnitude of *P*_0_^S^ is confirmed by experimentally estimated point of zero dipole of alcohol monolayers.^11^ The order of magnitude of our calculated Δ_L_^G^ϕ falls within the uncertainty range of several values extracted from various experimental data.^24,52,53^

Possible future work includes:

1. analysis of the temperature dependence of *P* ^S^, and calculation of the temperature derivative Δ_L_^G^ϕ, which is accessible experimentally.^53,54^ In particular, it is important to test the assumption that Δ_L_^G^ϕ decreases linearly until the critical point.
2. Other liquids. The model developed here should work better for liquids less polar than water; it is also easy to extend to liquid mixtures and to interfaces between water and various nonpolar solvents.
3. Adding higher-order terms in the multipole expansion; ***qo***/*z*_o_^–6^ and ***ph***/*z*_o_^–6^ may have large contribution to the surface potential, in particular for water, due to the small value of *q*_*zz*_ of water. This is not trivial as higher-order terms require also account for structural factors in the image force expressions (higher order surface polarizabilities, cf. Supplement S2), and possibly taking into account the image force caused by the difference in macroscopic quadrupolarizabilities of the two phases.
4. We can use the developed framework to model the orientation of ion-dipoles near the water surface. There is evidence that this orientation has a significant effect on the surface tension and the Δχ potential of concentrated electrolytes.^7^
5. The surface dipolar potential and the DDL must give rise to an adsorption potential and a disjoining pressure that may be significant, especially when the nonpolar phase is quadrupolar (benzene, dense-phase CO_2_).

## Supplementary Material



## Notes

S1: list of symbols, indices and abbreviations; S2: effect of the tangential surface polarizability on the image force; S3: image field and field gradients; S4: explicit expressions for the orientation distribution of water; S5: conversion of multipole moments upon changing the origin; S6: structure of the surface layer for polarizable molecules; S7: effect of the cavity field and the reaction field on the image force; S8: local values of the dielectric properties (ε, *L*_*Q*_, and *R*_cav_); S9: molecular model of the surface polarizabilities; S10: structure of the DDL with explicit distribution of the surface dipole moment (PDF)
