# Supplementary material for: Surface Potential and Surface Dipole Moment of Water and Polar-Quadrupolar Liquids
Source: Langmuir. 2026 Jun 30;42(27):19466–87. doi: 10.1021/acs.langmuir.6c00269 (PMC13374380; doi:10.1021/acs.langmuir.6c00269)
Supplement: Supplementary file 1 [file la6c00269_si_001.pdf]

Surface potential and surface dipole moment of water and polar-quadrupolar liquids (supplementary information)

Radomir I. Slavchov[1], Boyan Peychev[1,2], Iglika M. Dimitrova[3,2]

[1] School of Engineering and Materials Science, Queen Mary University of London, Mile End Road, London E1 4NS, UK
[2] Institute of Physical Chemistry, Bulgarian Academy of Sciences, Akad. G. Bonchev Str., bl. 11, ZIP 1113, Sofia
[3] Faculty of Chemical Technologies, Department of Physical Chemistry, University of Chemical Technology and Metallurgy, 8, Kliment Ohridski blvd, ZIP 1756, Sofia, Bulgaria

1. List of symbols, indices and abbreviations

C	particle number density (in units [m^{-3}])
c_n	normalization coefficient
$\boldsymbol{D}$	electric displacement field
D_i	component of $\boldsymbol{D}$
d	distance between the oxygen nucleus and the centre of mass of water
$\mathbf{E}^{\varphi\psi\theta}$	Euler tensor
$\boldsymbol{E}$	electric field intensity
$\boldsymbol{E}_\mathrm{cav}$	cavity field
E_i	component of $\boldsymbol{E}$
$\boldsymbol{E}_\mathrm{react}$	reaction field
$\mathbf{e}_i$	unit vector in direction i
e	charge
e_im	image charge
$\boldsymbol{E}^\mathrm{S}$	surface electric field intensity, $\boldsymbol{E}(z=0)$
$f_p, f_q, f_E, f_{\nabla E}$	quadupolar factors in the cavity/reaction field fromulae, eq. (104)&(113)
$g_p, g_q, g_{\nabla E}$	quadupolar factors in the cavity/reaction field fromulae, eq. (104)&(113)
$\mathbf{h}$	molecular hexadecapole moment
h_{ijk}	component of $\mathbf{h}$
k	Boltzmann constant
k_im	dielectric factor, eq. (5)&(19)
L	length
L_C	thickness of the surface layer
L_imm	thickness of the layer where dielectric saturation occurs
L_Q	quadrupolar length
L_ε	distance between the dielectric surface and the equimolecular surface

m	molecular mass of water
$\boldsymbol{o}$	molecular octupole moment
o_{ijk}	component of $\boldsymbol{o}$
$\boldsymbol{P}$	polarization (macroscopic density of dipole moment), C·m^{-2}
P_i	component of $\boldsymbol{P}$
$\boldsymbol{P}_0$	unperturbed image force polarization in the surface layer (only a normal component, $\boldsymbol{P}_0 = P_0\mathbf{e}_z$), C·m^{-2}
P^{S}	intrinsic surface normal dipole moment, excess of $\boldsymbol{P}$, C·m^{-1}
P_0^{S}	unperturbed dipole moment of the surface (in the absence of surface reaction field), excess of P_0
$\boldsymbol{p}$	dipole moment
$\boldsymbol{p}_0$	dipole moment in the absence of electric field and molecular polarization
p_i	component of the dipole moment $\boldsymbol{p}$
p_{0i}	component of the dipole moment $\boldsymbol{p}_0$
$\boldsymbol{Q}$	quadrupolarization tensor (macroscopic density of quadrupole moment)
Q_{ij}	component of the quadrupolarization $\boldsymbol{Q}$
$\boldsymbol{q}$	molecular quadrupole moment
q_{ij}	component of $\boldsymbol{q}$
R_{cav}	radius of the cavity
R_{w}	Van der Waals radius of water
$\boldsymbol{r}$	radius-vector
$\boldsymbol{r}_{\mathrm{o}}$	radius-vector of the point multipole
T	temperature
$\mathbf{U}$	unit tensor
U_{pp0}, U_{pq}, U_{qq}, U_{po}	coefficients in the expansions (13)&(18) of the interaction energy
U_{pp}	coefficient with polarization, eq. (27)&(28)
u	free energy of interaction of a multipole with an interface
V	volume
X_p	reaction field coefficient ($E_{\mathrm{react}} = X_p\boldsymbol{p}$)
X_q	reaction field gradient coefficient ($\nabla E_{\mathrm{react}} = X_q\boldsymbol{q}$)
x	Cartesian coordinate tangential to the interface
Y_E	cavity field coefficient ($\boldsymbol{E}_{\mathrm{cav}} = Y_E\boldsymbol{E}_{\mathrm{im}}$)
$Y_{\nabla E}$	cavity field gradient coefficient ($\nabla\boldsymbol{E}_{\mathrm{cav}} = Y_{\nabla E}\nabla\boldsymbol{E}_{\mathrm{im}}$)
y	Cartesian coordinate tangential to the interface
z	Cartesian coordinate normal to the interface
z_{o}	distance between the dielectric surface and the point multipole

$\alpha_{\mathrm{mol,t}}$, $\alpha_{\mathrm{mol},z}$	effective polarizability per molecule, tangential or normal, eq. (51)
α_P	macroscopic polarizability
α_p	polarizability of a molecule
$\alpha_{p,\mathrm{cav}}$	polarizability of the assembly molecule + cavity, eq. (30)
α_Q	macroscopic quadrupolarizability
α_q	quadrupolarizability of a molecule
$\alpha_{\mathrm{t}}^{\mathrm{S}}$ and α_z^{S}	components of the intrinsic surface polarizability tensor
Γ_{w}	adsorption of water molecules

Γ_P total surface dipole moment (adsorbed dipole P^{S} plus diffuse dipole)
$\Delta_{\mathrm{L}}^{\mathrm{G}}\phi$ potential drop through the interface, $\Delta_{\mathrm{L}}^{\mathrm{G}}\phi = \phi^{\mathrm{G}} - \phi^{\mathrm{L}}$ (Bethe potential excluded)
ΔV change $\Delta_{\mathrm{L}}^{\mathrm{G}}\phi$ of upon spreading a monolayer, $\Delta_{\mathrm{L}}^{\mathrm{G}}\phi$(with monolayer) - $\Delta_{\mathrm{L}}^{\mathrm{G}}\phi$(pure)
$\Delta\chi$ change $\Delta_{\mathrm{L}}^{\mathrm{G}}\phi$ of upon addition of electrolyte, $\Delta_{\mathrm{L}}^{\mathrm{G}}\phi$(with electrolyte) - $\Delta_{\mathrm{L}}^{\mathrm{G}}\phi$(pure)
δ_{ij} Kronecker delta function
ε absolute dielectric permittivity
ε_0 dielectric permittivity of vacuum
θ Euler angle, angle between $\boldsymbol{p}_0$ and $\mathbf{e}_z$
ξ shorthand for L_Q/R_{cav}
ξ_{cutoff} cutoff parameter, $\xi_{\mathrm{cutoff}} = |p_z - p_{0z}|/p_z$ maximum allowed
ρ mass density
$\rho_{\phi\psi\theta}$ density of probability distribution of the Euler angles
ρ_{molecule} charge density of the molecule
ϕ potential of the electric field
φ Euler angle (polar for rotation around the axis of $\boldsymbol{p}_0$)
ψ Euler angle (polar angle for the rotation around the axis z)

Indices:

X_0 in the absence of field or neglecting the polarizability
X_{cav} related to the cavity
X_{cutoff} related to the cutoff distance or criterion
X_e charge
X_{ext} external dipole/quadrupole (total for central molecule + cavity)
X^{G} 'gas' (less polar) phase
X_{im} image
X^{L} liquid (more polar) phase
X_m origin of the dipole-quadupole-octupole at the centre of mass
X_{n} a chosen normal orientation and origin of the dipole-quadupole-octupole
X_{O} origin of the dipole-quadupole-octupole at the centre of the oxygen atom (but orientation is not necessarily normal)
X_{o} at the origin (the location of the point multipole)
X_o octupole
X_p dipole
X_q quadrupole
X_{react} related to the reaction field
X^{S} value for the adsorption layer of dipoles or at the dielectric surface

$\Delta_{\mathrm{L}}^{\mathrm{G}}X$ surface jump of X, $X^{\mathrm{G}} - X^{\mathrm{L}}$
$\bar{X}$ average over all orientations,

$$\bar{X} = \int_0^{\pi}\int_0^{2\pi}\int_0^{2\pi} X\rho_{\varphi\psi\theta}\sin\theta\,\mathrm{d}\varphi\,\mathrm{d}\psi\,\mathrm{d}\theta$$

Abbreviations:

DDL	dipolar double layer
EDL	electric double layer
G	gas phase
L	liquid phase
MD	molecular dynamics
pzc	point of zero charge
pzd	point of zero dipole

2. Effect of the tangential surface polarizability on the image force

If the boundary surface between two dielectrics has a nonzero tangential surface polarizability, this modifies the boundary condition at the surface[29,56]:

$$\nabla_{\mathrm{t}} \cdot \alpha_{\mathrm{t}}^{\mathrm{S}} \boldsymbol{E} + D_{\mathrm{n}}^{\mathrm{G}} - D_{\mathrm{n}}^{\mathrm{L}} = 0, \tag{84}$$

where ∇_{t} is the surface nabla operator (only the components of ∇ tangential to the surface). We have chosen a coordinate system where $\mathbf{e}_z$ points at the gas phase and the surface is $z = 0$. For a point charge near a surface (cylindrical symmetry), eq. (84) reads:

$$\alpha_{\mathrm{t}}^{\mathrm{S}} \frac{1}{r} \frac{\partial}{\partial r} r E_r + D_z^{\mathrm{G}} - D_z^{\mathrm{L}} = 0 .$$

This boundary condition leads[29,56] to the following image potential:

$$\phi_{\mathrm{im}} = \frac{e}{4\pi\varepsilon^{\mathrm{L}}} \int_0^\infty \frac{\varepsilon^{\mathrm{L}} - \varepsilon^{\mathrm{G}} - \alpha_{\mathrm{t}}^{\mathrm{S}} \xi}{\varepsilon^{\mathrm{L}} + \varepsilon^{\mathrm{G}} + \alpha_{\mathrm{t}}^{\mathrm{S}} \xi} \mathrm{e}^{-\xi(z+z_{\mathrm{o}})} \mathrm{J}_0(\xi r) \mathrm{d}\xi = \frac{e}{2\pi} \int_0^\infty \frac{\mathrm{e}^{-\xi(z+z_{\mathrm{o}})} \mathrm{J}_0(\xi r) \mathrm{d}\xi}{\varepsilon^{\mathrm{L}} + \varepsilon^{\mathrm{G}} + \alpha_{\mathrm{t}}^{\mathrm{S}} \xi} - \frac{e}{4\pi\varepsilon^{\mathrm{L}}} \frac{1}{\sqrt{r^2 + (z+z_{\mathrm{o}})^2}} . \tag{85}$$

Here, J_0 is the Bessel J function of the 0th order. At zero surface polarizability ($\alpha_{\mathrm{t}}^{\mathrm{S}} = 0$), this expression simplifies to:

$$\phi_{\mathrm{im},\alpha_{\mathrm{t}}^{\mathrm{S}}=0} = \frac{e}{4\pi\varepsilon^{\mathrm{L}}} \int_0^\infty \frac{\varepsilon^{\mathrm{L}} - \varepsilon^{\mathrm{G}}}{\varepsilon^{\mathrm{L}} + \varepsilon^{\mathrm{G}}} \mathrm{e}^{-\xi(z+z_{\mathrm{o}})} \mathrm{J}_0(\xi r) \mathrm{d}\xi = \frac{e}{4\pi\varepsilon^{\mathrm{L}}} \frac{\varepsilon^{\mathrm{L}} - \varepsilon^{\mathrm{G}}}{\varepsilon^{\mathrm{L}} + \varepsilon^{\mathrm{G}}} \frac{1}{\sqrt{r^2 + (z+z_{\mathrm{o}})^2}} ,$$

i.e. the classical image potential eq. (2) is restored. The first correction to it can be obtained by expanding the function under the integral in eq. (85) with respect to $\alpha_{\mathrm{t}}^{\mathrm{S}}$:

$$\phi_{\mathrm{im}} - \phi_{\mathrm{im},\alpha_{\mathrm{t}}^{\mathrm{S}}=0} \approx \frac{e}{2\pi} \int_0^\infty - \frac{\alpha_{\mathrm{t}}^{\mathrm{S}} \xi \mathrm{e}^{-\xi(z+z_{\mathrm{o}})} \mathrm{J}_0(\xi r) \mathrm{d}\xi}{\left(\varepsilon^{\mathrm{L}} + \varepsilon^{\mathrm{G}}\right)^2} = - \frac{e}{2\pi} \frac{\alpha_{\mathrm{t}}^{\mathrm{S}}}{\left(\varepsilon^{\mathrm{L}} + \varepsilon^{\mathrm{G}}\right)^2} \frac{z+z_{\mathrm{o}}}{\left[r^2 + (z+z_{\mathrm{o}})^2\right]^{3/2}} .$$

Therefore, at a surface that is not the dielectric one, rather than the classical eq. (2), the image charge potential involves another term:

$$\phi_{e,\mathrm{im}} = \frac{e_{\mathrm{im}}}{4\pi\varepsilon^{\mathrm{L}}} \frac{1}{|\boldsymbol{r} - \boldsymbol{r}_{\mathrm{im}}|} - \frac{e}{2\pi} \frac{\alpha_{\mathrm{t}}^{\mathrm{S}}}{\left(\varepsilon^{\mathrm{L}} + \varepsilon^{\mathrm{G}}\right)^2} \frac{z+z_{\mathrm{o}}}{|\boldsymbol{r} - \boldsymbol{r}_{\mathrm{im}}|^3} . \tag{86}$$

Thus, a charge at a distance z_{o} from a tangentially polarizable surface has additional image energy compared to the classical expression:

$$u_{ee} = \frac{1}{2} e \phi_{e,\mathrm{im}}(\boldsymbol{r}_{\mathrm{o}}) = \frac{k_{\mathrm{im}}^{\mathrm{L}}}{2} \frac{e^2}{|2z_{\mathrm{o}}|} - \frac{\alpha_{\mathrm{t}}^{\mathrm{S}}}{4\pi\left(\varepsilon^{\mathrm{L}} + \varepsilon^{\mathrm{G}}\right)^2} \frac{e^2}{|2z_{\mathrm{o}}|^2} . \tag{87}$$

The correction to the image energy is $\sim\alpha_{\mathrm{t}}^{\mathrm{S}} e^2/z_{\mathrm{o}}^2$, i.e. this correction is as long-ranged as the u_{ep} interaction for ion-dipoles. A surface with positive tangential polarizability attracts the charged particles from both the L and the G phase; on the contrary, if $\alpha_{\mathrm{t}}^{\mathrm{S}} < 0$, the tangential polarizability results in extra repulsion of all charged particles in both directions.

3. Image field and field gradients

The image potential of a point dipole-quadrupole-octupole is given by eq. (10). Its negative gradient leads to the image field:

$$\boldsymbol{E}_{\text{im}} = -\frac{\boldsymbol{p}_{\text{im}}}{4\pi\varepsilon^{\text{L}}\left|\boldsymbol{r}-\boldsymbol{r}_{\text{im}}\right|^{3}} + 3\frac{\boldsymbol{p}_{\text{im}}\cdot\left(\boldsymbol{r}-\boldsymbol{r}_{\text{im}}\right)\left(\boldsymbol{r}-\boldsymbol{r}_{\text{im}}\right)}{4\pi\varepsilon^{\text{L}}\left|\boldsymbol{r}-\boldsymbol{r}_{\text{im}}\right|^{5}}$$
$$-\frac{6\boldsymbol{q}_{\text{im}}\cdot\left(\boldsymbol{r}-\boldsymbol{r}_{\text{im}}\right)}{8\pi\varepsilon^{\text{L}}\left|\boldsymbol{r}-\boldsymbol{r}_{\text{im}}\right|^{5}} + \frac{15\boldsymbol{q}_{\text{im}}:\left(\boldsymbol{r}-\boldsymbol{r}_{\text{im}}\right)\left(\boldsymbol{r}-\boldsymbol{r}_{\text{im}}\right)\left(\boldsymbol{r}-\boldsymbol{r}_{\text{im}}\right)}{8\pi\varepsilon^{\text{L}}\left|\boldsymbol{r}-\boldsymbol{r}_{\text{im}}\right|^{7}}$$
$$-\frac{15\boldsymbol{o}_{\text{im}}:\left(\boldsymbol{r}-\boldsymbol{r}_{\text{im}}\right)\left(\boldsymbol{r}-\boldsymbol{r}_{\text{im}}\right)}{8\pi\varepsilon^{\text{L}}\left|\boldsymbol{r}-\boldsymbol{r}_{\text{im}}\right|^{7}} + \frac{35\boldsymbol{o}_{\text{im}}\therefore\left(\boldsymbol{r}-\boldsymbol{r}_{\text{im}}\right)\left(\boldsymbol{r}-\boldsymbol{r}_{\text{im}}\right)\left(\boldsymbol{r}-\boldsymbol{r}_{\text{im}}\right)\left(\boldsymbol{r}-\boldsymbol{r}_{\text{im}}\right)}{8\pi\varepsilon^{\text{L}}\left|\boldsymbol{r}-\boldsymbol{r}_{\text{im}}\right|^{9}}. \tag{88}$$

The value of it at $\boldsymbol{r} = \boldsymbol{r}_{\text{o}}$ gives eq. (11).

The image field gradient is obtained as a gradient of eq. (88):

$$\nabla\boldsymbol{E}_{\text{im}} = +\frac{3\left(\boldsymbol{r}-\boldsymbol{r}_{\text{im}}\right)\boldsymbol{p}_{\text{im}} + 3\boldsymbol{p}_{\text{im}}\left(\boldsymbol{r}-\boldsymbol{r}_{\text{im}}\right) + 3\boldsymbol{p}_{\text{im}}\cdot\left(\boldsymbol{r}-\boldsymbol{r}_{\text{im}}\right)\mathbf{U}}{4\pi\varepsilon^{\text{L}}\left|\boldsymbol{r}-\boldsymbol{r}_{\text{im}}\right|^{5}}$$
$$-\frac{15\boldsymbol{p}_{\text{im}}\cdot\left(\boldsymbol{r}-\boldsymbol{r}_{\text{im}}\right)\left(\boldsymbol{r}-\boldsymbol{r}_{\text{im}}\right)\left(\boldsymbol{r}-\boldsymbol{r}_{\text{im}}\right)}{4\pi\varepsilon^{\text{L}}\left|\boldsymbol{r}-\boldsymbol{r}_{\text{im}}\right|^{7}}$$
$$-\frac{6\boldsymbol{q}_{\text{im}}}{8\pi\varepsilon^{\text{L}}\left|\boldsymbol{r}-\boldsymbol{r}_{\text{im}}\right|^{5}}$$
$$+\frac{30\left(\boldsymbol{r}-\boldsymbol{r}_{\text{im}}\right)\boldsymbol{q}_{\text{im}}\cdot\left(\boldsymbol{r}-\boldsymbol{r}_{\text{im}}\right) + 30\boldsymbol{q}_{\text{im}}\cdot\left(\boldsymbol{r}-\boldsymbol{r}_{\text{im}}\right)\left(\boldsymbol{r}-\boldsymbol{r}_{\text{im}}\right) + 15\boldsymbol{q}_{\text{im}}:\left(\boldsymbol{r}-\boldsymbol{r}_{\text{im}}\right)\left(\boldsymbol{r}-\boldsymbol{r}_{\text{im}}\right)\mathbf{U}}{8\pi\varepsilon^{\text{L}}\left|\boldsymbol{r}-\boldsymbol{r}_{\text{im}}\right|^{7}}$$
$$-\frac{105\boldsymbol{q}_{\text{im}}:\left(\boldsymbol{r}-\boldsymbol{r}_{\text{im}}\right)\left(\boldsymbol{r}-\boldsymbol{r}_{\text{im}}\right)\left(\boldsymbol{r}-\boldsymbol{r}_{\text{im}}\right)\left(\boldsymbol{r}-\boldsymbol{r}_{\text{im}}\right)}{8\pi\varepsilon^{\text{L}}\left|\boldsymbol{r}-\boldsymbol{r}_{\text{im}}\right|^{9}}$$
$$-\frac{30\boldsymbol{o}_{\text{im}}\cdot\left(\boldsymbol{r}-\boldsymbol{r}_{\text{im}}\right)}{8\pi\varepsilon^{\text{L}}\left|\boldsymbol{r}-\boldsymbol{r}_{\text{im}}\right|^{7}}$$
$$+\frac{105\left(\boldsymbol{r}-\boldsymbol{r}_{\text{im}}\right)\boldsymbol{o}_{\text{im}}:\left(\boldsymbol{r}-\boldsymbol{r}_{\text{im}}\right)\left(\boldsymbol{r}-\boldsymbol{r}_{\text{im}}\right) + 105\boldsymbol{o}_{\text{im}}:\left(\boldsymbol{r}-\boldsymbol{r}_{\text{im}}\right)\left(\boldsymbol{r}-\boldsymbol{r}_{\text{im}}\right)\left(\boldsymbol{r}-\boldsymbol{r}_{\text{im}}\right)}{8\pi\varepsilon^{\text{L}}\left|\boldsymbol{r}-\boldsymbol{r}_{\text{im}}\right|^{9}}$$
$$+\frac{35\boldsymbol{o}_{\text{im}}\therefore\left(\boldsymbol{r}-\boldsymbol{r}_{\text{im}}\right)\left(\boldsymbol{r}-\boldsymbol{r}_{\text{im}}\right)\left(\boldsymbol{r}-\boldsymbol{r}_{\text{im}}\right)\mathbf{U}}{8\pi\varepsilon^{\text{L}}\left|\boldsymbol{r}-\boldsymbol{r}_{\text{im}}\right|^{9}}$$
$$-\frac{315\boldsymbol{o}_{\text{im}}\therefore\left(\boldsymbol{r}-\boldsymbol{r}_{\text{im}}\right)\left(\boldsymbol{r}-\boldsymbol{r}_{\text{im}}\right)\left(\boldsymbol{r}-\boldsymbol{r}_{\text{im}}\right)\left(\boldsymbol{r}-\boldsymbol{r}_{\text{im}}\right)\left(\boldsymbol{r}-\boldsymbol{r}_{\text{im}}\right)}{8\pi\varepsilon^{\text{L}}\left|\boldsymbol{r}-\boldsymbol{r}_{\text{im}}\right|^{11}}. \tag{89}$$

Its value at $\boldsymbol{r} = \boldsymbol{r}_\mathrm{o}$ is:

$$\begin{aligned}\nabla \boldsymbol{E}_\mathrm{im}(\boldsymbol{r}_\mathrm{o}) = &+\frac{3\left(\mathbf{e}_z \boldsymbol{p}_\mathrm{im} + \boldsymbol{p}_\mathrm{im}\mathbf{e}_z\right) + 3 p_{\mathrm{im},z}\left(\mathsf{U} - 5\mathbf{e}_z\mathbf{e}_z\right)}{4\pi\varepsilon^\mathrm{L}\left|2z_\mathrm{o}\right|^4} \\ &+\frac{-6\boldsymbol{q}_\mathrm{im} + 30\left(\mathbf{e}_z\boldsymbol{q}_\mathrm{im}\cdot\mathbf{e}_z + \boldsymbol{q}_\mathrm{im}\cdot\mathbf{e}_z\mathbf{e}_z\right) + 15 q_{\mathrm{im},zz}\left(\mathsf{U} - 7\mathbf{e}_z\mathbf{e}_z\right)}{8\pi\varepsilon^\mathrm{L}\left|2z_\mathrm{o}\right|^5} \\ &+\frac{-30\boldsymbol{o}_\mathrm{im}\cdot\mathbf{e}_z + 105\left(\mathbf{e}_z\boldsymbol{o}_\mathrm{im} : \mathbf{e}_z\mathbf{e}_z + \boldsymbol{o}_\mathrm{im} : \mathbf{e}_z\mathbf{e}_z\mathbf{e}_z\right) + 35 o_{\mathrm{im},zzz}\left(\mathsf{U} - 9\mathbf{e}_z\mathbf{e}_z\right)}{8\pi\varepsilon^\mathrm{L}\left|2z_\mathrm{o}\right|^6}.\end{aligned} \tag{90}$$

(continues on the next page)

Finaly, the image field double gradient is obtained from eq. (89):

$$
\begin{aligned}
\nabla\nabla\boldsymbol{E}_{\mathrm{im}} = &+\frac{3\mathbf{U}\boldsymbol{p}_{\mathrm{im}}+3\mathbf{e}_i\boldsymbol{p}_{\mathrm{im}}\mathbf{e}_i+3\boldsymbol{p}_{\mathrm{im}}\mathbf{U}}{4\pi\varepsilon^{\mathrm{L}}\left|\boldsymbol{r}-\boldsymbol{r}_{\mathrm{im}}\right|^5}\\
&+\frac{-15(\boldsymbol{r}-\boldsymbol{r}_{\mathrm{im}})(\boldsymbol{r}-\boldsymbol{r}_{\mathrm{im}})\boldsymbol{p}_{\mathrm{im}}-15(\boldsymbol{r}-\boldsymbol{r}_{\mathrm{im}})\boldsymbol{p}_{\mathrm{im}}(\boldsymbol{r}-\boldsymbol{r}_{\mathrm{im}})-15\boldsymbol{p}_{\mathrm{im}}(\boldsymbol{r}-\boldsymbol{r}_{\mathrm{im}})(\boldsymbol{r}-\boldsymbol{r}_{\mathrm{im}})}{4\pi\varepsilon^{\mathrm{L}}\left|\boldsymbol{r}-\boldsymbol{r}_{\mathrm{im}}\right|^7}\\
&+\frac{-15\boldsymbol{p}_{\mathrm{im}}\cdot(\boldsymbol{r}-\boldsymbol{r}_{\mathrm{im}})\left[(\boldsymbol{r}-\boldsymbol{r}_{\mathrm{im}})\mathbf{U}+\mathbf{e}_i(\boldsymbol{r}-\boldsymbol{r}_{\mathrm{im}})\mathbf{e}_i+\mathbf{U}(\boldsymbol{r}-\boldsymbol{r}_{\mathrm{im}})\right]}{4\pi\varepsilon^{\mathrm{L}}\left|\boldsymbol{r}-\boldsymbol{r}_{\mathrm{im}}\right|^7}\\
&+\frac{105\boldsymbol{p}_{\mathrm{im}}\cdot(\boldsymbol{r}-\boldsymbol{r}_{\mathrm{im}})(\boldsymbol{r}-\boldsymbol{r}_{\mathrm{im}})(\boldsymbol{r}-\boldsymbol{r}_{\mathrm{im}})(\boldsymbol{r}-\boldsymbol{r}_{\mathrm{im}})}{4\pi\varepsilon^{\mathrm{L}}\left|\boldsymbol{r}-\boldsymbol{r}_{\mathrm{im}}\right|^9}\\
&+\frac{30(\boldsymbol{r}-\boldsymbol{r}_{\mathrm{im}})\boldsymbol{q}_{\mathrm{im}}+30\mathbf{e}_i(\boldsymbol{r}-\boldsymbol{r}_{\mathrm{im}})q_{\mathrm{im},ij}\mathbf{e}_j+30\boldsymbol{q}_{\mathrm{im}}(\boldsymbol{r}-\boldsymbol{r}_{\mathrm{im}})}{8\pi\varepsilon^{\mathrm{L}}\left|\boldsymbol{r}-\boldsymbol{r}_{\mathrm{im}}\right|^7}\\
&+\frac{30\mathbf{U}\boldsymbol{q}_{\mathrm{im}}\cdot(\boldsymbol{r}-\boldsymbol{r}_{\mathrm{im}})+30\mathbf{e}_i\boldsymbol{q}_{\mathrm{im}}\cdot(\boldsymbol{r}-\boldsymbol{r}_{\mathrm{im}})\mathbf{e}_i+30\boldsymbol{q}_{\mathrm{im}}\cdot(\boldsymbol{r}-\boldsymbol{r}_{\mathrm{im}})\mathbf{U}}{8\pi\varepsilon^{\mathrm{L}}\left|\boldsymbol{r}-\boldsymbol{r}_{\mathrm{im}}\right|^7}\\
&+\frac{\begin{bmatrix}-210(\boldsymbol{r}-\boldsymbol{r}_{\mathrm{im}})(\boldsymbol{r}-\boldsymbol{r}_{\mathrm{im}})\boldsymbol{q}_{\mathrm{im}}\cdot(\boldsymbol{r}-\boldsymbol{r}_{\mathrm{im}})-210(\boldsymbol{r}-\boldsymbol{r}_{\mathrm{im}})\boldsymbol{q}_{\mathrm{im}}\cdot(\boldsymbol{r}-\boldsymbol{r}_{\mathrm{im}})(\boldsymbol{r}-\boldsymbol{r}_{\mathrm{im}})\\ -210\boldsymbol{q}_{\mathrm{im}}\cdot(\boldsymbol{r}-\boldsymbol{r}_{\mathrm{im}})(\boldsymbol{r}-\boldsymbol{r}_{\mathrm{im}})(\boldsymbol{r}-\boldsymbol{r}_{\mathrm{im}})\end{bmatrix}}{8\pi\varepsilon^{\mathrm{L}}\left|\boldsymbol{r}-\boldsymbol{r}_{\mathrm{im}}\right|^9}\\
&+\frac{-105\boldsymbol{q}_{\mathrm{im}}:(\boldsymbol{r}-\boldsymbol{r}_{\mathrm{im}})(\boldsymbol{r}-\boldsymbol{r}_{\mathrm{im}})\left[(\boldsymbol{r}-\boldsymbol{r}_{\mathrm{im}})\mathbf{U}+\mathbf{e}_i(\boldsymbol{r}-\boldsymbol{r}_{\mathrm{im}})\mathbf{e}_i+\mathbf{U}(\boldsymbol{r}-\boldsymbol{r}_{\mathrm{im}})\right]}{8\pi\varepsilon^{\mathrm{L}}\left|\boldsymbol{r}-\boldsymbol{r}_{\mathrm{im}}\right|^9}\\
&+\frac{945\boldsymbol{q}_{\mathrm{im}}:(\boldsymbol{r}-\boldsymbol{r}_{\mathrm{im}})(\boldsymbol{r}-\boldsymbol{r}_{\mathrm{im}})(\boldsymbol{r}-\boldsymbol{r}_{\mathrm{im}})(\boldsymbol{r}-\boldsymbol{r}_{\mathrm{im}})(\boldsymbol{r}-\boldsymbol{r}_{\mathrm{im}})}{8\pi\varepsilon^{\mathrm{L}}\left|\boldsymbol{r}-\boldsymbol{r}_{\mathrm{im}}\right|^{11}}\\
&+\frac{-30\boldsymbol{o}_{\mathrm{im}}}{8\pi\varepsilon^{\mathrm{L}}\left|\boldsymbol{r}-\boldsymbol{r}_{\mathrm{im}}\right|^7}\\
&+\frac{210(\boldsymbol{r}-\boldsymbol{r}_{\mathrm{im}})\boldsymbol{o}_{\mathrm{im}}\cdot(\boldsymbol{r}-\boldsymbol{r}_{\mathrm{im}})+210o_{\mathrm{im},ijm}(r-r_{\mathrm{im}})_m\mathbf{e}_i(\boldsymbol{r}-\boldsymbol{r}_{\mathrm{im}})\mathbf{e}_j+210\boldsymbol{o}_{\mathrm{im}}\cdot(\boldsymbol{r}-\boldsymbol{r}_{\mathrm{im}})(\boldsymbol{r}-\boldsymbol{r}_{\mathrm{im}})}{8\pi\varepsilon^{\mathrm{L}}\left|\boldsymbol{r}-\boldsymbol{r}_{\mathrm{im}}\right|^9}\\
&+\frac{105\mathbf{U}\boldsymbol{o}_{\mathrm{im}}:(\boldsymbol{r}-\boldsymbol{r}_{\mathrm{im}})(\boldsymbol{r}-\boldsymbol{r}_{\mathrm{im}})+105\mathbf{e}_i\boldsymbol{o}_{\mathrm{im}}:(\boldsymbol{r}-\boldsymbol{r}_{\mathrm{im}})(\boldsymbol{r}-\boldsymbol{r}_{\mathrm{im}})\mathbf{e}_i+105\boldsymbol{o}_{\mathrm{im}}:(\boldsymbol{r}-\boldsymbol{r}_{\mathrm{im}})(\boldsymbol{r}-\boldsymbol{r}_{\mathrm{im}})\mathbf{U}}{8\pi\varepsilon^{\mathrm{L}}\left|\boldsymbol{r}-\boldsymbol{r}_{\mathrm{im}}\right|^9}\\
&+\frac{\begin{bmatrix}-945(\boldsymbol{r}-\boldsymbol{r}_{\mathrm{im}})(\boldsymbol{r}-\boldsymbol{r}_{\mathrm{im}})\boldsymbol{o}_{\mathrm{im}}:(\boldsymbol{r}-\boldsymbol{r}_{\mathrm{im}})(\boldsymbol{r}-\boldsymbol{r}_{\mathrm{im}})-945(\boldsymbol{r}-\boldsymbol{r}_{\mathrm{im}})\boldsymbol{o}_{\mathrm{im}}:(\boldsymbol{r}-\boldsymbol{r}_{\mathrm{im}})(\boldsymbol{r}-\boldsymbol{r}_{\mathrm{im}})(\boldsymbol{r}-\boldsymbol{r}_{\mathrm{im}})\\ -945\boldsymbol{o}_{\mathrm{im}}:(\boldsymbol{r}-\boldsymbol{r}_{\mathrm{im}})(\boldsymbol{r}-\boldsymbol{r}_{\mathrm{im}})(\boldsymbol{r}-\boldsymbol{r}_{\mathrm{im}})(\boldsymbol{r}-\boldsymbol{r}_{\mathrm{im}})\end{bmatrix}}{8\pi\varepsilon^{\mathrm{L}}\left|\boldsymbol{r}-\boldsymbol{r}_{\mathrm{im}}\right|^{11}}\\
&+\frac{-315\boldsymbol{o}_{\mathrm{im}}\therefore(\boldsymbol{r}-\boldsymbol{r}_{\mathrm{im}})(\boldsymbol{r}-\boldsymbol{r}_{\mathrm{im}})(\boldsymbol{r}-\boldsymbol{r}_{\mathrm{im}})\left[(\boldsymbol{r}-\boldsymbol{r}_{\mathrm{im}})\mathbf{U}+\mathbf{e}_i(\boldsymbol{r}-\boldsymbol{r}_{\mathrm{im}})\mathbf{e}_i+\mathbf{U}(\boldsymbol{r}-\boldsymbol{r}_{\mathrm{im}})\right]}{8\pi\varepsilon^{\mathrm{L}}\left|\boldsymbol{r}-\boldsymbol{r}_{\mathrm{im}}\right|^{11}}\\
&+\frac{3465\boldsymbol{o}_{\mathrm{im}}\therefore(\boldsymbol{r}-\boldsymbol{r}_{\mathrm{im}})(\boldsymbol{r}-\boldsymbol{r}_{\mathrm{im}})(\boldsymbol{r}-\boldsymbol{r}_{\mathrm{im}})(\boldsymbol{r}-\boldsymbol{r}_{\mathrm{im}})(\boldsymbol{r}-\boldsymbol{r}_{\mathrm{im}})(\boldsymbol{r}-\boldsymbol{r}_{\mathrm{im}})}{8\pi\varepsilon^{\mathrm{L}}\left|\boldsymbol{r}-\boldsymbol{r}_{\mathrm{im}}\right|^{13}}.
\end{aligned}
$$

At $\boldsymbol{r} = \boldsymbol{r}_\mathrm{o}$, the image field double gradient simplifies to:

$$\nabla\nabla \boldsymbol{E}_\mathrm{im}(\boldsymbol{r}_\mathrm{o}) = +\frac{\begin{bmatrix} 3\mathbf{U}\boldsymbol{p}_\mathrm{im} + 3\mathbf{e}_i\boldsymbol{p}_\mathrm{im}\mathbf{e}_i + 3\boldsymbol{p}_\mathrm{im}\mathbf{U} \\ -15\mathbf{e}_z\mathbf{e}_z\boldsymbol{p}_\mathrm{im} - 15\mathbf{e}_z\boldsymbol{p}_\mathrm{im}\mathbf{e}_z - 15\boldsymbol{p}_\mathrm{im}\mathbf{e}_z\mathbf{e}_z \\ -15p_{\mathrm{im},z}\left(\mathbf{e}_z\mathbf{U} + \mathbf{e}_i\mathbf{e}_z\mathbf{e}_i + \mathbf{U}\mathbf{e}_z - 7\mathbf{e}_z\mathbf{e}_z\mathbf{e}_z\right) \end{bmatrix}}{4\pi\varepsilon^\mathrm{L}\left|2z_\mathrm{o}\right|^5}$$

$$+\frac{\begin{bmatrix} 30\mathbf{e}_z\boldsymbol{q}_\mathrm{im} + 30\mathbf{e}_i\mathbf{e}_z q_{\mathrm{im},ij}\mathbf{e}_j + 30\boldsymbol{q}_\mathrm{im}\mathbf{e}_z \\ +30\mathbf{U}\boldsymbol{q}_\mathrm{im}\cdot\mathbf{e}_z + 30\mathbf{e}_i\boldsymbol{q}_\mathrm{im}\cdot\mathbf{e}_z\mathbf{e}_i + 30\boldsymbol{q}_\mathrm{im}\cdot\mathbf{e}_z\mathbf{U} \\ -210\mathbf{e}_z\mathbf{e}_z\boldsymbol{q}_\mathrm{im}\cdot\mathbf{e}_z - 210\mathbf{e}_z\boldsymbol{q}_\mathrm{im}\cdot\mathbf{e}_z\mathbf{e}_z - 210\boldsymbol{q}_\mathrm{im}\cdot\mathbf{e}_z\mathbf{e}_z\mathbf{e}_z \\ -105q_{\mathrm{im},zz}\left(\mathbf{e}_z\mathbf{U} + \mathbf{e}_i\mathbf{e}_z\mathbf{e}_i + \mathbf{U}\mathbf{e}_z - 9\mathbf{e}_z\mathbf{e}_z\mathbf{e}_z\right) \end{bmatrix}}{8\pi\varepsilon^\mathrm{L}\left|2z_\mathrm{o}\right|^6}$$

$$+\frac{\begin{bmatrix} -30\boldsymbol{o}_\mathrm{im} \\ +210\mathbf{e}_z\boldsymbol{o}_\mathrm{im}\cdot\mathbf{e}_z + 210o_{\mathrm{im},ijz}\mathbf{e}_i\mathbf{e}_z\mathbf{e}_j + 210\boldsymbol{o}_\mathrm{im}\cdot\mathbf{e}_z\mathbf{e}_z \\ +105\mathbf{U}\boldsymbol{o}_\mathrm{im}:\mathbf{e}_z\mathbf{e}_z + 105\mathbf{e}_i\boldsymbol{o}_\mathrm{im}:\mathbf{e}_z\mathbf{e}_z\mathbf{e}_i + 105\boldsymbol{o}_\mathrm{im}:\mathbf{e}_z\mathbf{e}_z\mathbf{U} \\ -945\mathbf{e}_z\mathbf{e}_z\boldsymbol{o}_\mathrm{im}:\mathbf{e}_z\mathbf{e}_z - 945\mathbf{e}_z\boldsymbol{o}_\mathrm{im}:\mathbf{e}_z\mathbf{e}_z\mathbf{e}_z - 945\boldsymbol{o}_\mathrm{im}:\mathbf{e}_z\mathbf{e}_z\mathbf{e}_z\mathbf{e}_z \\ -315o_{\mathrm{im},zzz}\left(\mathbf{e}_z\mathbf{U} + \mathbf{e}_i\mathbf{e}_z\mathbf{e}_i + \mathbf{U}\mathbf{e}_z - 11\mathbf{e}_z\mathbf{e}_z\mathbf{e}_z\right) \end{bmatrix}}{8\pi\varepsilon^\mathrm{L}\left|2z_\mathrm{o}\right|^7}. \tag{91}$$

We used the formulae:

$$\nabla\left|\boldsymbol{r} - \boldsymbol{r}_\mathrm{im}\right|^n = n\left|\boldsymbol{r} - \boldsymbol{r}_\mathrm{im}\right|^{n-2}\left(\boldsymbol{r} - \boldsymbol{r}_\mathrm{im}\right) \quad \text{and}$$

$$\boldsymbol{r}_\mathrm{o} - \boldsymbol{r}_\mathrm{im} = (0, 0, 2z_\mathrm{o}) = 2z_\mathrm{o}\mathbf{e}_z\,.$$

4. Conversion of multipole moments upon changing the origin

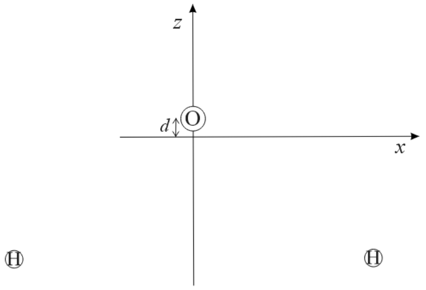

Figure S 1. Coordinate system used for water by Batista et al.

The dipole moment reported by Batista et al.[39] refers to origin placed in the centre of mass and orientation of the water molecule with all nuclei in the xz plane, oxygen pointing up in direction $\mathbf{e}_z$, see Figure S 1. Therefore, the dipole moment is:

$$\boldsymbol{p}_m = \int \rho_{\text{molecule}} \boldsymbol{r} \mathrm{d}V = (0,0,-p_0) .$$

The oxygen nucleus in the coordinate system of Batista et al. is located at $\boldsymbol{r}_\mathrm{O} = (0,0,d)$ where d = 0.06586 Å. Changing the origin from $\boldsymbol{r}_m$ = (0,0,0) to $\boldsymbol{r}_\mathrm{O}$ = (0,0,d) does not alter the dipole of a neutral particle:

$$\boldsymbol{p}_\mathrm{O} = \int \rho_{\text{molecule}} \left(\boldsymbol{r} - \boldsymbol{r}_\mathrm{O}\right) \mathrm{d}V = \boldsymbol{p}_m ,$$

because $e = \int \rho \mathrm{d}V = 0$. However, the quadrupole moment $\boldsymbol{q}_\mathrm{O}$ is different from $\boldsymbol{q}_m$:

$$\boldsymbol{q}_{\text{O-Tr}} = \int \rho_{\text{molecule}} \left(\boldsymbol{r} - \boldsymbol{r}_\mathrm{O}\right)\left(\boldsymbol{r} - \boldsymbol{r}_\mathrm{O}\right) \mathrm{d}V = \boldsymbol{q}_m - \boldsymbol{r}_\mathrm{O}\boldsymbol{p}_m - \boldsymbol{p}_m\boldsymbol{r}_\mathrm{O} = \boldsymbol{q}_m + 2p_0 d \mathbf{e}_z \mathbf{e}_z .$$

According to this formula, only the zz component acquires a new value, namely:

$$q_{\text{O-Tr},zz} = q_{m,zz} + 2p_0 d .$$

The moment $\boldsymbol{q}_{\text{O-Tr}}$, however, has a nonzero trace: $\mathrm{Tr}\boldsymbol{q}_{\text{O-Tr}} = +2p_0 d$. To construct the new traceless quadrupole moment, we subtract $\mathrm{Tr}\boldsymbol{q}_{\text{O-Tr}}/3\times\mathbf{U}$ from $\boldsymbol{q}_{\text{O-Tr}}$:

$$\boldsymbol{q}_\mathrm{O} = \boldsymbol{q}_{\text{O-Tr}} - \frac{2}{3} p_0 d \mathbf{U}, \text{ i.e.}$$

$$q_{\mathrm{O},xx} = q_{m,xx} - \frac{2}{3} p_0 d , \quad q_{\mathrm{O},yy} = q_{m,yy} - \frac{2}{3} p_0 d , \quad q_{\mathrm{O},zz} = q_{m,zz} + \frac{4}{3} p_0 d .$$

We convert the octupole moment similarly. We expand the brackets in the definition:

$$\boldsymbol{o}_{\text{O-Tr}} = \int \rho_{\text{molecule}} \left(\boldsymbol{r} - \boldsymbol{r}_\mathrm{O}\right)\left(\boldsymbol{r} - \boldsymbol{r}_\mathrm{O}\right)\left(\boldsymbol{r} - \boldsymbol{r}_\mathrm{O}\right) \mathrm{d}V ,$$

which leads to:

$$o_{\text{O-Tr},ijk} = o_{m,ijk} + p_{m,i} r_{\mathrm{O},j} r_{\mathrm{O},k} + r_{\mathrm{O},i} p_{m,j} r_{\mathrm{O},k} + r_{\mathrm{O},i} r_{\mathrm{O},j} p_{m,k} - q_{m,ij} r_{\mathrm{O},k} - q_{m,ik} r_{\mathrm{O},j} - q_{m,jk} r_{\mathrm{O},i} .$$

This corresponds to the following component values:

$$o_{\text{O-Tr},xxz} = o_{m,xxz} - q_{m,xx} d ; \quad o_{\text{O-Tr},yyz} = o_{m,yyz} - q_{m,yy} d ;$$

$$o_{\text{O-Tr},zzz} = o_{m,zzz} - 3p_0 d^2 - 3q_{m,zz} d . \qquad (92)$$

This octupole has one nonzero 'trace':

$$o_{\text{O-Tr},xxz} + o_{\text{O-Tr},yyz} + o_{\text{O-Tr},zzz} = -3p_0 d^2 - 2q_{m,zz} d .$$

We subtract a third of it from the components (92) to obtain the final formulae:

$$o_{\mathrm{O},xxz} = o_{m,xxz} + p_0 d^2 + \frac{2}{3} q_{m,zz} d - q_{m,xx} d \, ;$$

$$o_{\mathrm{O},yyz} = o_{m,yyz} + p_0 d^2 + \frac{2}{3} q_{m,zz} d - q_{m,yy} d \, ;$$

$$o_{\mathrm{O},zzz} = o_{m,zzz} - 2 p_0 d^2 - \frac{7}{3} q_{m,zz} d \, . \tag{93}$$

Batista et al.[39] use also other definitions for the quadrupole and octupole moments that differ from ours by factor 3/2 in the quadrupole and 5/2 in the octupole. All values in Table 2 were converted to our definition of choice.

5. Explicit expressions for the orientation distribution of water

For water, eq. (27),(28)&(29) read:

$$U_{pp}^{\mathrm{L}} = p_0^2\left(\frac{2\cos^2\theta}{1+2k_{\mathrm{im}}^{\mathrm{L}}\alpha_p/|2z_{\mathrm{o}}|^3}+\frac{\sin^2\theta}{1+k_{\mathrm{im}}^{\mathrm{L}}\alpha_p/|2z_{\mathrm{o}}|^3}\right);$$

$$U_{pp}^{\mathrm{G}} = p_0^2\left(\frac{2\cos^2\theta}{1-2k_{\mathrm{im}}^{\mathrm{G}}\alpha_p/|2z_{\mathrm{o}}|^3}+\frac{\sin^2\theta}{1-k_{\mathrm{im}}^{\mathrm{G}}\alpha_p/|2z_{\mathrm{o}}|^3}\right);$$

$$U_{pp,\mathrm{cav}}^{\mathrm{L}} = \frac{Y_E^2}{\left(1-\alpha_p X_p\right)^2}\left(\frac{2\cos^2\theta}{1+2k_{\mathrm{im}}^{\mathrm{L}}\alpha_{p,\mathrm{cav}}/|2z_{\mathrm{o}}|^3}+\frac{\sin^2\theta}{1+k_{\mathrm{im}}^{\mathrm{L}}\alpha_{p,\mathrm{cav}}/|2z_{\mathrm{o}}|^3}\right);$$

$$U_{pp,\mathrm{cav}}^{\mathrm{G}} = \frac{Y_E^2}{\left(1-\alpha_p X_p\right)^2}\left(\frac{2\cos^2\theta}{1-2k_{\mathrm{im}}^{\mathrm{G}}\alpha_{p,\mathrm{cav}}/|2z_{\mathrm{o}}|^3}+\frac{\sin^2\theta}{1-k_{\mathrm{im}}^{\mathrm{G}}\alpha_{p,\mathrm{cav}}/|2z_{\mathrm{o}}|^3}\right). \tag{94}$$

For water, eq. (15)-(17)&(31) read:

$$U_{pq} = 3p_0\cos\theta\left[3\left(-1+\frac{1}{2}\sin^2\theta\right)q_{zz}+\frac{1}{2}\cos(2\varphi)\sin^2\theta\left(q_{xx}-q_{yy}\right)\right];$$

$$U_{pq,\mathrm{cav}} = \frac{3Y_E Y_{\nabla E} p_0}{\left(1-\alpha_p X_p\right)\left(1-\alpha_q X_q\right)}\cos\theta\left[3\left(-1+\frac{1}{2}\sin^2\theta\right)q_{zz}+\frac{1}{2}\cos(2\varphi)\sin^2\theta\left(q_{xx}-q_{yy}\right)\right]; \tag{95}$$

$$\begin{aligned} U_{qq} = {} & \frac{27}{4}\left(2\cos^2\theta+\frac{3}{4}\sin^4\theta\right)q_{zz}^2 \\ & +\frac{9}{4}\cos(2\varphi)\left(1+\frac{3}{2}\sin^2\theta\right)\sin^2\theta\left(q_{xx}-q_{yy}\right)q_{zz} \\ & +\frac{3}{4}\left(1+3\sin^2\theta+\frac{3}{4}\cos^2(2\varphi)\sin^4\theta\right)\left(q_{xx}-q_{yy}\right)^2. \end{aligned} \tag{96}$$

$$U_{po} = 5\left(4-8\sin^2\theta+\frac{5}{2}\sin^4\theta\right)p_0 o_{zzz}-15\cos(2\varphi)\left(1-\frac{1}{2}\sin^2\theta\right)\sin^2\theta\, p_0\left(o_{xxz}-o_{yyz}\right). \tag{97}$$

At $\theta = 90°$ (water dipole standing parallel to the surface), eq. (95)-(97) simplify to:

$$U_{pp}^{\mathrm{L}} = \frac{p_0^2}{1+k_{\mathrm{im}}^{\mathrm{L}}\alpha_p/|2z_{\mathrm{o}}|^3} = const;\quad U_{pp}^{\mathrm{G}} = \frac{p_0^2}{1-k_{\mathrm{im}}^{\mathrm{G}}\alpha_p/|2z_{\mathrm{o}}|^3} = const;$$

$$U_{pq} = 0;$$

$$U_{qq}+U_{po} = const+\frac{15}{2}\left[\frac{3}{4}\left(q_{xx}-q_{yy}\right)q_{zz}-p_0\left(o_{xxz}-o_{yyz}\right)\right]\cos 2\varphi+\frac{9}{16}\left(q_{xx}-q_{yy}\right)^2\cos^2 2\varphi. \tag{98}$$

6. Structure of the surface layer for polarizable molecules

If the molecule is polarizable, the explicit form of the integral is:

$$\bar{p}_z^{\mathrm{L}} = \int_0^{\pi}\int_0^{2\pi}\int_0^{2\pi} p_z \rho_{\varphi\psi\theta} \sin\theta \mathrm{d}\varphi \mathrm{d}\psi \mathrm{d}\theta = \frac{k_{\mathrm{im}}^{\mathrm{L}}}{1+2k_{\mathrm{im}}^{\mathrm{L}}\alpha_p / \left|2z_{\mathrm{o}}^3\right|} \frac{3q_{\mathrm{n},zz} p_0^2}{40kT} \frac{1}{z_{\mathrm{o}}^4}, \tag{99}$$

where p_z is given by eq. (22) and $\rho_{\varphi\psi\theta}$ – by the linearized eq. (41), but this time with energy from eq. (26) with polarization. Instead of eq. (42), in this case, the average energy is:

$$\bar{u}^{\mathrm{L}} = \frac{2}{3} k_{\mathrm{im}}^{\mathrm{L}} \frac{1+\frac{3}{2}k_{\mathrm{im}}^{\mathrm{L}}\alpha_p / \left|2z_{\mathrm{o}}\right|^3}{\left(1+2k_{\mathrm{im}}^{\mathrm{L}}\alpha_p / \left|2z_{\mathrm{o}}\right|^3\right)\left(1+k_{\mathrm{im}}^{\mathrm{L}}\alpha_p / \left|2z_{\mathrm{o}}\right|^3\right)} \frac{p_0^2}{\left|2z_{\mathrm{o}}\right|^3} + \frac{12}{5} k_{\mathrm{im}}^{\mathrm{L}} \frac{\boldsymbol{q}:\boldsymbol{q}}{\left|2z_{\mathrm{o}}\right|^5}. \tag{100}$$

The average squares of the dipole moment in the liquid phase are given by:

$$\frac{\overline{p_{0z}^2}}{p_0^2} = \frac{1}{3} - \frac{8k_{\mathrm{im}}^{\mathrm{L}}}{5kT}\left[\frac{p_0^2}{36\left(1+2k_{\mathrm{im}}^{\mathrm{L}}\alpha_p / \left|2z_{\mathrm{o}}\right|^3\right)\left(1+k_{\mathrm{im}}^{\mathrm{L}}\alpha_p / \left|2z_{\mathrm{o}}\right|^3\right)\left|2z_{\mathrm{o}}\right|^3} + \frac{3\left(q_{\mathrm{n},xz}^2+q_{\mathrm{n},yz}^2+q_{\mathrm{n},zz}^2\right) - \boldsymbol{q}:\boldsymbol{q} - \frac{55}{12}p_0 o_{\mathrm{n},zzz}}{7\left|2z_{\mathrm{o}}\right|^5}\right];$$

$$\frac{\overline{p_{0x}^2+p_{0y}^2}}{p_0^2} = \frac{2}{3} + \frac{8k_{\mathrm{im}}^{\mathrm{L}}}{5kT}\left[\frac{p_0^2}{36\left(1+2k_{\mathrm{im}}^{\mathrm{L}}\alpha_p / \left|2z_{\mathrm{o}}\right|^3\right)\left(1+k_{\mathrm{im}}^{\mathrm{L}}\alpha_p / \left|2z_{\mathrm{o}}\right|^3\right)\left|2z_{\mathrm{o}}\right|^3} + \frac{3\left(q_{\mathrm{n},xz}^2+q_{\mathrm{n},yz}^2+q_{\mathrm{n},zz}^2\right) - \boldsymbol{q}:\boldsymbol{q} - \frac{55}{12}p_0 o_{\mathrm{n},zzz}}{7\left|2z_{\mathrm{o}}\right|^5}\right];$$

$$\overline{p_z^2} = \frac{\overline{p_{0z}^2}}{\left(1+2k_{\mathrm{im}}^{\mathrm{L}}\alpha_p / \left|2z_{\mathrm{o}}\right|^3\right)^2}; \qquad \overline{p_x^2+p_y^2} = \frac{\overline{p_{0x}^2+p_{0y}^2}}{\left(1+k_{\mathrm{im}}^{\mathrm{L}}\alpha_p / \left|2z_{\mathrm{o}}\right|^3\right)^2}. \tag{101}$$

The average squares of the dipole moment in the gas phase are:

$$\frac{\overline{p_{0z}^2}}{p_0^2} = \frac{1}{3} + \frac{8k_{\mathrm{im}}^{\mathrm{G}}}{5kT}\left[\frac{p_0^2}{36\left(1-2k_{\mathrm{im}}^{\mathrm{G}}\alpha_p / \left|2z_{\mathrm{o}}\right|^3\right)\left(1-k_{\mathrm{im}}^{\mathrm{G}}\alpha_p / \left|2z_{\mathrm{o}}\right|^3\right)\left|2z_{\mathrm{o}}\right|^3} + \frac{3\left(q_{\mathrm{n},xz}^2+q_{\mathrm{n},yz}^2+q_{\mathrm{n},zz}^2\right) - \boldsymbol{q}:\boldsymbol{q} - \frac{55}{12}p_0 o_{\mathrm{n},zzz}}{7\left|2z_{\mathrm{o}}\right|^5}\right];$$

$$\frac{\overline{p_{0x}^2+p_{0y}^2}}{p_0^2} = \frac{2}{3} - \frac{8k_{\mathrm{im}}^{\mathrm{G}}}{5kT}\left[\frac{p_0^2}{36\left(1-2k_{\mathrm{im}}^{\mathrm{G}}\alpha_p / \left|2z_{\mathrm{o}}\right|^3\right)\left(1-k_{\mathrm{im}}^{\mathrm{G}}\alpha_p / \left|2z_{\mathrm{o}}\right|^3\right)\left|2z_{\mathrm{o}}\right|^3} + \frac{3\left(q_{\mathrm{n},xz}^2+q_{\mathrm{n},yz}^2+q_{\mathrm{n},zz}^2\right) - \boldsymbol{q}:\boldsymbol{q} - \frac{55}{12}p_0 o_{\mathrm{n},zzz}}{7\left|2z_{\mathrm{o}}\right|^5}\right].$$

$$\overline{p_z^2} = \frac{\overline{p_{0z}^2}}{\left(1-2k_{\rm im}^{\rm G}\alpha_p/|2z_{\rm o}|^3\right)^2}; \qquad \overline{p_x^2+p_y^2} = \frac{\overline{p_{0x}^2+p_{0y}^2}}{\left(1-k_{\rm im}^{\rm G}\alpha_p/|2z_{\rm o}|^3\right)^2}. \qquad (102)$$

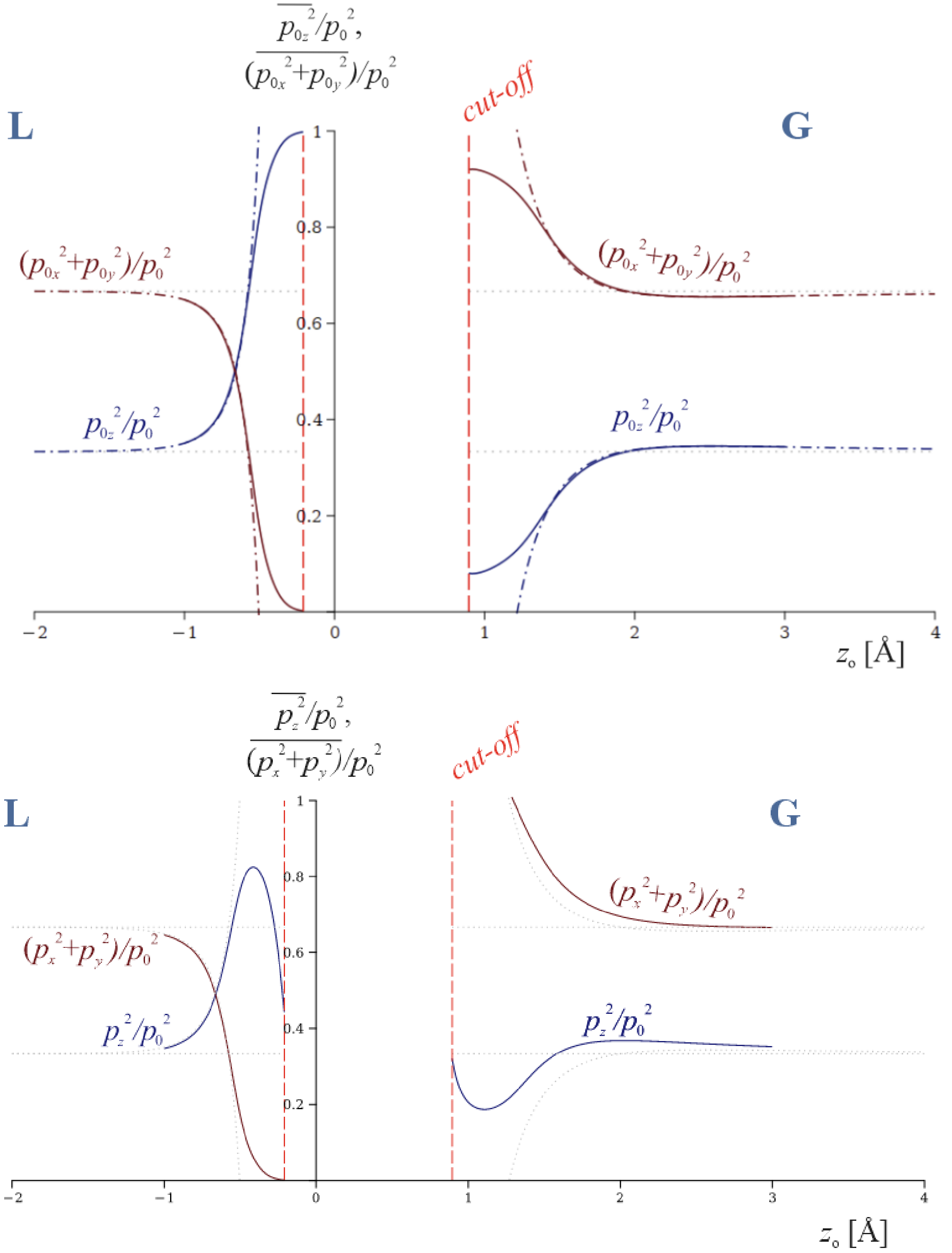

Figure S 2. Mean square values of the normal and the tangential dipole moment of a molecule near the surface (compare to Figure 5).

According to Figure 4, Figure 5 and Figure S 2, the structure of the adsorbed layer of dipoles appears to be multilayered:

Liquid phase, z_o = -4…-1.5 Å below the dielectric surface (parallel L-*pp* layer). Here, molecules are preferentially orientated tangentially to the surface due to the $\boldsymbol{pp}/z_o^3$ image force; this layer carries normal dipole moment only through the DDL effect.

Liquid phase, z_o = -1.5…-0.5 Å (normal L-*qq-po* layer). Here, the molecules are preferentially orientated normally to the surface, mostly due to the $\boldsymbol{qq}/z_o^5$ and $\boldsymbol{po}/z_o^5$ forces; the $\boldsymbol{pq}/z_o^4$ interaction causes considerable orientation of the dipole moment towards the liquid phase. Also notable is a significant preference for $\theta = 90°$, $\varphi = 0°$ (***p*** parallel to the surface, all atoms in the *xy* plane – hydrogens parallel to the surface).

Transition layer, z_o = -0.5…+1 Å. Here, the molecules sharply change orientation from mostly normal to the surface to mostly tangential to the surface. There is significant effect from the polarization and a significant preference for dipole moment in direction of the liquid phase. This layer carries most of the adsorbed dipole moment.

Gas phase, z_o = +1…+2 Å (parallel G-*qq-po* layer). Here, the molecules are orientated preferentially in the *xy* plane by the $\boldsymbol{qq}/z_o^5$ and $\boldsymbol{po}/z_o^5$ forces but, nevertheless, are carrying a considerable nonvanishing average normal dipole in direction towards the liquid phase due to $\boldsymbol{pq}/z_o^4$, magnified by a significant polarization. Also notable is a preference for $\theta = 90°$, $\varphi = 90°$ configuration (i.e. ***p*** parallel to the surface, all atoms in the *xz* plane – hydrogens up).

Gas phase, z_o = +2…+4 Å (normal G-*pp* layer). Here, molecules are preferentially orientated normally to the surface due to the $\boldsymbol{pp}/z_o^3$ image force.

The multipole-in-a-cavity model produces a different structure on the liquid side of the surface layer: in particular, the population of tangentially orientated molecules remains higher even close to the adsorption layer. Since the cavity model underestimates the higher-order multipoles (as we correct for the cavity effects only to the ***pp*** and the ***pq*** terms), this result is probably an overestimation. By contrast, the point multipole model greatly overestimates the predominance of normally orientated dipoles in this layer.

7. Effect of the cavity field and the reaction field on the image force

7.1. *pp* image force in the liquid phase

The image potential is produced by the total dipole moment $\boldsymbol{p}_{\text{tot}}$ of the whole entity, molecule plus cavity. A molecule and its cavity carry a total dipole moment that is the sum of their Onsager external unperturbed 'reaction' dipole $\boldsymbol{p}_{\text{ext}}$ and the dipole $\boldsymbol{p}_{\text{ext},E}$ induced by $\boldsymbol{E}_{\text{im}}$. From ref. [36]:

$$\boldsymbol{p}_{\text{ext}} = Y_E \boldsymbol{p}\,, \quad Y_E = \frac{3 f_E \varepsilon}{2\varepsilon + f_p \varepsilon_0}\,;$$

$$\boldsymbol{p}_{\text{ext},E} = -\frac{4\pi}{3} R_{\text{cav}}^3 Y_E \left(\varepsilon - \varepsilon_0\right) \boldsymbol{E}_{\text{im}}\,;$$

$$\boldsymbol{p}_{\text{tot}} = \boldsymbol{p}_{\text{ext}} + \boldsymbol{p}_{\text{ext},E} = Y_E \boldsymbol{p} - \frac{4\pi}{3} R_{\text{cav}}^3 Y_E \left(\varepsilon - \varepsilon_0\right) \boldsymbol{E}_{\text{im}}\,. \tag{103}$$

The quadrupolar factors in these formulae stand for:

$$f_E = \frac{2 g_p}{2 g_p + 9\xi^2 + 9\xi^3}\,;\; f_p = \frac{2 + 8\xi}{2 g_p + 9\xi^2 + 9\xi^3}\,;\; g_p = 1 + 4\xi + 9\xi^2 + 9\xi^3\,;\; \xi = L_Q/R_{\text{cav}}. \tag{104}$$

Further, the electric field inside the cavity is the sum of the cavity field produced by $\boldsymbol{E}_{\text{im}}$ and the reaction field[36]:

$$\boldsymbol{E}_{\text{cav}} = Y_E \boldsymbol{E}_{\text{im}}\,,$$

$$\boldsymbol{E}_{\text{react}} = X_p \boldsymbol{p}\,, \quad X_p = \frac{1}{2\pi\varepsilon_0 R_{\text{cav}}^3} \frac{\varepsilon - f_p \varepsilon_0}{2\varepsilon + f_p \varepsilon_0}$$

$$\boldsymbol{E}_{\text{tot}} = X_p \boldsymbol{p} + Y_E \boldsymbol{E}_{\text{im}}\,. \tag{105}$$

On the other hand, eq. (21) relates the image field $\boldsymbol{E}_{\text{im}}$ and the total dipole moment $\boldsymbol{p}_{\text{tot}}$ of the ensemble cavity+molecule:

$$\boldsymbol{E}_{\text{im}} = -k_{\text{im}}^{\text{L}} \frac{p_{\text{tot},x}\mathbf{e}_x + p_{\text{tot},y}\mathbf{e}_y + 2 p_{\text{tot},z}\mathbf{e}_z}{\left|2 z_{\text{o}}\right|^3}\,. \tag{106}$$

Finally, eq. (20) relates the dipole moment $\boldsymbol{p}$ of the central molecule to its unperturbed (vacuum) value:

$$\boldsymbol{p} = \boldsymbol{p}_0 + \alpha_p \boldsymbol{E}_{\text{tot}}\,. \tag{107}$$

The vector equations (103)-(107) are solved for $\boldsymbol{E}_{\text{tot}}$, $\boldsymbol{p}$, $\boldsymbol{E}_{\text{im}}$, and $\boldsymbol{p}_{\text{tot}}$:

$$\boldsymbol{p} = \boldsymbol{p}_0 + \alpha_p X_p \boldsymbol{p} + \alpha_p Y_E \boldsymbol{E}_{\text{im}} \;\rightarrow\; \boldsymbol{p} = \frac{\boldsymbol{p}_0 + \alpha_p Y_E \boldsymbol{E}_{\text{im}}}{1 - \alpha_p X_p}\,;$$

$$\boldsymbol{p}_{\text{tot}} = \frac{Y_E}{1 - \alpha_p X_p} \boldsymbol{p}_0 + \alpha_{p,\text{cav}} \boldsymbol{E}_{\text{im}}\,, \text{ where } \alpha_{p,\text{cav}} = \frac{\alpha_p Y_E^2}{1 - \alpha_p X_p} - \frac{4\pi}{3} R_{\text{cav}}^3 \left(\varepsilon - \varepsilon_0\right) Y_E\,;$$

$$\boldsymbol{E}_{\text{im}} = -k_{\text{im}}^{\text{L}} \frac{\boldsymbol{p}_{\text{tot}} + p_{\text{tot},z}\mathbf{e}_z}{\left|2 z_{\text{o}}\right|^3}\,.$$

Hence, for the components of the total dipole moment, the image field and the molecule's dipole moment we obtain:

$$p_{\text{tot},x} = \frac{Y_E}{1-\alpha_p X_p}\frac{p_{0,x}}{1+k_{\text{im}}^{\text{L}}\alpha_{p,\text{cav}}/|2z_{\text{o}}|^3}, \quad p_{\text{tot},x} = \frac{Y_E}{1-\alpha_p X_p}\frac{p_{0,x}}{1+2k_{\text{im}}^{\text{L}}\alpha_{p,\text{cav}}/|2z_{\text{o}}|^3};$$

$$E_{\text{im},x} = -\frac{k_{\text{im}}^{\text{L}}Y_E}{|2z_{\text{o}}|^3}\frac{1}{1-\alpha_p X_p}\frac{p_{0,x}}{1+k_{\text{im}}^{\text{L}}\alpha_{p,\text{cav}}/|2z_{\text{o}}|^3}, \quad E_{\text{im},z} = -\frac{2k_{\text{im}}^{\text{L}}Y_E}{|2z_{\text{o}}|^3}\frac{1}{1-\alpha_p X_p}\frac{p_{0,z}}{1+2k_{\text{im}}^{\text{L}}\alpha_{p,\text{cav}}/|2z_{\text{o}}|^3};$$

$$p_x = \frac{1-\dfrac{k_{\text{im}}^{\text{L}}Y_E}{|2z_{\text{o}}|^3}\dfrac{4\pi}{3}R_{\text{cav}}^3(\varepsilon-\varepsilon_0)}{1+k_{\text{im}}^{\text{L}}\alpha_{p,\text{cav}}/|2z_{\text{o}}|^3}\frac{p_{0,x}}{1-\alpha_p X_p}; \quad p_z = \frac{1-\dfrac{2k_{\text{im}}^{\text{L}}Y_E}{|2z_{\text{o}}|^3}\dfrac{4\pi}{3}R_{\text{cav}}^3(\varepsilon-\varepsilon_0)}{1+2k_{\text{im}}^{\text{L}}\alpha_{p,\text{cav}}/|2z_{\text{o}}|^3}\frac{p_{0,z}}{1-\alpha_p X_p}. \tag{108}$$

At large distance, the last formula simplifies to:

$$\boldsymbol{p} = \frac{\boldsymbol{p}_0}{1-\alpha_p X_p},$$

i.e. Onsager's formula for the polarization of water in the liquid phase[36].

For the energy of the entity cavity+molecule placed in the image potential, we use *eq. 46* from ref. [37] but with ½ as in eq. (12) (the entity is the source of the image potential):

$$u = -\frac{1}{2}\boldsymbol{p}_{\text{ext}}\cdot\boldsymbol{E}_{\text{im}} + \text{O}(E_{\text{im}}^2) = -\frac{1}{2}Y_E\boldsymbol{p}\cdot\boldsymbol{E}_{\text{im}} = -\frac{1}{2}Y_E\frac{\boldsymbol{p}_0}{1-\alpha_p X_p}\cdot\boldsymbol{E}_{\text{im}}.$$

Substituting here eq. (108) leads to:

$$u_{pp}^{\text{L}} = \frac{k_{\text{im}}^{\text{L}}}{2}\frac{U_{pp,\text{cav}}^{\text{L}}}{|2z_{\text{o}}|^3},$$

with U_{pp} coefficient given by eq. (29). This result generalizes eq. (25) for polarizable point dipole to a polarizable dipole in a cavity.

In the gas phase, we can write:

$$U_{pp,\text{cav}}^{\text{G}} = \frac{Y_E^2}{(1-\alpha_p X_p)^2}\left(\frac{p_{0,x}^2+p_{0,y}^2}{1-k_{\text{im}}^{\text{G}}\alpha_{p,\text{cav}}/|2z_{\text{o}}|^3} + \frac{2p_{0,z}^2}{1-2k_{\text{im}}^{\text{G}}\alpha_{p,\text{cav}}/|2z_{\text{o}}|^3}\right). \tag{109}$$

The coefficient Y_E approaches 1 and X_p approaches 0 deep into the gas phase, but close to the surface, they may be significant – see Figure 2.

7.2. Image *pq* force in the liquid phase

The ***pq*** cross-interaction is dealt with similarly. The image gradient formula (90) truncated at z_o^{-4} reads:

$$\nabla \boldsymbol{E}_{\text{im}}(\boldsymbol{r}_{\text{o}}) = + \frac{3\boldsymbol{p}_{\text{im}}\mathbf{e}_z + 3\mathbf{e}_z\boldsymbol{p}_{\text{im}} + 3p_{\text{im},z}\left(\mathbf{U} - 5\mathbf{e}_z\mathbf{e}_z\right)}{4\pi\varepsilon^{\text{L}}\left|2z_{\text{o}}\right|^4} + \text{O}(z_{\text{o}}^{-5}).$$

Using eq. (6) for the relationship between $\boldsymbol{p}_{\text{im}}$ and $\boldsymbol{p}_{\text{tot}}$, and eq. (8) for the relationship between $\boldsymbol{q}_{\text{im}}$ and $\boldsymbol{q}_{\text{tot}}$, we obtain:

$$\nabla \boldsymbol{E}_{\text{im}}(\boldsymbol{r}_{\text{o}}) = +k_{\text{im}}^{\text{L}} \frac{3\boldsymbol{p}_{\text{tot}}\mathbf{e}_z + 3\mathbf{e}_z\boldsymbol{p}_{\text{tot}} - 3p_{\text{tot},z}\left(\mathbf{U} - \mathbf{e}_z\mathbf{e}_z\right)}{\left|2z_{\text{o}}\right|^4}. \tag{110}$$

For the image field, we truncate eq. (11) at z_o^{-4}:

$$\boldsymbol{E}_{\text{im}} = \frac{3p_{\text{im},z}\mathbf{e}_z - \boldsymbol{p}_{\text{im}}}{4\pi\varepsilon^{\text{L}}\left|2z_{\text{o}}\right|^3} + \frac{15q_{\text{im},zz}\mathbf{e}_z - 6\boldsymbol{q}_{\text{im}}\cdot\mathbf{e}_z}{8\pi\varepsilon^{\text{L}}\left|2z_{\text{o}}\right|^4} + \text{O}(z_{\text{o}}^{-5}).$$

Using eq. (6) for the relationship between $\boldsymbol{p}_{\text{im}}$ and $\boldsymbol{p}_{\text{tot}}$, and eq. (8) for the relationship between $\boldsymbol{q}_{\text{im}}$ and $\boldsymbol{q}_{\text{tot}}$, we obtain:

$$\boldsymbol{E}_{\text{im}} = -k_{\text{im}}^{\text{L}} \frac{\boldsymbol{p}_{\text{tot}} + p_{\text{tot},z}\mathbf{e}_z}{\left|2z_{\text{o}}\right|^3} + k_{\text{im}}^{\text{L}} \frac{6\boldsymbol{q}_{\text{tot}}\cdot\mathbf{e}_z + 3q_{\text{tot},zz}\mathbf{e}_z}{2\left|2z_{\text{o}}\right|^4}. \tag{111}$$

The external dipole moment formulae (103) and the reaction and cavity field formulae (105) hold; so does eq. (107) that relates the central dipole $\boldsymbol{p}$ to its unpolarized value $\boldsymbol{p}_0$. A similar set of equations hold for the quadrupoles and the field gradients[37,38]; the external quadrupole moments are given by:

$$\boldsymbol{q}_{\text{ext}} = Y_{\nabla E}\boldsymbol{q}, \quad Y_{\nabla E} = \frac{5f_{\nabla E}\varepsilon}{3\varepsilon + 2f_q\varepsilon_0};$$

$$\boldsymbol{q}_{\text{ext},\nabla E} = -\frac{8\pi}{15}R_{\text{cav}}^5 Y_{\nabla E}A_{\nabla E}\nabla\boldsymbol{E}_{\text{im}};$$

$$\boldsymbol{q}_{\text{tot}} = Y_{\nabla E}\boldsymbol{q} - \frac{8\pi}{15}R_{\text{cav}}^5 Y_{\nabla E}A_{\nabla E}\nabla\boldsymbol{E}_{\text{im}}. \tag{112}$$

The quadrupolar factors in these formulae stand for the expressions:

$$A_{\nabla E} = \left(1 + 9\xi^2\frac{g_{\nabla E}}{2g_q}\right)\varepsilon - \left(1 - 3\xi^2\frac{1 + 3\xi + 3\xi^2}{g_q}\right)\varepsilon_0;$$

$$f_q = \frac{1 + 6\xi + 6\xi^2}{g_q + 12\xi^2 + 18\xi^3 + 18\xi^4}, \quad f_{\nabla E} = \frac{g_q}{g_q + 12\xi^2 + 18\xi^3 + 18\xi^4};$$

$$g_q = 1 + 6\xi + 24\xi^2 + 54\xi^3 + 54\xi^4; \quad g_{\nabla E} = 1 + 9\xi + 39\xi^2 + 90\xi^3 + 90\xi^4. \tag{113}$$

For the reaction and cavity field gradients, the following formulae hold[37,38]:

$$\nabla\boldsymbol{E}_{\text{cav}} = Y_{\nabla E}\boldsymbol{E}_{\text{im}},$$

$$\nabla\boldsymbol{E}_{\text{react}} = X_q\boldsymbol{q}, \quad X_q = \frac{9}{4\pi\varepsilon_0 R_{\text{cav}}^5}\frac{\varepsilon - f_q\varepsilon_0}{3\varepsilon + 2f_q\varepsilon_0};$$

$$\nabla\boldsymbol{E}_{\text{tot}} = X_q\boldsymbol{q} + Y_{\nabla E}\nabla\boldsymbol{E}_{\text{im}}. \tag{114}$$

Finally, the central quadrupole is quadrupolarized by the total field gradient in the cavity:

$$\boldsymbol{q} = \boldsymbol{q}_0 + \alpha_q \nabla \boldsymbol{E}_{\text{tot}} .$$

These equations can be simplified to:

$$\boldsymbol{p} = \frac{\boldsymbol{p}_0 + \alpha_p Y_E \boldsymbol{E}_{\text{im}}}{1 - \alpha_p X_p} ; \quad \boldsymbol{q} = \frac{\boldsymbol{q}_0 + \alpha_q Y_{\nabla E} \nabla \boldsymbol{E}_{\text{im}}}{1 - \alpha_q X_q} .$$

Hence, the total dipole and quadrupole are given by:

$$\boldsymbol{p}_{\text{tot}} = \boldsymbol{p}_{\text{tot},0} + \alpha_{p,\text{cav}} \boldsymbol{E}_{\text{im}} ;$$

$$\boldsymbol{q}_{\text{tot}} = \boldsymbol{q}_{\text{tot},0} + \alpha_{q,\text{cav}} \nabla \boldsymbol{E}_{\text{im}} , \tag{115}$$

$$\text{where } \alpha_{q,\text{cav}} = \frac{\alpha_q Y_{\nabla E}^2}{1 - \alpha_q X_q} - \frac{8\pi}{15} R_{\text{cav}}^5 A_{\nabla E} Y_{\nabla E} ,$$

$$\boldsymbol{p}_{\text{tot},0} = \frac{Y_E}{1 - \alpha_p X_p} \boldsymbol{p}_0 , \quad \text{and} \quad \boldsymbol{q}_{\text{tot},0} = \frac{Y_{\nabla E}}{1 - \alpha_q X_q} \boldsymbol{q}_0 . \tag{116}$$

Here, $\alpha_{q,\text{cav}}$ is the quadrupolarizability, $p_{\text{tot},0}$ – the total dipole, and $\boldsymbol{q}_{\text{tot},0}$ – the total quadrupole of the assembly molecule+cavity in the absence of field. Eq. (110),(111)&(115) are two vectorial and two tensorial linear equations for $\boldsymbol{p}_{\text{tot}}$, $\boldsymbol{q}_{\text{tot}}$, $\boldsymbol{E}_{\text{im}}$ and $\nabla \boldsymbol{E}_{\text{im}}$. We solve and we neglected all terms that do not produce correction beyond $O(z_o^{-6})$:

$$p_{\text{tot},x} \approx \frac{Y_E}{1 - \alpha_p X_p} \frac{p_{0,x}}{1 + k_{\text{im}}^{\text{L}} \alpha_{p,\text{cav}} / |2z_o|^3} ; \quad p_{\text{tot},z} \approx \frac{Y_E}{1 - \alpha_p X_p} \frac{p_{0,z}}{1 + 2k_{\text{im}}^{\text{L}} \alpha_{p,\text{cav}} / |2z_o|^3} ;$$

$$\boldsymbol{q}_{\text{tot}} \approx \frac{Y_{\nabla E}}{1 - \alpha_q X_q} \boldsymbol{q}_0 . \tag{117}$$

The energy of the ensemble multipole+cavity interacting with its image field can be written as:

$$u = -\frac{1}{2} \boldsymbol{p}_{\text{tot},0} \cdot \boldsymbol{E}_{\text{im}} - \frac{1}{4} \boldsymbol{q}_{\text{tot},0} : \nabla \boldsymbol{E}_{\text{im}} =$$

$$= \frac{k_{\text{im}}^{\text{L}}}{2} \left(\frac{p_{\text{tot},0,x} p_{\text{tot},x} + p_{\text{tot},0,y} p_{\text{tot},y} + 2 p_{\text{tot},0,z} p_{\text{tot},z}}{|2z_o|^3} - \frac{6 p_{\text{tot},x} q_{\text{tot},xz} + 6 p_{\text{tot},y} q_{\text{tot},yz} + 9 p_{\text{tot},z} q_{\text{tot},zz}}{|2z_o|^4} \right) + O(z_o^{-5}).$$

The substitution of eq. (117) and (116) here and simplification produce the final result (31) for the coefficient $U_{pq,\text{cav}}$.

7.3. Average values of the dipole moments

In linear approximation, the average dipole per cavity + molecule is:

$$\bar{p}^{\mathrm{L}}_{\mathrm{tot},z} = \int_0^{\pi}\int_0^{2\pi}\int_0^{2\pi} p_{\mathrm{tot},z}\rho_{\varphi\psi\theta}\sin\theta \mathrm{d}\varphi \mathrm{d}\psi \mathrm{d}\theta =$$

$$= \frac{Y_E^2 Y_{\nabla E}}{\left(1-\alpha_p X_p\right)^2\left(1-\alpha_q X_q\right)} \frac{k^{\mathrm{L}}_{\mathrm{im}}}{1+2k^{\mathrm{L}}_{\mathrm{im}}\alpha_{p,\mathrm{cav}}/\left|2z_{\mathrm{o}}^3\right|} \frac{3q_{\mathrm{n},zz}p_0^2}{40kT}\frac{1}{z_{\mathrm{o}}^4}, \tag{118}$$

where $p_{z,\mathrm{tot}}$ is given by eq. (117) and $\rho_{\varphi\psi\theta}$ – by the linearized eq. (41), but this time with energy coefficients from eq. (29)&(31) with polarization and cavity and reaction fields. Instead of eq. (42) and (100), in this case, the average energy is:

$$\bar{u}^{\mathrm{L}} = \frac{2}{3}\frac{Y_E^2}{\left(1-\alpha_p X_p\right)^2} k^{\mathrm{L}}_{\mathrm{im}} \frac{1+\frac{3}{2}k^{\mathrm{L}}_{\mathrm{im}}\alpha_p/\left|2z_{\mathrm{o}}\right|^3}{\left(1+2k^{\mathrm{L}}_{\mathrm{im}}\alpha_p/\left|2z_{\mathrm{o}}\right|^3\right)\left(1+k^{\mathrm{L}}_{\mathrm{im}}\alpha_p/\left|2z_{\mathrm{o}}\right|^3\right)} \frac{p_0^2}{\left|2z_{\mathrm{o}}\right|^3} + \frac{12}{5}k^{\mathrm{L}}_{\mathrm{im}}\frac{\boldsymbol{q}:\boldsymbol{q}}{\left|2z_{\mathrm{o}}\right|^5}. \tag{119}$$

8. Local values of the dielectric properties (ε, L_Q and R_{cav})

To calculate the local dielectric permittivity and quadrupolar length of water, we use the quadrupolar variant of the Onsager cavity model[38]. This model gives the following formulae for the macroscopic polarizability α_P and quadrupolarizability α_Q:

$$\alpha_P \equiv \quad \varepsilon - \varepsilon_0 = \frac{Y_E}{1-\alpha_p X_p}\left(\alpha_p + \frac{1}{1-\alpha_p X_p}\frac{p_0^2}{3k_{\mathrm{B}}T}\right)C\,; \tag{120}$$

$$\alpha_Q \equiv \quad 3\varepsilon L_Q^2 = \frac{Y_{\nabla E}}{1-\alpha_q X_q}\left(\alpha_q + \frac{1}{1-\alpha_q X_q}\frac{\mathbf{q}_0 : \mathbf{q}_0}{10k_{\mathrm{B}}T}\right)C\,. \tag{121}$$

Here, the reaction and cavity field factors are given by eq. (30), with the quadrupolar coefficients from eq. (104) and (113); C is the local concentration of water as set by the tanh formula (57); α_q is the molecular quadrupolarizability of water.

Eq. (120)&(121) are two nonlinar equations for ε and L_Q; to solve them, however, we need to know the value of R_{cav}, because $X_p \sim 1/R_{\text{cav}}^3$ and $X_q \sim 1/R_{\text{cav}}^5$, see eq. (30); the quadrupolar factors are also functions of R_{cav}. The cavity size is a function of the local density. To deal with this dependence, we will assume two limiting models for it. In ref. [38], we found from experimental data that, for liquid water at various pressures (density in the range 1000-1100 kg/m^3), the cavity radius follows the empirical correlation:

$$\frac{m}{\frac{4}{3}\pi R_{\text{cav}}^3} = k_\rho \rho + k_{298}\,, \tag{122}$$

where $k_\rho = 0.1195$, $k_{298} = 2538.6$ kg/m^3; m – molecular mass of water. If we extrapolate this formula to low densities, it produces nearly constant R_{cav}, see Figure S 3. This is clearly unrealistic for gas-like densities, but it sets a lower limit for this quantity.

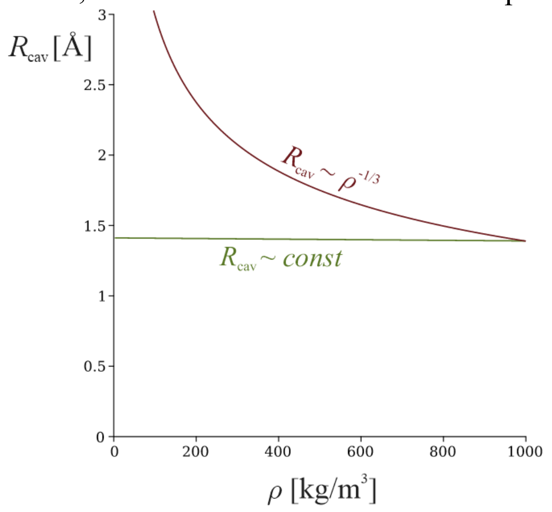

Figure S 3. Local radius of the cavity R_{cav} as a function of the local mass density ρ of water. We use two limits: one where R_{cav} changes little (green) and another where it follows the Onsager formula (red).

For low densities, eventually the cavity radius should approach Onsager's formula[36] $1/R_{\text{cav}}^3 \propto \rho$. We know the value of $R_{\text{cav,bulk}}$ for the bulk density ρ_{bulk} from eq. (122); hence, we can write this formula as:

$$\frac{R_{\text{cav,bulk}}^3}{R_{\text{cav}}^3} = \frac{\rho}{\rho_{\text{bulk}}}. \qquad (123)$$

Here, ρ and R_{cav} are local values near the interface; $\rho_{\text{bulk}} = 997$ kg/m^3; $R_{\text{cav,bulk}} = 1.39$ Å. Eq. (122) and (123) are compared in Figure S 3; the first option sets a lower limit and the second – an upper limit for R_{cav}.

Eq. (120),(121)&(122), or alternatively eq. (120),(121)&(123), are three algebraic equations for three unknowns: ε, L_Q, and R_{cav}. These are functions of the local mass density ρ (where $\rho = mC$). The solutions are plotted in Figure S 4. As seen, if R_{cav} follows eq. (122) (i.e. it is approximately constant), then ε is approximately linear function of C, as obvious from eq. (120). When $\varepsilon \propto C$, then the second eq. (121) predicts that L_Q is nearly constant until very low densities. Hence, for $R_{\text{cav}} \sim const$, the surface layer is of permittivity proportional to the local density but of unchanged quadrupolar length compared to the bulk.

In contrast, if Onsager's eq. (123) is used, ε is dropping much faster as the density decreases, i.e. the surface layer is of greatly reduced permittivity. On the other hand, the macroscopic quadrupolarizability from eq. (122) is still approximately linear with C (because α_q is small). Since $L_Q \sim \alpha_Q/\varepsilon$, the quadrupolar length increases in the surface layer (because the interphase is much less polar, and L_Q is a ratio between quadupolar and dipolar strengths[1]). Only at very low densities, where ε approaches 1, will L_Q drop to zero. This is the reason for the maximum of L_Q vs ρ in Figure S 4 – i.e. the surface layer behaves as more quadrupolar than the very polar bulk phase.

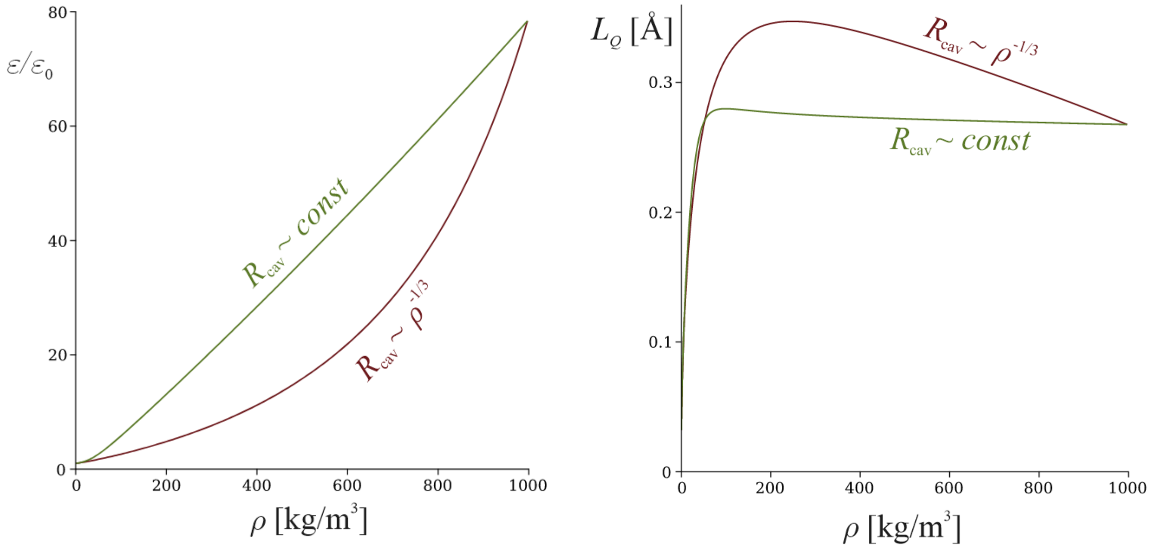

Figure S 4. Local relative dielectric permittivity (left) and quadrupolar length (right) as a function of the local mass density.

Using the tanh formula for C, we can convert the dependence on density to dependence on distance from the surface; hence, Figure S 4 produces Figure S 5 through eq. (57). We plot ε and L_Q as functions of the distance $z - L_\varepsilon$ to the equimolecular surface, rather than directly z to the dielectric surface.

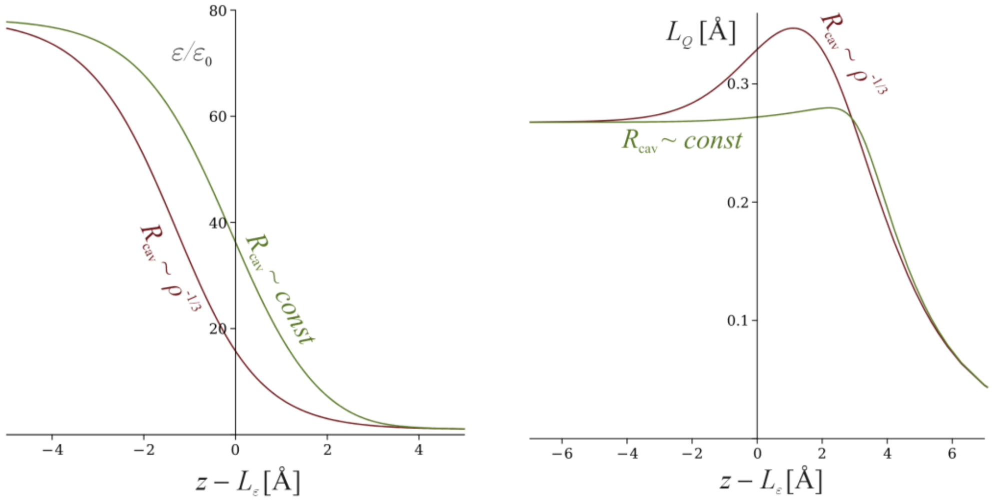

Figure S 5. Local relative dielectric permittivity (left) and quadrupolar length (right) as a function of the distance from the equimolecular surface.

From Figure S 5, we can calculate the distance between the equimolecular and the dielectric surfaces using the Gibbs formula:

$$L_\varepsilon = \frac{\left| \int_{-\infty}^{0} \varepsilon - \varepsilon^{\mathrm{L}} \mathrm{d}z_{\mathrm{e}} + \int_{0}^{\infty} \varepsilon - \varepsilon^{\mathrm{G}} \mathrm{d}z_{\mathrm{e}} \right|}{\varepsilon^{\mathrm{L}} - \varepsilon^{\mathrm{G}}}. \qquad (124)$$

Direct integration of the curves in Figure S 5 gives $L_\varepsilon = 0.22$ Å for the lower assumed limit for R_{cav} (constant), and $L_\varepsilon = -1.33$ Å for the higher R_{cav} (Onsager's formula).

9. Molecular model of the surface polarizabilities

Consider a solid dipole that is in electric field $\boldsymbol{E}_{\text{im}} + \boldsymbol{E}$, where $\boldsymbol{E}$ is a small external field. The energy of the dipole is given by:

$$u = -\frac{1}{2}\boldsymbol{p}\cdot\boldsymbol{E}_{\text{im}} - \boldsymbol{p}\cdot\boldsymbol{E} + \frac{1}{2}\frac{(\boldsymbol{p}-\boldsymbol{p}_0)^2}{\alpha_p}, \text{ where} \tag{125}$$

$$\boldsymbol{p} = \boldsymbol{p}_0 + \alpha_p(\boldsymbol{E}_{\text{im}} + \boldsymbol{E}). \tag{126}$$

Here, E_{im} is created by $\boldsymbol{p}$ itself, therefore the coefficient ½. Substituting eq. (126) into (125) and neglecting the small E^2 terms, we obtain:

$$u \approx -\frac{1}{2}\boldsymbol{p}_0\cdot\boldsymbol{E}_{\text{im}} - \boldsymbol{p}_0\cdot\boldsymbol{E}\,.$$

The first term corresponds to the energy (24)-(25) in the absence of an external field ($\boldsymbol{E} = 0$) and in this section we will call it u_{im}. The Boltzmann distribution linearized with respect to the small $\boldsymbol{E}$ reads:

$$\rho = c_{\text{n}}\mathrm{e}^{-u_{\text{im}}/kT}\left(1+\frac{\boldsymbol{p}_0\cdot\boldsymbol{E}}{kT}\right).$$

The normalizing coefficient is given by:

$$c_{\text{n}} = \frac{1}{\int \mathrm{e}^{-u_{\text{im}}/kT}\,\mathrm{d}\Omega + \int \mathrm{e}^{-u_{\text{im}}/kT}\frac{\boldsymbol{p}_0\cdot\boldsymbol{E}}{kT}\mathrm{d}\Omega} = c_{\text{n},E=0}\left(1-\frac{\overline{\boldsymbol{p}}_{0,E=0}\cdot\boldsymbol{E}}{kT}\right).$$

Here, the index $\boldsymbol{E} = 0$ indicates value unperturbed by the external field.

The average dipole moment is given by:

$$\overline{\boldsymbol{p}} = c_{\text{n},E=0}\left(1-\frac{\overline{\boldsymbol{p}}_{0,E=0}\cdot\boldsymbol{E}}{kT}\right)\int\left[\boldsymbol{p}_0 + \alpha_p(\boldsymbol{E}_{\text{im}}+\boldsymbol{E})\right]\mathrm{e}^{-u_{\text{im}}/kT}\left(1+\frac{\boldsymbol{p}_0\cdot\boldsymbol{E}}{kT}\right)\mathrm{d}\Omega =$$

$$= \overline{\boldsymbol{p}}_{E=0} + \frac{\left(\overline{\boldsymbol{p}_0\boldsymbol{p}}_{E=0} - \overline{\boldsymbol{p}}_{E=0}\overline{\boldsymbol{p}}_{0\,E=0}\right)\cdot\boldsymbol{E}}{kT} + \alpha_p\boldsymbol{E} + \mathrm{O}(E^2).$$

The macroscopic polarization is:

$$\overline{\boldsymbol{P}}(z) = C(z)\left[\overline{\boldsymbol{p}}_{E=0}(z) + \left(\alpha_p\mathbf{U} + \frac{\overline{\boldsymbol{p}_0\boldsymbol{p}}_{E=0} - \overline{\boldsymbol{p}}_{0\,E=0}\overline{\boldsymbol{p}}_{E=0}}{kT}\right)\cdot\boldsymbol{E}(z)\right]. \tag{127}$$

This is the equation of state of a linear pyroelectric. The first term in the square brackets gives the polarization in the absence of external field. The round bracket gives a formula for what is known as the molecular polarizability [36,34] – however, for a pyroelectric. The tangential molecular polarizability is:

$$\alpha_{\text{mol,t}} = \alpha_p + \frac{\overline{p_{0x}p_x}_{E=0}}{kT} = \alpha_p + \frac{\overline{p_{0x}^2}_{E=0}}{kT\left(1+k_{\text{im}}^{\text{L}}\alpha_p/|2z_{\text{o}}|^3\right)}. \tag{128}$$

The normal one is given by:

$$\alpha_{\text{mol},z} = \alpha_p + \frac{\overline{p_{0z}p_z}_{E=0} - \overline{p}_{0z\,E=0}\overline{p}_{z\,E=0}}{kT} = \alpha_p + \frac{\overline{p_{0z}^2}_{E=0} - \overline{p}_{0z\,E=0}^2}{kT\left(1+2k_{\text{im}}^{\text{L}}\alpha_p/|2z_{\text{o}}|^3\right)}. \tag{129}$$

Far from the surface, in the absence of image forces, the two molecular polarizabilities simplify to the classical Debye expression:

$$\alpha_{\text{mol}} = \alpha_{\text{mol},z} = \alpha_p + \frac{p_0^2}{3kT}. \qquad (130)$$

10. Structure of the DDL with explicit distribution of the surface dipole moment

We tested several sets of parameter values for the quadupolar electrostatic problem (70). The base case was chosen to be $\xi_{\text{cutoff}} = ½$, and $L_\varepsilon = 0.24$ Å, respectively (see Table 3); we use $\varepsilon^{\text{L}} = 78.4\times\varepsilon_0$, $L_Q^{\text{L}} = 0.27$ Å at $T = 25$ °C, as obtained from our dielectric cavity model[38,37]; $C^{\text{L}} = 3.33\times10^{28}$ m^{-3} (density 997 kg/m^3); $L_C = 1$ Å, see eq. (58). We also tested the values for $\xi_{\text{cutoff}} = 1/3$, and higher values of L_Q^{L} up to 0.5 Å, to check the uncertainty of the final value of the surface potential. We also investigated a larger gap L_ε for $\alpha_P(z)$, as the polarizability in eq. (70) is normal and the surface of zero α_z^{S} is below the dielectric surface, see eq. (65). Hence, instead of eq. (82), for α_P we used:

$$\alpha_P = \frac{\varepsilon^{\text{L}} - \varepsilon_0}{2}\left(1 - \tanh\frac{z - L_{\alpha z}}{2L_C}\right),$$

where the length $L_{\alpha z}$ stands for the quantity $L_{\alpha z} = -(½ \pm ¼)L_{\text{imm}}$ in eq. (65).

Figure S 6 shows the solution for the electric field in the surface layer of eq. (70), but for a different cutoff parameter, $\xi_{\text{cutoff}} = ½$, compared to the base case in Figure 6. The difference in the unperturbed dipole moment density P_0 in Figure S 6 and Figure 6 is clearly visible, but the produced field is not very different due to the smoothing effect of the quadrupolar term in eq. (70).

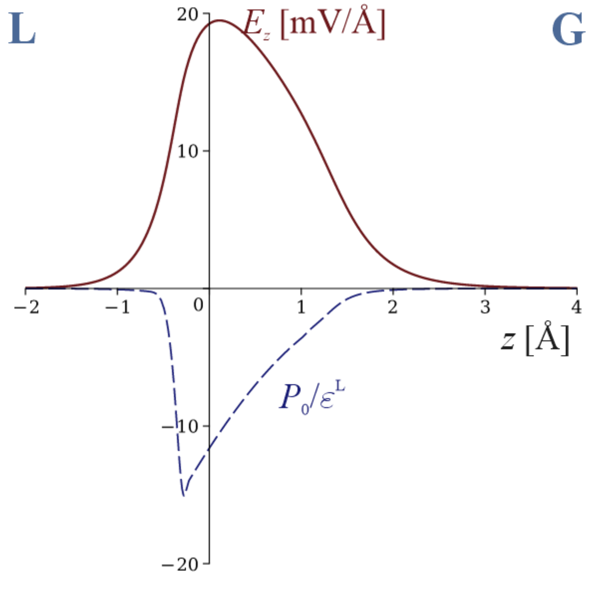

Figure S 6. Field profile in the DDL for profile of $P_0(z)$ corresponding to $\xi_{\text{cutoff}} = 1/3$. The field profile changes little with the base case $\xi_{\text{cutoff}} = 1/2$ (see Figure 6).

Table S 1 shows the value of the surface potential calculated from the distribution of E_z through eq. (83) for various choices of the parameters of the quadrupolar equation of electrostatics (70). As seen, $\Delta_{\mathrm{L}}^{\mathrm{G}}\phi$ is weakly dependent on the choice of ξ_{cutoff} and L_Q^{L} (in the range of values deemed reasonable for them, the potential remains within ±2 mV of the base value $\Delta_{\mathrm{L}}^{\mathrm{G}}\phi$ = -33 mV). The structural parameters of the surface (L_ε, L_C, $L_{\alpha z}$) have a more pronounced effect (±5 mV).

Table S 1. The first row is the base case; the underlined values in the following lines are the parameter values that we changed compared to the base case.

ξ_{cutoff}	L_ε, $L_{\alpha z}$	L_Q^{L} [Å]	L_C [Å]	$\Delta_{\mathrm{L}}^{\mathrm{G}}\phi$ [mV]
½	L_ε = 0.24 Å	0.27	1.0	-33
1/3	L_ε = 0.32 Å	0.27	1.0	-34
½	L_ε = 0.24 Å	**0.5**	1.0	-34
**1/3**	**L_ε = 0.32 Å**	**0.5**	1.0	-35
½	L_ε = 0.24 Å	0.27	**1.5**	-31
½	**L_ε = 0 Å**	0.27	1.0	-29
½	L_ε = 0.24 Å<br>**$L_{\alpha z}$ = -0.1 Å**	0.27	1.0	-35
½	L_ε = 0.24 Å<br>**$L_{\alpha z}$ = -0.3 Å**	0.27	1.0	-40

Additional references

56. R. Slavchov. On the theory of charged heterogeneous interfaces: electric interactions in Langmuir monolayers and semiconductor surfaces. Thesis, 2007, Sofia University, ch. 7.
